# Supplementary material for: Pan-cancer analysis of longitudinal metastatic tumors reveals genomic alterations and immune landscape dynamics associated with pembrolizumab sensitivity
Source: Nat Commun. 2021 Aug 26;12:5137. doi: 10.1038/s41467-021-25432-7 (PMC8390680; doi:10.1038/s41467-021-25432-7)
Supplement: Supplementary file 1 — Supplementary Information [file 41467_2021_25432_MOESM1_ESM.pdf]

Supplementary Information

Table of Contents

- Supplementary Discussion
- Supplementary References
- Supplementary Figures 1 - 11
- Supplementary Tables 1 – 2
- Supplementary Note: INSPIRE Clinical Study Protocol

Supplementary Discussion

Enrichment of mutations with predicted functional effects in tumors with high and low pembrolizumab sensitivity

We first used SIFT[9] and PolyPhen2[10] to predict the functional impact of the non-synonymous mutations in each tumor. We selected mutations predicted to be "deleterious" by SIFT and "probably damaging" by PolyPhen2 for the subsequent enrichment analysis. We identified one gene, *LRP1B*, frequently mutated in the HS/CB group (7 of 19, $p < 0.05$ Fisher's exact test). In melanoma and non-small cell lung cancer patients, *LRP1B* mutations have been associated with high TMB and prolonged survival with ICB treatment[11]. In our cohort, we observed *LRP1B* mutations only in melanoma and basal cell carcinoma patients. Due to this association between *LRP1B* mutations and cancer type, we do not have sufficient evidence to report *LRP1B* mutations as a pan-cancer biomarker candidate for predicting immune checkpoint blockade response. When we repeated the IO resistance gene analysis, we still did not observe any significant mutation enrichment. We expected this result since we had a limited number of mutations within the genes of interest prior to the functional prediction filter.

TMB and PGA as combined biomarker to predict pembrolizumab clinical response

To evaluate tumor genome mutation and copy number burden as a combined biomarker to predict pembrolizumab clinical outcome, we divided the patients into four subgroups, based on the combination of high (>10 mutations/Mb) or low TMB and high (> 50%) or

low PGA (Supplementary Fig. 6). We then calculated the proportion of patients with clinical benefit (CBR) and frequencies of cancer types and pembrolizumab sensitivity types within each subgroup. PGA is low (< 50%) in all six TMB-high tumors (Supplementary Fig. 6). The TMB-low and PGA-low group contains the fewest tumors with high pembrolizumab sensitivity and more than half of the breast and ovarian cancers. Based on published studies, we expected the highest clinical benefit rate (CBR) in TMB-high and PGA-low tumors and lowest in TMB-low and PGA-high tumors. Indeed, the CBR is 1.9-times higher in the TMB-high and PGA-low group as compared to the overall CBR (43%, 26/61), while CBR is 3.3-times lower in the TMB-low and PGA-low group (Supplementary Fig. 6). The proportion of patients with clinical benefit is notably higher in the subgroup of TMB-high and PGA-low (5 out of 6) compared to the subgroup of TMB-low and PGA-high (2 out of 15) patients (p = 0.006, Fisher's exact test) (Supplementary Fig. 6). Our data validated the potential utility of TMB and PGA as a combinatorial biomarker to increase the pool of patients and broaden the range of cancer types that will meaningfully respond to pembrolizumab.

Supplementary References

1. Bratman, S. V. *et al.* Personalized circulating tumor DNA analysis as a predictive biomarker in solid tumor patients treated with pembrolizumab. *Nature Cancer* **1**, 873–881 (2020).
2. Clouthier, D. L. *et al.* A technical feasibility report on correlative studies from the investigator-initiated phase II study of pembrolizumab (Pembro) immunological response evaluation (INSPIRE). *J. Clin. Orthod.* **35**, 11607–11607 (2017).
3. Cerami, E. *et al.* The cBio cancer genomics portal: an open platform for exploring multidimensional cancer genomics data. *Cancer Discov.* **2**, 401–404 (2012).
4. Gao, J. *et al.* Integrative analysis of complex cancer genomics and clinical profiles using the cBioPortal. *Sci. Signal.* **6**, l1 (2013).
5. Samstein, R. M. *et al.* Tumor mutational load predicts survival after immunotherapy across multiple cancer types. *Nat. Genet.* **51**, 202–206 (2019).
6. Zehir, A. *et al.* Mutational landscape of metastatic cancer revealed from prospective clinical sequencing of 10,000 patients. *Nat. Med.* **23**, 703–713 (2017).

7. Robinson, D. R. *et al.* Integrative clinical genomics of metastatic cancer. *Nature* **548**, 297–303 (2017).
8. Ru, B. *et al.* TISIDB: an integrated repository portal for tumor-immune system interactions. *Bioinformatics* **35**, 4200–4202 (2019).
9. Ng, P. C. & Henikoff, S. SIFT: Predicting amino acid changes that affect protein function. *Nucleic Acids Res.* **31**, 3812–3814 (2003).
10. Adzhubei, I., Jordan, D. M. & Sunyaev, S. R. Predicting functional effect of human missense mutations using PolyPhen-2. *Curr. Protoc. Hum. Genet.* **Chapter 7**, Unit7.20 (2013).
11. Chen, H. *et al.* Association of LRP1B Mutation With Tumor Mutation Burden and Outcomes in Melanoma and Non-small Cell Lung Cancer Patients Treated With Immune Check-Point Blockades. *Front. Immunol.* **10**, 1113 (2019).
12. Riaz, N. et al. Tumor and Microenvironment Evolution during Immunotherapy with Nivolumab. Cell **171**, 934–949.e15 (2017).
13. Mariathasan, S. et al. TGFβ attenuates tumour response to PD-L1 blockade by contributing to exclusion of T cells. Nature **554**, 544–548 (2018).
14. Snyder, A. et al. Contribution of systemic and somatic factors to clinical response and resistance to PD-L1 blockade in urothelial cancer: An exploratory multi-omic analysis. PLoS Med. **14**, e1002309 (2017).
15. Hugo, W. et al. Genomic and Transcriptomic Features of Response to Anti-PD-1 Therapy in Metastatic Melanoma. Cell vol. 165 35–44 (2016).
16. Miao, D. et al. Genomic correlates of response to immune checkpoint therapies in clear cell renal cell carcinoma. Science **359**, 801–806 (2018).

Supplementary Fig. 1

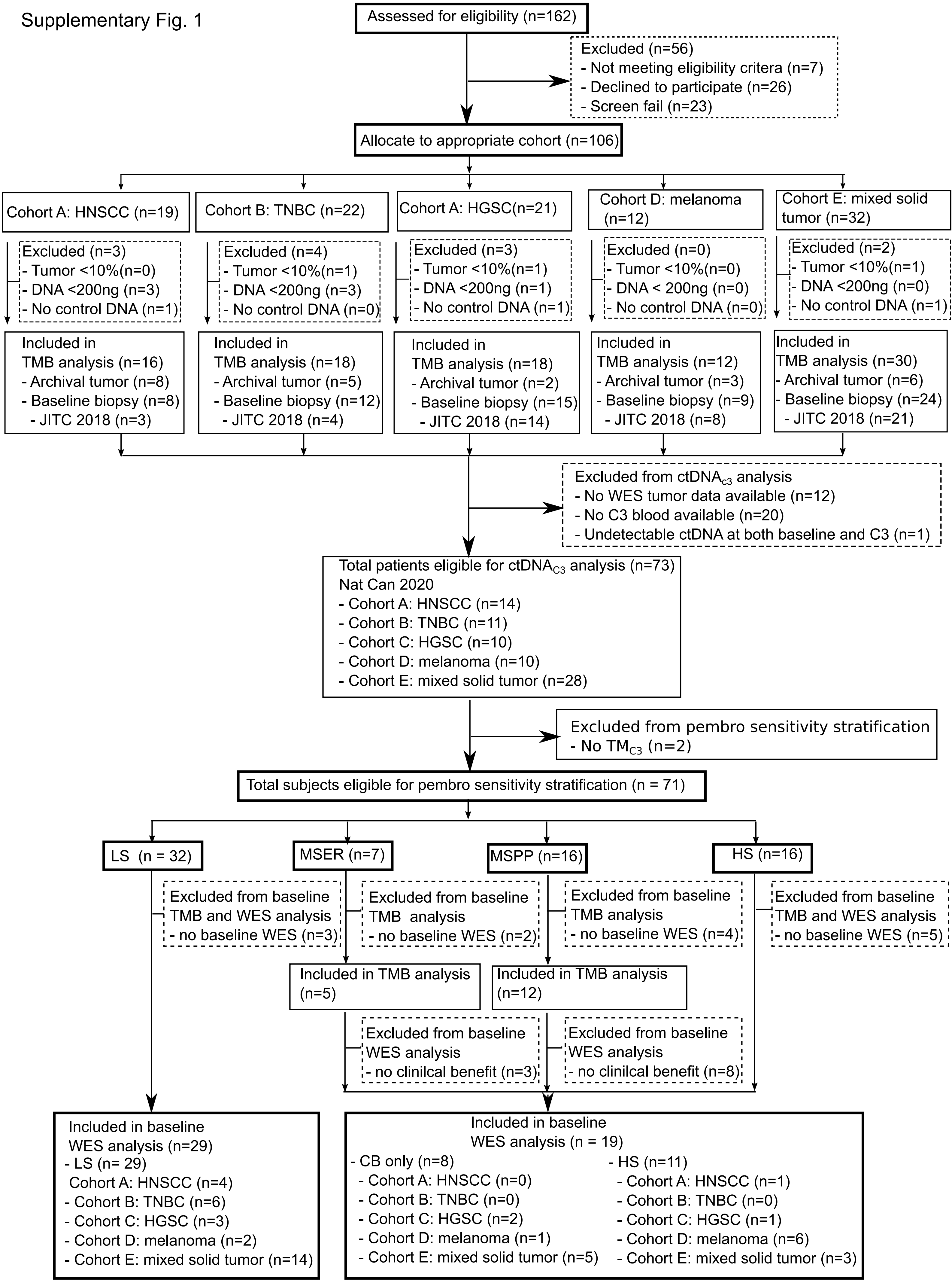

Supplementary Fig.1 Consort diagram of the INSPIRE clinical trial. The diagram summarizes the detailed break-down of sample sizes and tumor types included in the current and previously published studies[1,2]. HNSCC: Head and Neck Squamous Cell Cancer; TNBC: Triple-Negative Breast Cancer; HGSC: High-grade Serous Ovarian Cancer; $ctDNA_{C3}$: change in circulating tumor DNA at cycle 3 from baseline; TM_{C3}: change in tumor measurement/burden at cycle 3 from baseline; TMB: Tumor Mutation Burden; WES: Whole Exome Sequencing; CB: Clinical Benefit. Pembrolizumab molecular sensitivity groups: LS: Low-Sensitivity; MSER: Mixed-Sensitivity with Emerging Resistance; MSPP: Mixed-sensitivity with Potential Pseudoprogression; HS: High-Sensitivity.

Supplementary Fig. 2

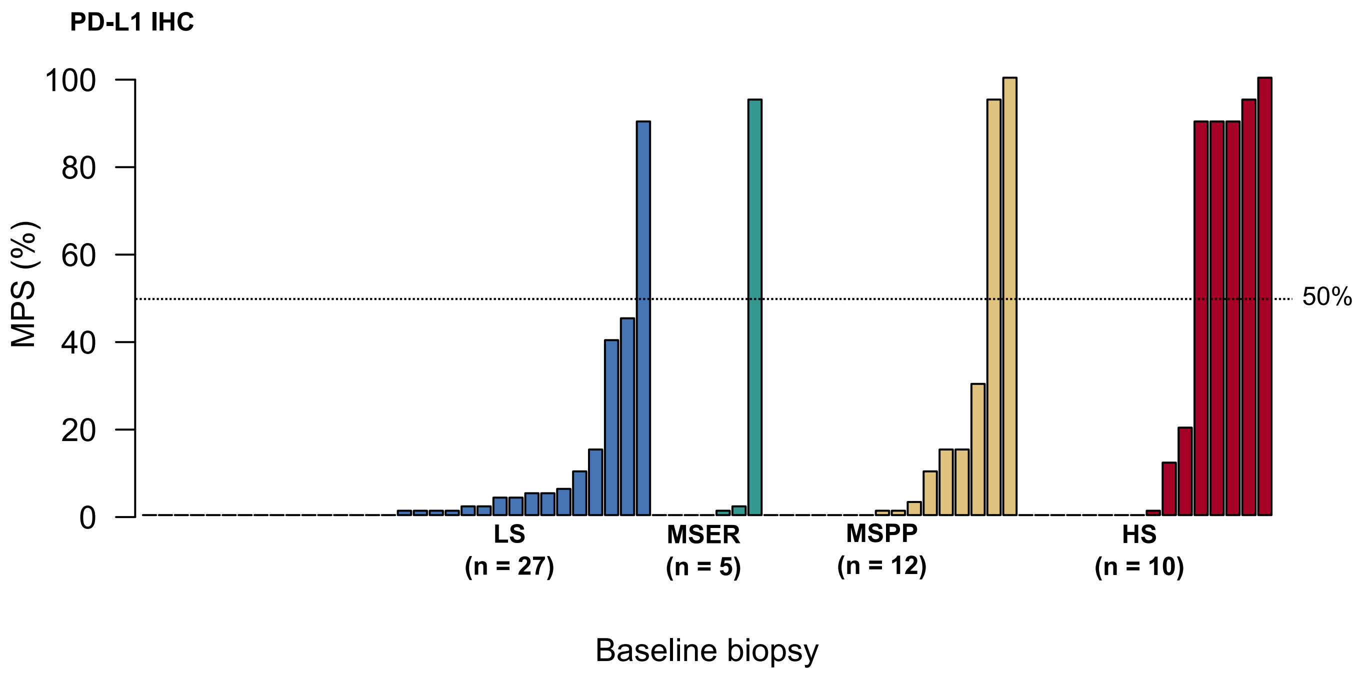

Supplementary Fig.2 PD-L1 protein expression in baseline tumor biopsies. Barplot for each tumor specimen grouped by pembrolizumab molecular sensitivity subgroups and sorted in ascending order of PD-L1 protein expression score assessed by immunohistochemistry. A horizontal dotted line indicates the 50% biomarker threshold as reported in previous studies. Source data are provided in SourceData_SupplementaryFig2.xlsx. MPS: Modified Proportion Score.

Supplementary Fig. 3

A

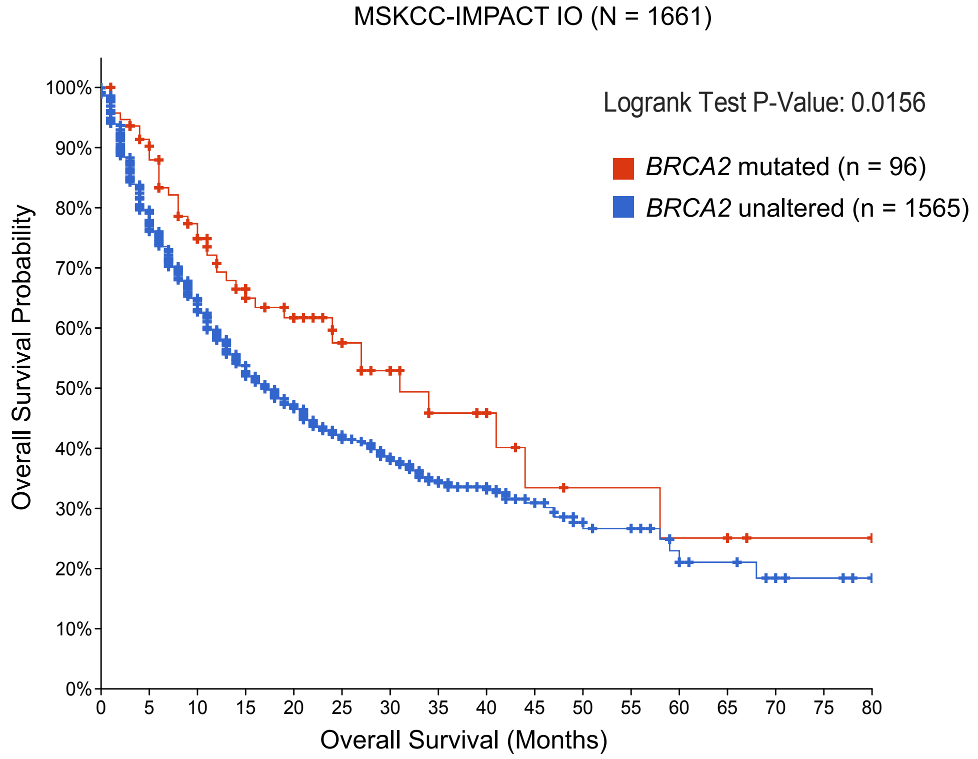

B

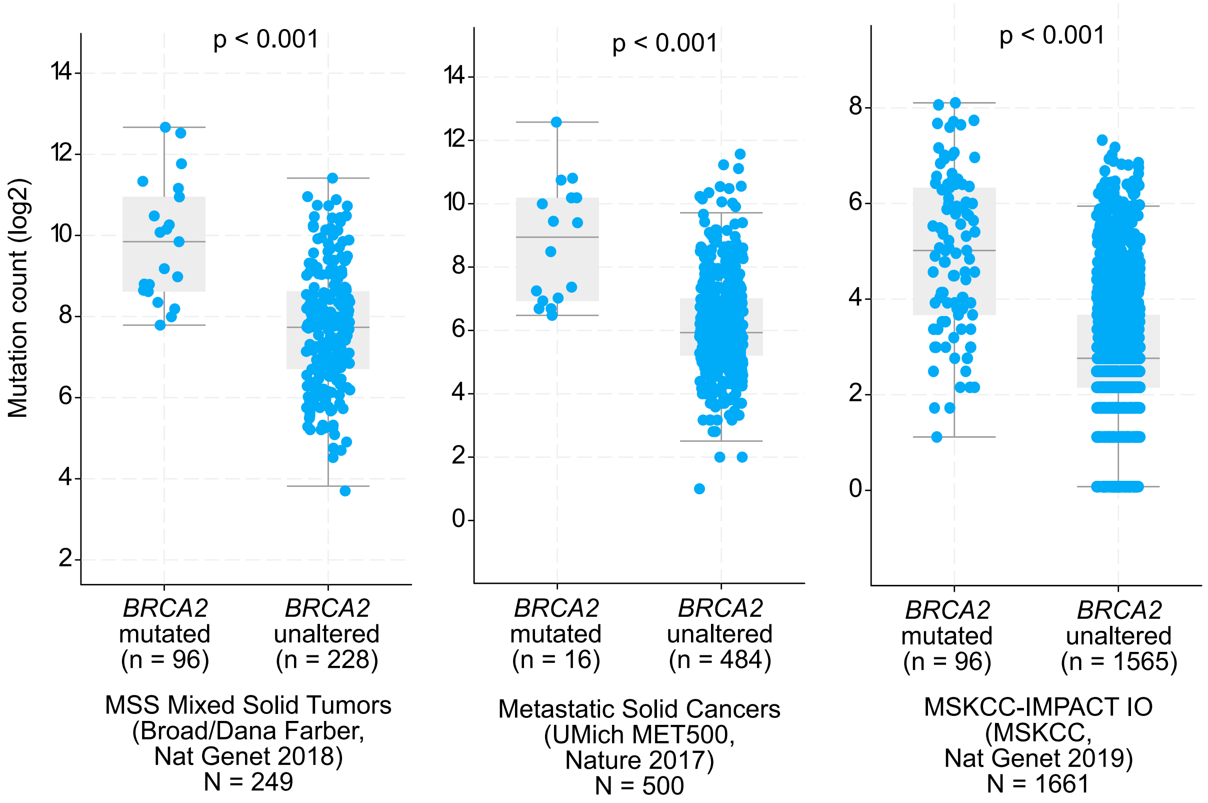

Supplementary Fig.3 Association of *BRCA2* mutation status with checkpoint immunotherapy survival and tumor mutation burden in solid cancers. Publicly available data from three previously published studies of mutation profiling in solid cancers were accessed via cBioportal[3,4]. **A.** Kaplan-Meier comparison of overall survival in patients with or without *BRCA2* mutated solid tumors treated with checkpoint immunotherapy (MSKCC-IO dataset)[5]. **B.** Boxplots comparing tumor mutation burden between tumors with and without *BRCA2* somatic point mutation in three independent solid tumor datasets: MSS mixed solid tumors from Broad/Dana Farber[6] (two-sided Wilcoxon Test, $p = 1.75 \times 7^{-7}$), metastatic solid cancers from UMich MET500[7] (two-sided Wilcoxon Test, $p = 6.42 \times 10^{-7}$), and immunotherapy treated solid tumors from the MSKCC-IMPACT study[5] (two-sided Wilcoxon Test, $p < 1 \times 10^{-10}$),. P-values from two-sided Wilcoxon tests are reported for each comparison. The distance between the third-quartile (Q3) and first-quartile (Q1), known as the interquartile range (IQR), is marked around the median by a black rectangle. Vertical lines extending from the top and bottom of the rectangle show the maximum (Q3+1.5-times IQR) and minimum (Q1+1.5-times IQR). Source data are provided in SourceData_SupplementaryFig3.xlsx.

Supplementary Fig. 4

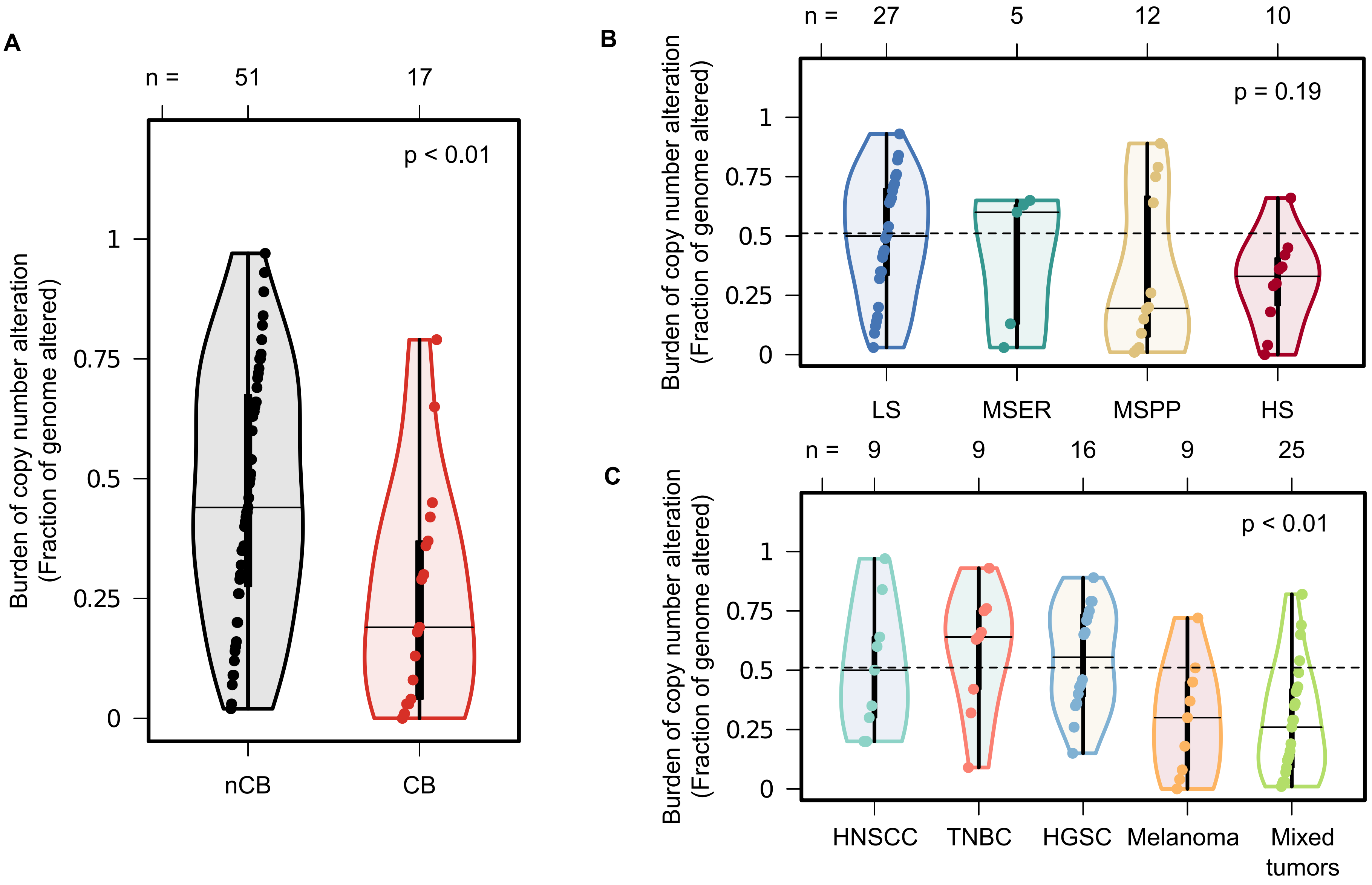

Supplementary Fig.4 Percent Genome Alteration (PGA) in INSPIRE baseline tumors. Violin plots comparing distributions of PGA in baseline tumors from: **A.** patients with and without clinical benefit (p = 0.0039), **B.** pembrolizumab molecular sensitivity groups, and **C.** cancer cohorts (p = 0.0024). Within each group, data points are sorted in increasing PGA and the median is indicated by a solid horizontal black line. P-values are calculated from two-sided Wilcoxon rank sum test for two-groups, and two-sided Kruskal-Wallis tests for more than two-groups. The dotted horizontal line shows the median PGA value across all cohorts. Source data are provided in SourceData_SupplementaryFig4.xlsx.

Supplementary Fig 5.

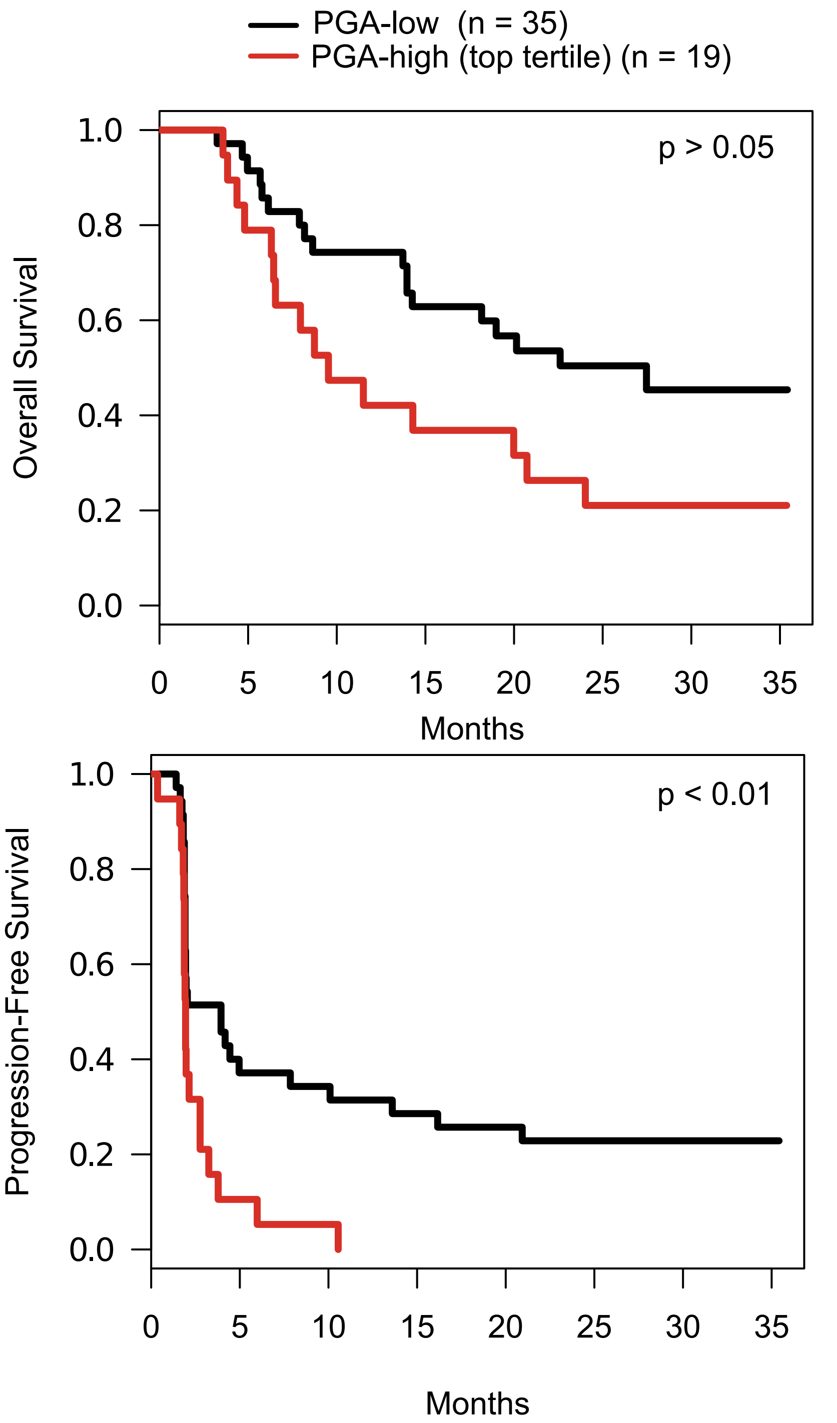

Supplementary Fig.5 Percent Genome Alteration (PGA) and patient survival. Kaplan-Meier plots comparing overall survival (p = 0.15) (top) and progression-free survival (p = 0.027) (bottom) between patients with PGA-high and PGA-low tumors. P-values indicate statistical significance from a two-sided log-rank test of a univariate Cox proportional hazards model. Source data are provided in SourceData_SupplementaryFig5.xlsx.

Supplementary Fig 6.

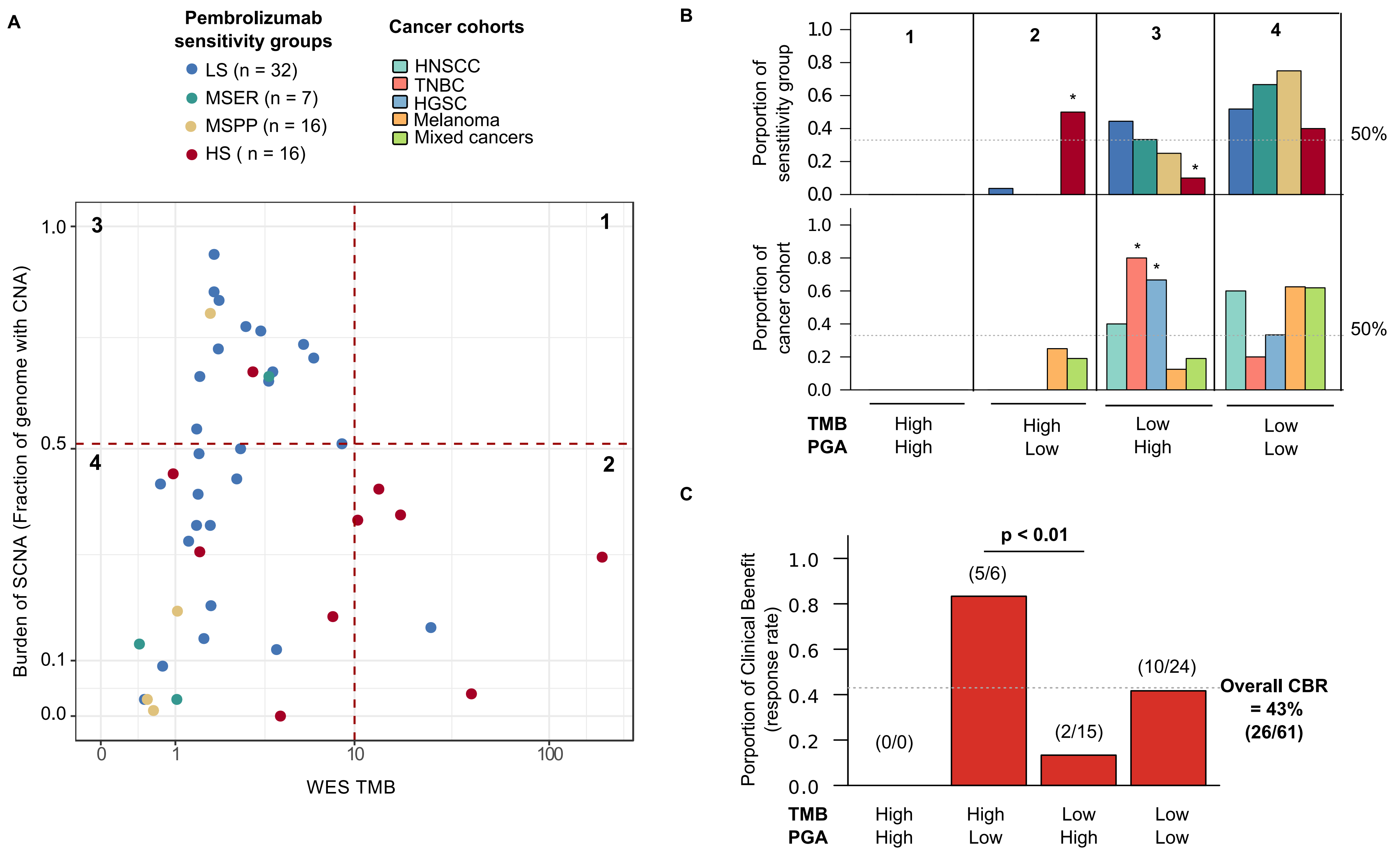

Supplementary Fig.6 Assessment of Percent Genome Alteration (PGA) and Tumor Mutation Burden (TMB) as a combined biomarker for pembrolizumab clinical benefit in solid tumors. **A.** Scatter plot of baseline tumor TMB and PGA. Dashed vertical and horizontal red lines indicate cut-offs to designate high versus low TMB or PGA groups, dividing subjects into four subgroups. Data points are colored according to membership in the pembrolizumab molecular sensitivity subgroups determined previously. Fisher's exact test $p = 0.006$. **B.** Barplot summarizing distribution of pembrolizumab molecular sensitivity subgroups (top) and cancer type cohorts (bottom) within each TMB/PGA subgroup. The height of each bar shows the fraction of samples within a specific sensitivity subgroup or cancer cohort assigned to a TMB/PGA subgroup. Statistical significance was evaluated by Fisher's Exact test. * $p < 0.05$. **C.** Barplot of clinical benefit rate of TMB/PGA determined subgroups. Sample numbers are displayed as fractions above each bar. Statistical significance was evaluated by Fisher's Exact test. The dashed horizontal grey line indicates the overall clinical benefit rate. Source data are provided in SourceData_SupplementaryFig6.xlsx.

Supplementary Fig 7.

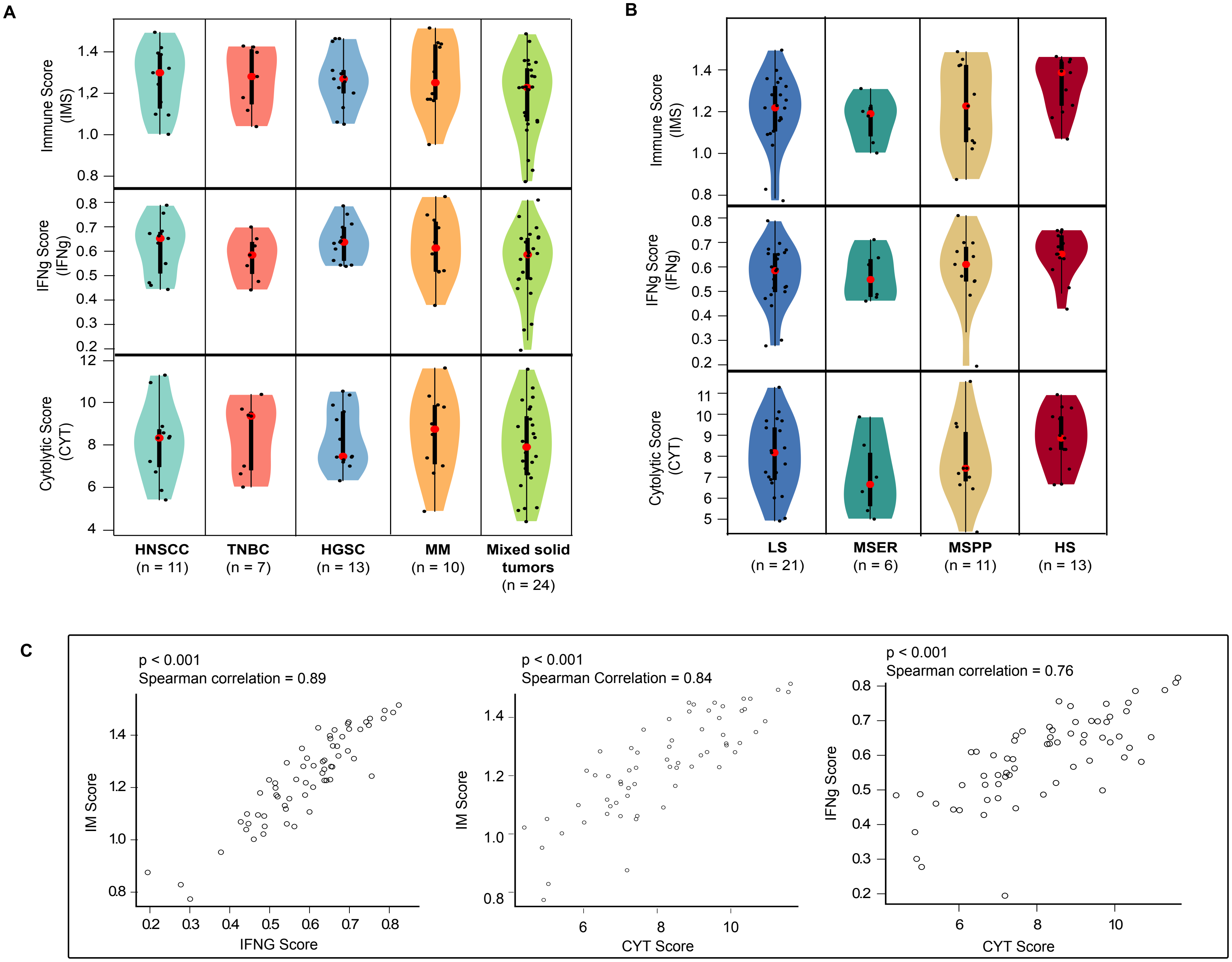

Supplementary Fig.7 Innate immune activity gene-expression signatures in solid tumors. Violin plots summarizing the distributions of immune score (IM, top), Interferon gamma score (IFNG, middle), and cytolytic activity score (CYT, bottom) of baseline tumors grouped by **A.** cancer type or **B.** pembrolizumab molecular sensitivity subgroup. In each group, the median is indicated by a red dot. **C.** Spearman correlation of pairwise comparisons between immune score ($p = 2.50 \times 10^{-16}$) (left), IFN gamma score ($p = 9.28 \times 10^{-13}$) (middle), and cytolytic activity ($p = 7.37 \times 10^{-11}$) (right). P value reflects a two-sided correlation test. For all violin plots, the distance between the third-quartile (Q3) and first-quartile (Q1), known as the interquartile range (IQR), is marked around the median by a black rectangle. Vertical lines extending from the top and bottom of the rectangle show the maximum (Q3+1.5-times IQR) and minimum (Q1+1.5-times IQR). Source data are provided in SourceData_SupplementaryFig7.xlsx.

Supplementary Fig. 8

p < 0.10 uncorrected for multiple testing

Signature	Upper 66%	CBR High	CBR Low	Clinical Benefit Odds Ratio (95% CI)	p	Overall Survival HR (95% CI)	p	Progression-Free Survival HR (95% CI)	p
IMS	1.31	36% (8/22)	23% (10/43)	1.87 (0.52,6.61)	0.38	0.61 (0.3,1.22)	0.161	0.74 (0.41,1.35)	0.326
CYT	9.04	32% (7/22)	26% (11/43)	1.35 (0.37,4.77)	0.77	0.79 (0.4,1.55)	0.492	1.01 (0.56,1.81)	0.976
IFNG	0.65	41% (9/22)	21% (9/43)	2.57 (0.73,9.27)	0.142	0.33 (0.15,0.72)	0.005	0.57 (0.31,1.04)	0.066
B.cells.naive	0.67	27% (6/22)	28% (12/43)	0.97 (0.25,3.46)	1	0.71 (0.36,1.41)	0.33	1 (0.56,1.78)	0.994
B.cells.memory	0.01	32% (7/22)	26% (11/43)	1.35 (0.37,4.77)	0.77	0.97 (0.5,1.9)	0.93	0.92 (0.51,1.65)	0.776
Plasma.cells	0.38	23% (5/22)	30% (13/43)	0.68 (0.16,2.51)	0.573	0.96 (0.5,1.86)	0.911	1.12 (0.63,1.99)	0.702
T.cells.CD8	3.85	36% (8/22)	23% (10/43)	1.87 (0.52,6.61)	0.38	0.5 (0.24,1.02)	0.058	0.79 (0.44,1.44)	0.45
T.cells.CD4.naive	0	25% (1/4)	28% (17/61)	0.86 (0.02,11.65)	1	0.75 (0.18,3.13)	0.695	0.99 (0.31,3.18)	0.982
T.cells.CD4.memory.resting	2.46	32% (7/22)	26% (11/43)	1.35 (0.37,4.77)	0.77	0.42 (0.2,0.89)	0.023	0.83 (0.46,1.5)	0.536
T.cells.CD4.memory.activated	0.4	32% (7/22)	26% (11/43)	1.35 (0.37,4.77)	0.77	0.52 (0.25,1.07)	0.074	0.92 (0.51,1.66)	0.772
Tfh	1.59	45% (10/22)	19% (8/43)	3.56 (1.01,13.25)	0.039	0.52 (0.26,1.05)	0.069	0.54 (0.29,0.99)	0.046
Tregs	0	35% (6/17)	25% (12/48)	1.62 (0.4,6.15)	0.53	0.39 (0.16,0.95)	0.037	0.68 (0.36,1.31)	0.251
Tgd	0	29% (6/21)	27% (12/44)	1.07 (0.27,3.83)	1	0.8 (0.4,1.62)	0.544	1.04 (0.58,1.88)	0.892
NK.cells.resting	0	25% (3/12)	28% (15/53)	0.85 (0.13,4.04)	1	1.18 (0.54,2.58)	0.679	1.21 (0.6,2.41)	0.596
NK.cells.activated	1.29	41% (9/22)	21% (9/43)	2.57 (0.73,9.27)	0.142	0.41 (0.19,0.87)	0.021	0.64 (0.35,1.17)	0.149
Monocytes	0.65	32% (7/22)	26% (11/43)	1.35 (0.37,4.77)	0.77	0.94 (0.48,1.85)	0.867	0.78 (0.43,1.42)	0.418
Macrophages.M0	1.15	27% (6/22)	28% (12/43)	0.97 (0.25,3.46)	1	1.23 (0.64,2.35)	0.536	0.91 (0.51,1.63)	0.75
Macrophages.M1	1.25	41% (9/22)	21% (9/43)	2.57 (0.73,9.27)	0.142	0.39 (0.19,0.84)	0.015	0.63 (0.34,1.15)	0.131
Macrophages.M2	4.21	27% (6/22)	28% (12/43)	0.97 (0.25,3.46)	1	0.66 (0.33,1.31)	0.232	0.81 (0.45,1.46)	0.474
Dendritic.cells.resting	0.17	27% (6/22)	28% (12/43)	0.97 (0.25,3.46)	1	0.82 (0.42,1.63)	0.58	1.04 (0.58,1.85)	0.896
Dendritic.cells.activated	0.17	23% (5/22)	30% (13/43)	0.68 (0.16,2.51)	0.573	1.19 (0.61,2.32)	0.616	0.99 (0.55,1.77)	0.96
Mast.cells.resting	0	40% (2/5)	27% (16/60)	1.81 (0.14,17.41)	0.611	1.31 (0.46,3.73)	0.612	0.84 (0.3,2.33)	0.732
Mast.cells.activated	5.18	23% (5/22)	30% (13/43)	0.68 (0.16,2.51)	0.573	0.94 (0.48,1.84)	0.848	1.03 (0.57,1.84)	0.928
Eosinophils	0.22	23% (5/22)	30% (13/43)	0.68 (0.16,2.51)	0.573	0.73 (0.37,1.45)	0.367	1.26 (0.71,2.22)	0.433
Neutrophils	0.18	14% (3/22)	35% (15/43)	0.3 (0.05,1.27)	0.085	2.04 (1.06,3.91)	0.033	1.45 (0.81,2.61)	0.208

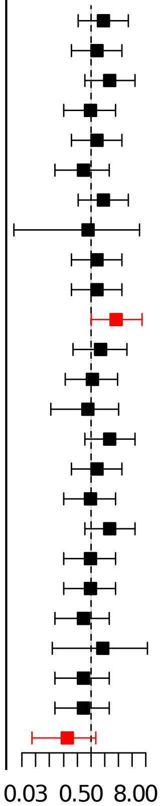

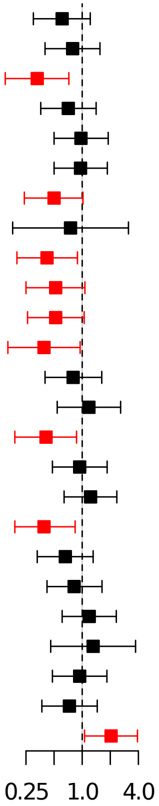

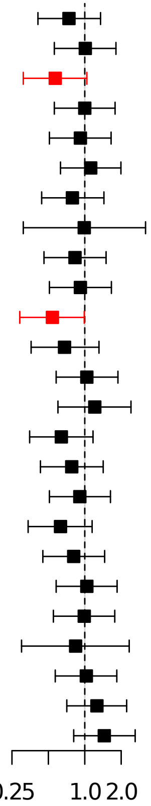

Supplementary Fig.8 Evaluation of associations between baseline immune activity gene-expression scores with clinical benefit and survival. Forest plot summaries of univariate associations between baseline tumor gene-expression derived immune activity/infiltrating immune cell signatures and clinical benefit, overall survival, and progression-free survival. For each signature, patients were divided into two groups defined by membership in the upper-tertile of the signature score distribution. Clinical benefit rate in the high and low groups were calculated and Fisher's exact test was performed to assess statistical significance of the observed differences. Univariate Cox proportional hazards models were used to evaluate the association with overall and progression-free survival. The location of either the odds ratio (success = high score group) or hazard ratio (event = progression/death) is shown as a solid dot with whiskers indicating the 95% confidence interval. P values are uncorrected for multiple testing. Source data are provided in SourceData_SupplementaryFig8.xlsx.

Supplementary Fig. 9

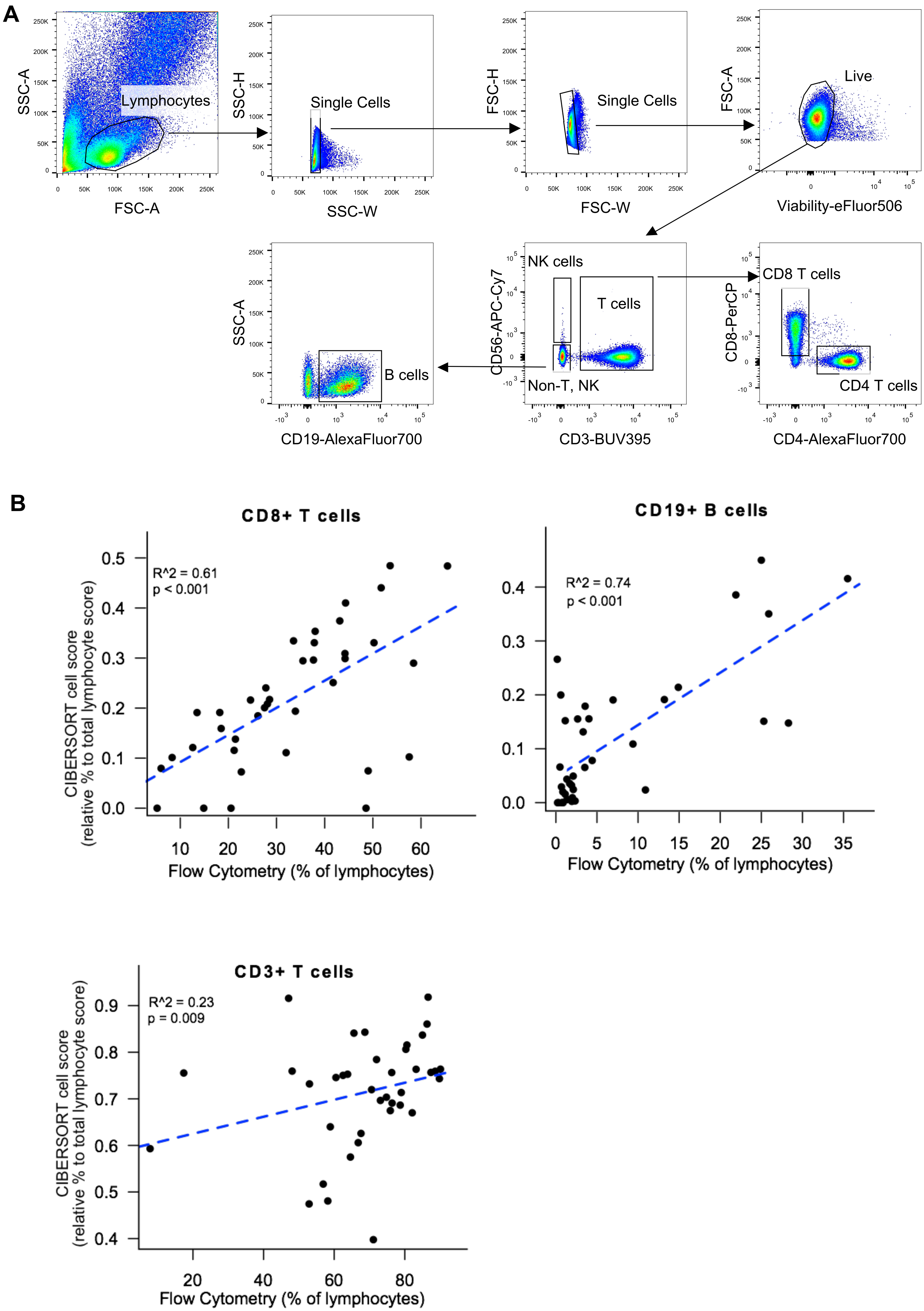

Supplementary Fig.9 Correlation of CIBERSORT estimated and flow-cytometry determined tumor infiltrating immune cell abundances. **A.** Flow cytometry gating strategy schematic for quantifying tumor infiltrating immune cell populations: CD3+ T cells, CD8+ T cells, CD19+ B cells. **B.** Correlations between CIBERSORT estimated quantities of CD3+ T cells, CD8+ T cells ($p = 2 \times 10^{-5}$), and CD19+ B cells ($p = 8.053 \times 10^{-8}$) with quantities measured by flow cytometry. Spearman correlation R-values are provided with p-values from two-sided correlation tests. Blue dotted lines indicate linear best fit between the X and Y variables. Source data are provided in SourceData_SupplementaryFig9.xlsx.

Supplementary Fig 10.

A

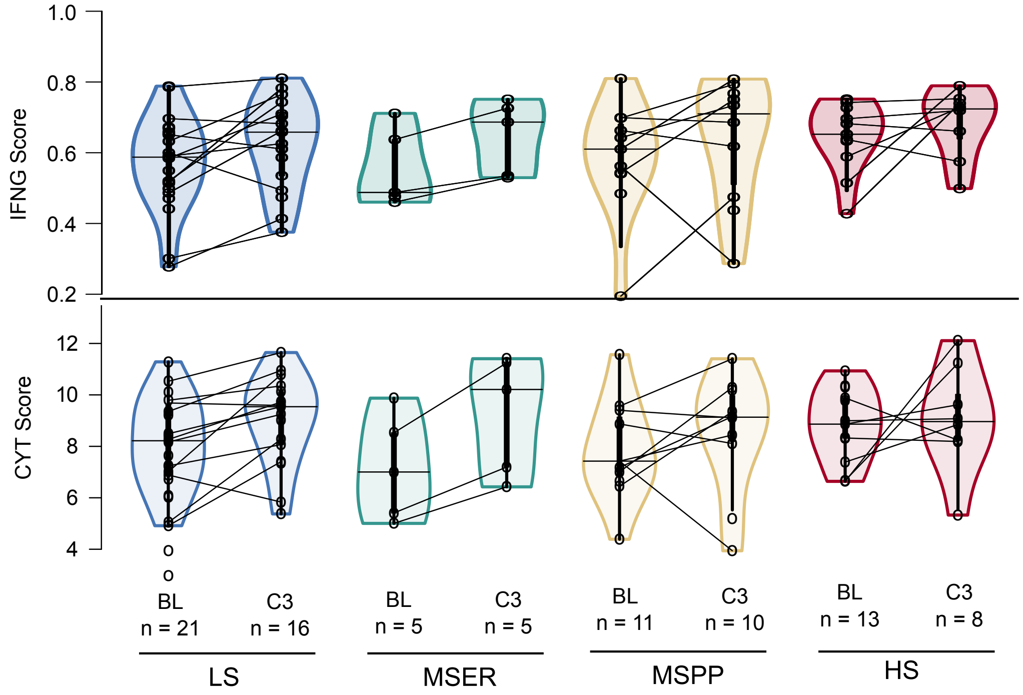

B

uncorrected p < 0.10

Signature	BL mean (range)	TX mean (range)	Delta-mean (95% CI)	p
IMS	1.24 (0.77-1.52)	1.29 (0.7-1.56)	0.07 (0,0.14)	0.063
CYT	8.16 (4.39-11.62)	8.91 (3.94-12.11)	0.85 (0.04,1.62)	0.04
IFNG	0.59 (0.19-0.82)	0.64 (0.29-0.81)	0.06 (0.01,0.1)	0.025
B.cells.naive	1.19 (0-10.3)	1.15 (0-8.6)	0 (-0.11,0.27)	0.749
B.cells.memory	0.23 (0-2.21)	0.28 (0-4.12)	0 (0,0)	0.447
Plasma.cells	0.5 (0-3.53)	0.4 (0-3.7)	-0.03 (-0.15,0.04)	0.305
T.cells.CD8	2.92 (0-13.99)	4.53 (0-19.25)	0.86 (-0.04,1.88)	0.07
T.cells.CD4.naive	0.04 (0-1.39)	0.07 (0-2.77)	0 (0,0)	0.393
T.cells.CD4.memory.resting	2.72 (0-13.14)	3.31 (0-9.17)	0.66 (-0.14,1.56)	0.097
T.cells.CD4.memory.activated	0.79 (0-8.51)	0.98 (0-7.11)	0 (0,0)	0.609
Tfh	1.41 (0.07-7.35)	2.01 (0-6.15)	0.61 (0.17,1.13)	0.01
Tregs	0.04 (0-0.5)	0.08 (0-1.24)	0 (0,0)	0.711
Tgd	0.18 (0-4.73)	0.73 (0-7.21)	0 (0,0)	0.097
NK.cells.resting	0.1 (0-1.44)	0.12 (0-3.21)	0 (0,0)	0.231
NK.cells.activated	1.18 (0-5.85)	1.81 (0-12.06)	0.31 (-0.05,0.7)	0.1
Monocytes	0.64 (0-3)	1.13 (0-21.33)	-0.01 (-0.18,0.14)	0.738
Macrophages.M0	1.1 (0-7.68)	1.35 (0-5.15)	0.03 (-0.01,0.59)	0.315
Macrophages.M1	1.16 (0-5.91)	1.52 (0-5.96)	0.35 (-0.02,0.8)	0.086
Macrophages.M2	3.6 (0.47-11.89)	3.7 (0-12.74)	-0.37 (-1.29,0.6)	0.473
Dendritic.cells.resting	0.33 (0-3.49)	0.27 (0-2.76)	0 (-0.04,0)	0.452
Dendritic.cells.activated	0.3 (0-4.86)	0.21 (0-1.67)	0 (-0.03,0)	0.448
Mast.cells.resting	0.23 (0-6.81)	0.12 (0-1.55)	0 (0,0)	0.306
Mast.cells.activated	4.57 (0-16.64)	4.38 (0-11.85)	-0.19 (-1.44,1.08)	0.769
Eosinophils	0.22 (0-1.56)	0.35 (0-1.77)	0.1 (0,0.19)	0.035
Neutrophils	0.2 (0-1.43)	0.36 (0-5.77)	0 (-0.03,0.02)	0.955

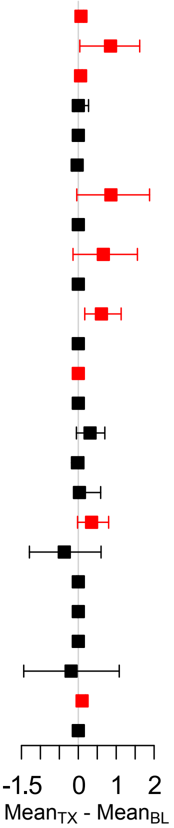

Supplementary Fig.10 Pharmacodynamic effects of pembrolizumab on tumor immune activity and infiltrating immune cell abundances. **A.** Violin plots of IFNg score (top) and CYT score (bottom) at baseline and treatment cycle 3 grouped by pembrolizumab molecular sensitivity. Data points collected from the same patient are connected by a solid black line between baseline and cycle 3. The median of each group is indicated with a solid horizontal line. The distance between the third-quartile (Q3) and first-quartile (Q1), known as the interquartile range (IQR), is marked around the median by a black rectangle. Vertical lines extending from the top and bottom of the rectangle show the maximum (Q3+1.5-times IQR) and minimum (Q1+1.5-times IQR). **B.** Forest plot summaries of comparisons of CIBERSORT inferred infiltrating immune cell abundances before and after pembrolizumab treatment. Statistical significance of group differences were evaluated with paired two-sided Wilcoxon rank sum tests between samples collected from the same patient. The location of the difference between the mean of the two groups is shown as a solid dot with whiskers indicating the 95% confidence interval for each signature. P values are uncorrected for multiple testing. Source data are provided in SourceData_SupplementaryFig10.xlsx. CYT: cytolytic score.

Supplementary Fig. 11

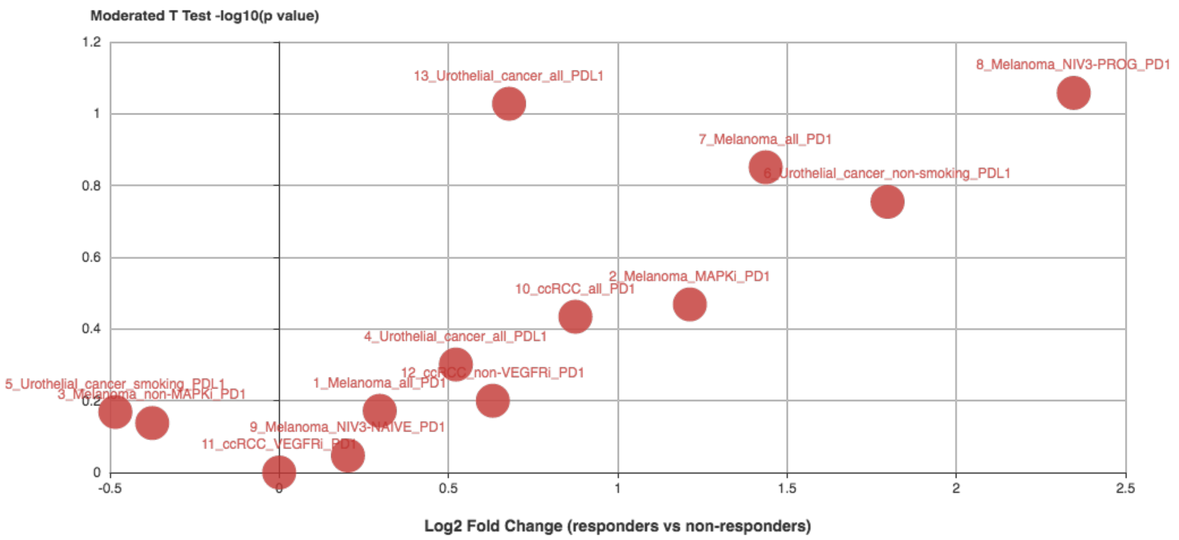

No	PMID	Cancer type	Group	Drug	# Res	# NRes	Log2 (Fold Change)	P value
8	29033130	Melanoma	NIV3-PROG	Anti-PD-1 (nivolumab)	15	11	2.347	0.0875
13	29443960	Urothelial cancer	all	Anti-PD-L1 (atezolizumab)	68	230	0.679	0.0938
7	29033130	Melanoma	all	Anti-PD-1 (nivolumab)	26	23	1.437	0.141
6	28552987	Urothelial cancer	non-smoking	Anti-PD-L1 (atezolizumab)	4	7	1.797	0.176
2	26997480	Melanoma	MAPKi	Anti-PD-1 (pembrolizumab and nivolumab)	6	5	1.213	0.34
10	29301960	Clear cell renal cell carcinoma (ccRCC)	all	Anti-PD-1 (nivolumab)	4	8	0.875	0.368
4	28552987	Urothelial cancer	all	Anti-PD-L1 (atezolizumab)	9	16	0.522	0.5
12	29301960	Clear cell renal cell carcinoma (ccRCC)	non-VEGFRi	Anti-PD-1 (nivolumab)	2	8	0.631	0.631
1	26997480	Melanoma	all	Anti-PD-1 (pembrolizumab and nivolumab)	14	12	0.297	0.673
5	28552987	Urothelial cancer	smoking	Anti-PD-L1 (atezolizumab)	5	9	-0.484	0.678
3	26997480	Melanoma	non-MAPKi	Anti-PD-1 (pembrolizumab and nivolumab)	8	7	-0.375	0.728
9	29033130	Melanoma	NIV3-NAIVE	Anti-PD-1 (nivolumab)	11	12	0.203	0.897
11	29301960	Clear cell renal cell carcinoma (ccRCC)	VEGFRi	Anti-PD-1 (nivolumab)	2	0	0	1

Supplementary Fig.11 Validation of *PLA2G2D* expression in publically available checkpoint immunotherapy datasets on the TISIDB: an integrated repository portal for tumor-immune system interactions[8] web portal. The scatter plot indicates the gene expression difference of *PLA2G2D* between responder and non-responders in each published study [PMIDs: 29033130[12], 29443960[13], 28552987[14], 26997480[15], 29301960[16]] listed in the table above. Source data are provided in SourceData_SupplementaryFig11.xlsx.

SUPPLEMENTARY TABLE 1 - CURATED IMMUNE RESPONSE RELATED GENES AND COHORT MUTATION FREQUENCIES

Hugo Symbol	Pathway	Mechanism	Pro or anti-tumor TME	Direction of alteration	Feature_1	Feature_2	n_mutated_Feature1	n_mutated_Feature2	FDR	n_loh_Feature1	n_loh_Feature2
MSH2	P1: DNA damage repair	Defects in DNA repair results in accumulation of tumor antigens that	immune recognition/ activation	LOF	LS	CB.HS	NA	NA	NA		
MSH6					LS	CB.HS	NA	NA	NA		
MLH1					LS	CB.HS	2 of 27	1 of 19	1.0000		
PMS2					LS	CB.HS	0 of 27	2 of 19	0.1652		
POLE					LS	CB.HS	2 of 27	2 of 19	1.0000		
BRCA2					LS	CB.HS	0 of 27	5 of 19	0.0085		
KRAS	P2: MAPK pathway	Activation of MAPK signaling reduces TILs and pro-inflammatory	immune evasion	GOF	LS	CB.HS	NA	NA	NA		
NRAS					LS	CB.HS	NA	NA	NA		
STK11					LS	CB.HS	NA	NA	NA		
TP53					LS	CB.HS	9 of 27	6 of 19	1.0000		
KIT					LS	CB.HS	1 of 27	2 of 19	0.5607		
PTEN	P2: PI3K-AKT-mTOR pathway	Activation of PI3K pathway through loss of PTEN or activation of other effectors reduces TILs		GOF	LS	CB.HS	2 of 27	0 of 19	0.5043		
PIK3CA					LS	CB.HS	4 of 27	3 of 19	1.0000		
AKT1					LS	CB.HS	2 of 27	0 of 19	0.5043		
AKT2					LS	CB.HS	0 of 27	2 of 19	0.1652		
AKT3					LS	CB.HS	NA	NA	NA		
EGFR					LS	CB.HS	2 of 27	3 of 19	0.6351		
DKK2	P2: WNT-b-catenin pathway	Increased WNT signaling decreases recruitment of		GOF	LS	CB.HS	NA	NA	NA		
BRAF					LS	CB.HS	2 of 27	1 of 19	1.0000		
CTNNB1					LS	CB.HS	NA	NA	NA		
IDO1	P2: IDO pathway	Increased IDO expression promotes activation of PI3K		GOF	LS	CB.HS	0 of 27	2 of 19	0.1652		
TGFB1	P2: TGFbeta	Increased TGFbeta expression promotes Treg activity while reduces cTL and NK cell activity		GOF	LS	CB.HS	NA	NA	NA		
B2M	P3: HLA variability and antigen presentation	LOH or loss of function in genes related to antigen presentation and processing impedes ability	immune evasion	LOF	LS	CB.HS	1 of 27	1 of 19	1.0000	11	0
HLA-A					LS	CB.HS	1 of 27	1 of 19	0.5607	4	2
HLA-B					LS	CB.HS	NA	NA	1.0000	7	2
HLA-C					LS	CB.HS	1 of 27	0 of 19	NA	3	5
TAP1					LS	CB.HS	NA	NA	NA		
TAP2					LS	CB.HS	NA	NA	NA		
IFNGR1	P3: JAK-STAT pathway	Lack of INFg effector function through defective JAK-STAT signaling		LOF	LS	CB.HS	NA	NA	NA		
JAK1					LS	CB.HS	1 of 27	1 of 19	1.0000		
JAK2					LS	CB.HS	NA	NA	NA		
JAK3					LS	CB.HS	0 of 27	2 of 19	0.1652		
APLNR					LS	CB.HS	0 of 27	2 of 19	0.1652		
SOCS1					LS	CB.HS	NA	NA	NA		
STAT1	P3: IFNg signaling	Lack of IFNg signaling mediates immune		LOF	LS	CB.HS	1 of 27	1 of 19	1.0000		
IFNGR2					LS	CB.HS	0 of 27	2 of 19	0.1652		
IRF1					LS	CB.HS	NA	NA	NA		
PTPN2					LS	CB.HS	NA	NA	NA		
PDCD1	P3: Immune checkpoint ligands	Loss of expression of checkpoint		LOF	LS	CB.HS	1 of 27	1 of 19	1.0000		
CD274					LS	CB.HS	NA	NA	NA		
ARID1A	P4: Chromatin remodeling	Loss of BAF/PBAF or EZH2-PRC2 complex induce IFNg	immune activation	LOF	LS	CB.HS	2 of 27	3 of 19	0.6351		
PBRM1					LS	CB.HS	2 of 27	1 of 19	1.0000		
SMARCA4					LS	CB.HS	0 of 27	2 of 19	0.1652		
EZH2					LS	CB.HS	0 of 27	2 of 19	0.1652		
YAP1	P5: Hippo signaling pathway	Immune suppression	Immune suppression	GOF	LS	CB.HS	NA	NA	NA		
MYCN					LS	CB.HS	0 of 27	2 of 19	0.1652		
MYC					LS	CB.HS	NA	NA	NA		
SERPINB3	P6: Prognostic in anti-CTLA4 treated melanoma	Frequently mutated in melanoma responders to	?	mutated	LS	CB.HS	1 of 27	3 of 19	0.2916		
SERPINB4					LS	CB.HS	2 of 27	1 of 19	1.0000		

Supplementary Table 2 - Flow cytometry antibody information and dilutions

Epitope	Fluor	Company	Cat. No.	Dilution
**T cell activation/exhaustion markers**				
TCRgd	**FITC**	eBioscience	11-9959-42	1:50
CD8	**PerCP**	BioLegend	301030	1:50
CD137	**PE**	eBioscience	12-1379-42	1:100
TIGIT	**PE-Cy7**	eBioscience	25-9500-42	1:50
CTLA-4 (surface)	**eFluor660**	eBioscience	50-1529-42	1:100
CD4	**Alexa700**	eBioscience	56-0048-42	1:100
CD19	**Alexa700**	eBioscience	56-0199-42	1:100
CD56	**APC-Cy7**	BioLegend	318332	1:100
PD-L1	**eF450**	eBioscience	48-5983-42	1:50
fixable viability	**e506**	eBioscience	65-0866-18	1:800
PD-1	**BV605**	BioLegend	329924	1:100
CD3	**BUV395**	BD	563546	1:25

SPONSOR: Princess Margaret Cancer Centre, Tumor Immunotherapy Program, Toronto, Ontario, Canada

TITLE: **IN**vestigator-initiated Phase II **S**tudy of **P**embrolizumab **I**mmunological **R**esponse **E**valuation **(INSPIRE-001)**

PRINCIPAL INVESTIGATOR : Lillian Siu, MD

SCIENTIFIC CO-PRINCIPAL INVESTIGATOR : Pamela Ohashi, PhD

TRANSLATIONAL CO-PRINCIPAL INVESTIGATOR : Marcus Butler, MD

OTHER SCIENTIFIC LEADS: Philippe Bedard, MD; David Cescon, MD; Eleftherios Diamandis, PhD; Scott Bratman, MD, PhD; David Brooks, PhD; Aaron Hansen, MBBS; Tracy McGaha, PhD; Linh Nguyen, PhD; Amit Oza, MD; Trevor Pugh, PhD, FACMG; Albiruni Razak, MB; Anna Spreafico, MD; Ming Tsao, MD

PROTOCOL VERSION: June 01, 2018

1.0 TRIAL SUMMARY

Abbreviated Title	INSPIRE-001
Trial Phase	II
Clinical Indication	Squamous cell cancer of head and neck (SCCHN)<br>Triple negative breast cancer (TNBC)<br>Ovarian (epithelial ovarian cancer type II)<br>Malignant melanoma (MM)<br>Mixed advanced solid tumors
Trial Type	Investigator-initiated interventional study
Type of control	No treatment control
Route of administration	Intravenous
Trial Blinding	Unblinded open-label
Treatment Groups	Pembrolizumab 200 mg IV Q3W
Number of trial subjects	Approximately 100 evaluable subjects will be enrolled
Estimated enrollment period	Estimated enrollment period is about 18 months
Estimated duration of trial	The sponsor estimates that the trial will require approximately 24 months from the time the first subject signs the informed consent until the last subject's last visit
Duration of Participation	Each subject will participate in the trial from the time the subject signs the Informed Consent Form (ICF) through the final protocol-specified contact. After a screening phase of 28 days, eligible subjects will receive treatment on Day 1 of each 3-week dosing cycle. Treatment with pembrolizumab will continue until documented confirmed disease progression, unacceptable adverse event(s), intercurrent illness that prevents further administration of treatment, investigator's decision to withdraw the subject, subject withdraws consent, pregnancy of the subject, noncompliance with trial treatment or procedure requirements; subject receives 35 administrations of study medication, or administrative reasons. Subjects who attain a complete response may consider stopping trial treatment if they meet criteria for holding therapy. Subjects who stop trial treatment after receiving 35 administrations of study medication for reasons other than disease progression or intolerability or who attain a complete response and stop trial treatment may be eligible for up to 17 cycles (approximately 1 year) of retreatment after experiencing disease progression. The decision to retreat will be at the discretion of the investigator only if they meet the criteria for retreatment and the trial is ongoing. After the end of treatment, each subject will be followed for 30 days for adverse event monitoring (serious adverse events will be collected for 90 days after the end of treatment). Subjects who discontinue for reasons other than disease progression will have post-treatment follow-up for disease status until disease progression, initiating a non-study cancer treatment, withdrawing consent, or becoming lost to follow-up. All subjects will be followed by telephone for overall survival until death, withdrawal of consent, or the end of the study.

2.0 TRIAL DESIGN

2.1 Trial Design

This is a single-centre, nonrandomized, multi-cohort trial of pembrolizumab in subjects with advanced solid tumors. Subjects will be enrolled into one of the following 5 solid tumor cohorts:

A Squamous Cell Cancer of Head and Neck (SCCHN)
B Triple Negative Breast Cancer (TNBC)
C Epithelial Ovarian Cancer (EOC) Type II
D Malignant Melanoma (MM)
E Advanced Solid Tumors

Approximately 100 evaluable subjects will be enrolled in this trial to examine the safety and efficacy of a 200mg dose of pembrolizumab administered every 3 weeks. Subjects will be considered evaluable if they have tumor sample available from the screening and on-treatment biopsy time-points of acceptable quality and quantity for analysis, as assessed by study site correlatives team (an estimate of 40 additional patients are required to yield 100 evaluable patients by this definition). Subjects will be evaluated every 9 weeks (63 days ±7 days) with radiographic imaging to assess response to treatment. After 9 months, radiography imaging will be evaluated every 12 weeks (84 days ±7 days). RECIST 1.1 will be used as the primary efficacy endpoint of response rate. RECIST 1.1 will be adapted due to the tumor response patterns seen with pembrolizumab treatment (e.g., tumor flare), and this adapted RECIST will be used by the sites for treatment decisions. Adverse events will be monitored throughout the trial and graded in severity according to the guidelines outlined in the NCI Common Terminology Criteria for Adverse Events (CTCAE) version 4.03. Treatment with pembrolizumab will continue until documented disease progression, unacceptable adverse event(s), intercurrent illness that prevents further administration of treatment, investigator's decision to withdraw the subject, subject withdraws consent, pregnancy of the subject, noncompliance with trial treatment or procedure requirements, completion of 35 treatments (approximately 2 years) with pembrolizumab, or administrative reasons. Subjects who attain an investigator-determined confirmed complete response (CR) may consider stopping trial treatment after receiving at least 8 cycles of treatment and at least 2 cycles treatment beyond the date when the initial CR was declared. In addition, subjects who discontinue after completion of 35 treatments (approximately 2 years) for reasons other than disease progression or intolerability may be eligible for up to 17 cycles (approximately 1 year) of retreatment if they subsequently experience radiographic disease progression. The decision to retreat will be at the discretion of the investigator only if no cancer treatment was administered since the last dose of pembrolizumab, the subject still meets the safety parameters listed in the Inclusion/Exclusion criteria and the trial remains open. After the end of treatment, each subject will be followed for 30 days for adverse event monitoring (serious adverse events will be collected for 90 days after the end of treatment). Subjects who discontinue treatment for reasons other than disease progression will have post-treatment follow-up of disease status until disease progression, initiating a non-study cancer treatment, withdrawing consent, or

becoming lost to follow-up. All subjects will be followed by telephone contact for overall survival until death, withdrawal of consent or the end of the study, whichever comes first.

The primary objective of this trial is to evaluate the changes in genomic and immune landscapes in patients with advanced various solid tumors during pembrolizumab treatment, and to perform a thorough immunological response evaluation.

Participation in this trial will be dependent upon supplying tissue from a newly obtained biopsy of a tumor lesion not previously irradiated (tumors progressing in a prior site of radiation are allowed, other exceptions may be considered after consultation with study principal investigators).

This study will be conducted in conformance with Good Clinical Practices.

Specific procedures to be performed during the trial, as well as their prescribed times and associated visit windows, are outlined in the Trial Flow Chart - Section 6.0. Details of each procedure are provided in Section 7.0 – Trial Procedures.

2.2 Trial Diagram

Figure 1: INSPIRE trial schema.

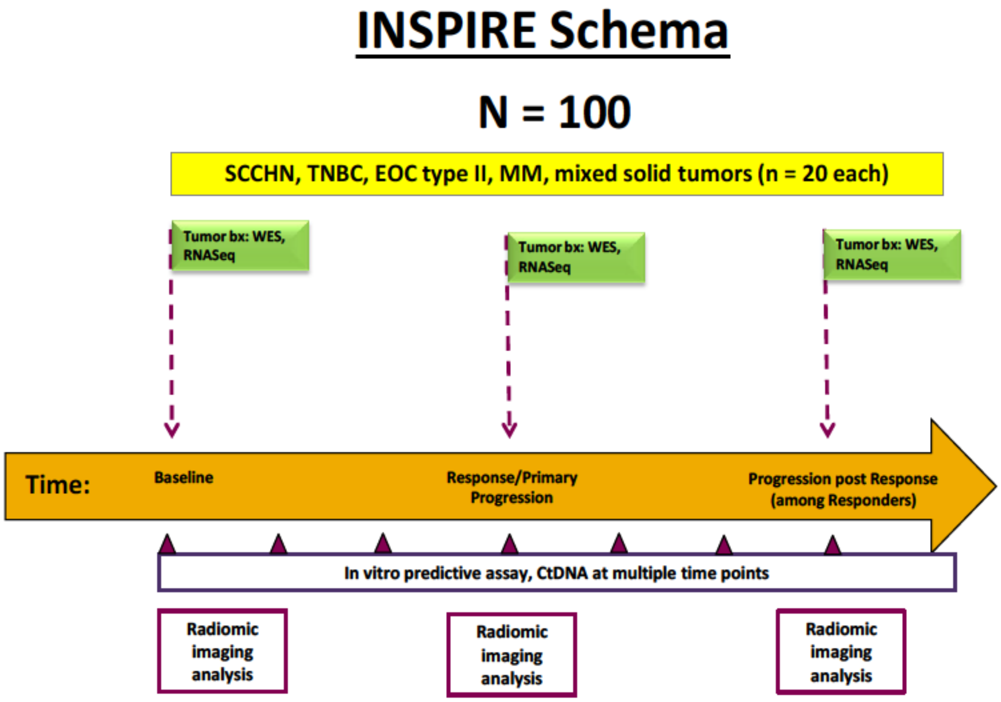

3.0 OBJECTIVE(S) & HYPOTHESIS(ES)

3.1 Primary Objective(s) & Hypothesis(es)

(1) **Objective:** To evaluate the changes in genomic and immune landscapes in patients with various advanced solid tumors during pembrolizumab treatment, and to perform a thorough immunological response evaluation

Hypothesis: In patients with advanced solid tumors who are treated with pembrolizumab, clonal dynamics of driver clones and subclones, as well as distinct immunological responses, can be observed with changes that correlate with clinical response

3.2 Secondary Objective(s) & Hypothesis(es)

(1) **Objective**: To evaluate the antitumor activity of single agent pembrolizumab in patients with various advanced solid tumors

Hypothesis: The overall response rates of pembrolizumab based on RECIST 1.1 in various advanced solid tumors are similar to those in completed or ongoing efficacy studies

(2) **Objective**: To assess the feasibility of using circulating tumor DNA analysis as a monitoring tool to inform clonal selection of cancer cells and its potential as an indicator of tumor burden in patients with advanced solid tumors being treated with pembrolizumab

Hypothesis: Circulating tumor DNA analysis can be used as a monitoring tool to inform clonal selection of cancer cells and tumor burden in patients with advanced solid tumors being treated with pembrolizumab

(3) **Objective**: To explore the potential value of radiomic imaging analysis as a predictive and pharmacodynamic biomarker to pembrolizumab

Hypothesis: Radiomic imaging analysis has the potential to be a predictive and pharmacodynamic biomarker to pembrolizumab

(4) **Objective**: To investigate whether any correlation or relationship exists between tumor genomic profiles and radiomic imaging signatures

Hypothesis: There are characteristic radiomic imaging signatures that are associated with genomic profiles of tumors and the immunological infiltrates induced by pembrolizumab treatment

(5) **Objective**: To explore changes in immune cell subsets in the peripheral circulation and tumor microenvironment during treatment with pembrolizumab

Hypothesis: Changes in the phenotype of immune cell subsets in peripheral circulation and in tumors from patients treated with pembrolizumab will be observed during response and subsequent progression

(6) **Objective**: To evaluate the positive and negative predictive values of an in vitro predictive assay in distinguishing responders from non-responders among patients receiving pembrolizumab

Hypothesis: The positive and negative predictive values of an in vitro predictive assay in distinguishing responders from non-responders among patients receiving pembrolizumab are over 90%

(7) **Objective**: To evaluate the distribution on tumor penetration of pembrolizumab using a mass spectrometry based monitoring assay

Hypothesis: The selected reaction monitoring assay can be correlated with therapeutic response to pembrolizumab

(8) **Objective:** To examine RNA expression from baseline biopsies, for immune inhibitory genes that may predict non-responders

Hypothesis: Immune inhibitory pathways are augmented in patients that do not respond to pembrolizumab

(9) **Objective:** To compare RNA expression profiles between baseline biopsies and responding patients and identify biomarkers or a signature that correlates with patient response

Hypothesis: Changes in the inflammation status in the tumor may provide biomarker(s) that correlate with patient response

4.0 BACKGROUND & RATIONALE

4.1 Background

Refer to the Investigator's Brochure (IB)/approved labeling for detailed background information on pembrolizumab (MK-3475).

4.1.1 Pharmaceutical and Therapeutic Background

The importance of intact immune surveillance in controlling outgrowth of neoplastic transformation has been clearly demonstrated in animal models[1]. Accumulating evidence shows a correlation between tumor-infiltrating lymphocytes (TILs) in cancer tissue and

favorable prognosis in various malignancies. In particular, the presence of CD8+ T-cells and the ratio of CD8+ effector T-cells / FoxP3+ regulatory T-cells to correlates with improved prognosis and long-term survival in many solid tumors[2].

T cell activation is regulated by a variety of activating and inhibitory receptor/ligand interactions. One of the well-studied inhibitory molecules is PD-1, which is expressed on the cell surface of activated T-cells as well as 'exhausted' T cells[3,4]. PD-1 (encoded by the gene Pdcd1) is an immunoglobulin (Ig) superfamily member related to CD28 and CTLA-4, which negatively regulates antigen receptor signaling upon engagement of its ligands (PD-L1 and/or PD-L2). PD-1 and family members are type I transmembrane glycoproteins containing an Ig Variable-type (V-type) domain responsible for ligand binding and a cytoplasmic tail which is responsible for the binding of signaling molecules. The cytoplasmic tail of PD-1 contains 2 tyrosine-based signaling motifs, an immunoreceptor tyrosine-based inhibition motif (ITIM) and an immunoreceptor tyrosine-based switch motif (ITSM). PD-1 was shown to be expressed on activated lymphocytes including peripheral CD4+ and CD8+ T-cells, B-cells, T regs and Natural Killer cells. The ligands for PD-1 (PD-L1 and PD-L2) are constitutively expressed or can be induced in a variety of cell types, including non-hematopoietic tissues as well as in various tumors. Both ligands are type I transmembrane receptors containing both IgV- and IgC-like domains in the extracellular region and contain short cytoplasmic regions with no known signaling motifs. Binding of either PD-1 ligand to PD-1 inhibits T-cell activation triggered through the T-cell receptor. PD-L1 is expressed at low levels on various non-hematopoietic tissues, most notably on vascular endothelium, whereas PD-L2 protein is only detectably expressed on antigen-presenting cells found in lymphoid tissue or chronic inflammatory environments. PD-L2 is thought to control immune T-cell activation in lymphoid organs, whereas PD-L1 serves to dampen unwarranted T-cell function in peripheral tissues. Although healthy organs express little (if any) PD-L1, a variety of cancers were demonstrated to express this T-cell inhibitor. Recent studies suggest that PD-L1 is upregulated only when tumor cells are in close proximity with T cells in the tumor microenvironment[5-7]. This suggests that the PD-1/PD-L1 pathway may play a critical role in tumor immune evasion and should be considered as an attractive target for therapeutic intervention.

Pembrolizumab is a potent and highly selective humanized monoclonal antibody (mAb) of the IgG4/kappa isotype designed to directly block the interaction between PD-1 and its ligands, PD-L1 and PD-L2. Keytruda™ (pembrolizumab; MK-3475) has recently been approved in the United Stated for the treatment of patients with unresectable or metastatic melanoma and disease progression following ipilumumab and, if BRAF V600 mutation positive, a BRAF inhibitor.

4.1.2 Preclinical and Clinical Trial Data

Refer to the Investigator's Brochure for Preclinical and Clinical data.

4.2 Rationale

4.2.1 Rationale for the Trial and Selected Subject Population

This study is designed as a prospective research study in which patients will have tumor and blood samples collected at serial time points to investigate the changes in genomic and immune landscapes of tumors under the selection treatment pressure of the immune checkpoint inhibitor (ICI), pembrolizumab. Patients will have image-guided fresh tumor core needle biopsy at a maximum of 3 time points: 1) prior to commencement of pembrolizumab, 2) on-treatment during pembrolizumab, and 3) when radiological disease progression on therapy is confirmed among patients who had initial response. Surgical resection of metastasis for time point (2) or (3) may be considered in some patients in place of biopsy, if appropriate. Patients who have disease response to pembrolizumab could have up to 3 fresh tumor biopsies (all 3 time points) whereas those who do not respond will only have two biopsies (1st and 2nd time points). During their participation on INSPIRE, patients who have a surgery, biopsy or other procedure as part of their care outside of this trial's specified investigations may have a sample of tissue, blood or fluid collected for biomarker research in this study if it is feasible and safe, as judged by staff surgeon or radiologist performing the procedure. Patients who have pleural drained by therapeutic thoracentesis or ascites fluid drained by paracentesis may also have an aliquot of these samples collected for biomarker research, if deemed feasible by the study investigator. Blood samples for ctDNA and immune analysis will be collected at baseline, and thereafter ideally coinciding with radiological tumor assessments whenever possible until radiological disease progression is confirmed. Normal genomic DNA will be extracted from whole blood collected at baseline to study normal variants. Imaging parameters for radiomic imaging analysis will be derived from patients' routine CT scans.

The tumor types were selected in INSPIRE such that a mixture of solid tumors are evaluated for the various hypotheses stated. Pembrolizumab has already shown antitumor activity in most of the tumor types in previously presented/published studies (Table 1). The main goal of INSPIRE is not specifically to assess the efficacy of pembrolizumab in these tumor types, but rather, to perform comprehensive molecular, genomic, immunologic, proteomic and genomic profiling to understand the dynamic changes in tumors and their microenvironment following treatment with pembrolizumab, and to identify predictors of sensitivity or resistance (primary and acquired) to this agent.

Table 1 Antitumor Activity of Pembrolizumab to Selected Tumor Types Being Studied in INSPIRE

Tumor type	Sample size	PD-L1 status	Objective response	Stable disease or PFS/OS
Recurrent or metastatic SCCHN[8]	117	Both + and -	Overall = 24.8%<br>HPV+ = 20.6%<br>HPV- = 27.2%	Overall = 24.8%<br>HPV+ = 26.5%<br>HPV- = 23.5%
Recurrent or metastatic TNBC[9]	32	+	PR = 16.1%	SD = 9.7%
Advanced EOC[10]	26	+	Overall = 11.5%	SD = 23.1%

			CR = 3.8%<br>PR = 7.7%	
Advanced MM[11]	834	Both + and -	Overall for pembrolizumab<br>Q2W = 33.7%<br>Q3W = 32.9%	Median PFS for pembrolizumab<br>Q2W = 5.5 mo<br>Q3W = 4.1 mo<br><br>1-year survival for pembrolizumab<br>Q2W = 74.1%<br>Q3W = 68.4%

4.2.2 Rationale for Dose Selection/Regimen/Modification

An open-label Phase I trial (Protocol 001) is being conducted to evaluate the safety and clinical activity of single agent pembrolizumab. The dose escalation portion of this trial evaluated three dose levels, 1 mg/kg, 3 mg/kg, and 10 mg/kg, administered every 2 weeks (Q2W) in subjects with advanced solid tumors. All three dose levels were well tolerated and no dose-limiting toxicities were observed. This first in human study of pembrolizumab showed evidence of target engagement and objective evidence of tumor size reduction at all dose levels (1 mg/kg, 3 mg/kg and 10 mg/kg Q2W). No maximum tolerated dose (MTD) has been identified to date. Recent data from other clinical studies within the pembrolizumab program has shown that a lower dose of pembrolizumab and a less frequent schedule may be sufficient for target engagement and clinical activity.

Pharmacokinetic (PK) data analysis of pembrolizumab administered Q2W and Q3W showed slow systemic clearance, limited volume of distribution, and a long half-life (refer to IB). Pharmacodynamic data (IL-2 release assay) suggested that peripheral target engagement is durable (>21 days). This early PK and pharmacodynamic data provides scientific rationale for testing a Q2W and Q3W dosing schedule.

A population pharmacokinetic analysis has been performed using serum concentration time data from 476 patients. Within the resulting population PK model, clearance and volume parameters of pembrolizumab were found to be dependent on body weight. The relationship between clearance and body weight, with an allometric exponent of 0.59, is within the range observed for other antibodies and would support both body weight normalized dosing or a fixed dose across all body weights. Pembrolizumab has been found to have a wide therapeutic range based on the melanoma indication. The differences in exposure for a 200 mg fixed dose regimen relative to a 2 mg/kg Q3W body weight based regimen are anticipated to remain well within the established exposure margins of 0.5 – 5.0 for pembrolizumab in the melanoma indication. The exposure margins are based on the notion of similar efficacy and safety in melanoma at 10 mg/kg Q3W vs. the proposed dose regimen of 2 mg/kg Q3W (i.e. 5-fold higher dose and exposure). The population PK evaluation revealed that there was no significant impact of tumor burden on exposure. In addition, exposure was similar between the NSCLC

and melanoma indications. Therefore, there are no anticipated changes in exposure between different indication settings.
The choice of the 200 mg Q3W as an appropriate dose for the switch to fixed dosing is based on simulations performed using the population PK model of pembrolizumab showing that the fixed dose of 200 mg every 3 weeks will provide exposures that 1) are optimally consistent with those obtained with the 2 mg/kg dose every 3 weeks, 2) will maintain individual patient exposures in the exposure range established in melanoma as associated with maximal efficacy response and 3) will maintain individual patients exposure in the exposure range established in melanoma that are well tolerated and safe.

A fixed dose regimen will simplify the dosing regimen to be more convenient for physicians and to reduce potential for dosing errors. A fixed dosing scheme will also reduce complexity in the logistical chain at treatment facilities and reduce wastage. Therefore, pembrolizumab at 200 mg IV Q3W has been selected for the current INSPIRE protocol.

4.2.3 Rationale for Endpoints

4.2.3.1 Efficacy Endpoints

The efficacy objective of this trial is to evaluate the antitumor activity of pembrolizumab in subjects with a variety of malignancies. Response rates per RECIST 1.1 will be evaluated and will serve as the efficacy endpoint. As the primary objective of this study is to perform correlative studies, the efficacy objective is a secondary objective of INSPIRE. The response rates observed are expected to be in line with those reported in ongoing or completed clinical trials of pembrolizumab in the tumor types under evaluation.

4.2.3.2 Biomarker Research - Tumor-Based

4.2.3.2.1 <u>DNA/RNA Sequencing</u>

To discover genomic correlates with immunotherapy, we will use an integrated DNA/RNA analysis approach to define clonal and subclonal read-outs of somatic mutation burden, infer the presence of mutant neoantigenic peptides, and nomination of abnormal tissue-specific expression of wild-type transcripts that may mediate an immune response. The choice of integrated exome/RNAseq analysis strikes a balance between cost-effective profiling of all available tissues, maximizing the survey of cancer genome variant types, and enabling complementary analysis and functional read-out of candidate variants. Similar data types are available from thousands of unselected tumors profiled through the International Cancer Genome Consortium (ICGC), The Cancer Genome Atlas (TCGA), and Therapeutically Applicable Research To Generate Effective Treatment (TARGET), and provide context for interpretation of individual tumors. There are also large collections of normal tissues profiled through the Genotype-Tissue Expression (GTEx) project using these same techniques to enable comparison within and across tissue sites. The Pugh Lab has gained controlled access to all of these data sets and has local copies of these data that have all been reanalyzed using a single computational analysis pipeline.

Exome + UTR analysis

For exome analysis, we will initially use the Agilent SureSelectXT Human All Exon V5+UTRs kit that employs hybrid selection to isolate 75 Mb of DNA representing coding exons and untranslated regions (UTRs) of genes annotated by five databases. Tumour and matched blood DNA will be sequenced (100 bp paired-end reads) on Illumina HiSeq 2000 instruments at the Princess Margaret Genomics Centre (coverage 250X tumours, 50X normal). Reads will be aligned using bwa[12] and processed using the Genome Analysis Toolkit Best Practices workflow. Somatic mutations will be called using muTect[13] and VarScan2[14] then annotated by Oncotator[15]. Deep exome sequence will enable inference of allele-specific copy number profiles, tumor ploidy and purity[16], and subclonal structure[17], as well as precise measurement of background somatic mutation rate and sequence context indicative of external exposures. Should costs of sequencing continue to fall, we will consider a move to whole genome sequencing, as many of our informatics methods are directly applicable to these data as well.

Overall mutation burden will be corrected for coverage of all regions across the exome and correlated with treatment outcome. In collaboration with the Ohashi lab, we will employ several published antigen prediction algorithms to infer specific antigens expression within each clonal population found in each tumor tissue.

RNA-seq analysis

For RNA-seq, we will generate sequencing libraries using approaches such as the Illumina TruSeq Stranded Total RNA with Ribo-Zero Gold kit, an approach that uses randomly primed cDNA synthesis to enable unbiased amplification along the length of RNA transcripts[18]. My group has independently confirmed the applicability of these methods to highly degraded RNAs. We will generate 80 million 100 bp paired-end reads per library, thereby enabling mutation confirmation in genes of sufficient expression level[19], measurement of gene expression levels and novel exon-usage patterns[20,21] , and detection of expressed fusion transcripts[22]. We have experience with RNA-seq analysis frameworks[23,24] and have local access to >3,600 data sets derived from normal tissue from the GTEx[25].

RNA-seq also serves as a functional read-out of mutations, copy number alterations, and methylation patterns to further the interpretation of these data types. RNA-seq also serves as a discovery data set to enable detection of fusion transcripts, altered gene expression levels and isoform usage patterns that may not be directly predicted from exome analysis. Furthermore, integration of RNA-seq analysis with GTEx and TCGA data will enable confirmation of cancer type and tissue of origin, as well as detection of wild-type sequences expressed in unusual tumor- or tissue-specific context.

4.2.3.2.2 Immunoprofiling and Growth of Tumor Infiltrating Lymphocytes

Biomarker studies of pembrolizumab and other immune modulating agents are actively being investigated in the clinic to identify predictors of response or resistance to treatment. Additionally, data generated from these studies may provide the rationale for new combination approaches with emerging therapeutic agents. Initial studies suggested that anti-tumor clinical

activity of anti-PD-1 therapies was associated with PD-L1 expression by the tumor. While a positive association has been confirmed with further studies, PD-L1 expression has not been shown to be an absolute biomarker of clinical response. Recent studies suggest that T cell–mediated immunity is enhanced by PD-1 pathway blockade and that clinical activity can be correlated with an induction of immune cell infiltration of the tumor and/or invasive margin following therapy[26]. Differences in mechanisms of action and resistance can be expected between tumors of different histologies, depending on presence of pre-existing immune recognition, mutational load, and presence of immune suppressive cells (i.e. regulatory T cells, tumor associated macrophages). Therefore, a comprehensive analysis of the tumor microenvironment for each cohort of this study will be performed. Tumor samples will be biopsied at baseline and after the initiation of therapy. Patients with clinical responses or prolonged stable disease (>4 months) who subsequently progress will also undergo tumor sampling.

Analysis of the tumor microenvironment will be performed using several methods. Core biopsies or, when possible, excisional biopsies will be performed. For this analysis tumor samples will be prepared for immunohistochemistry (formalin fixed, paraffin embedded), or for flow cytometry (processed fresh). The complexity of the tumor immune microenvironment will be evaluated by standard IHC and, when possible, with multiplexed IHC techniques with the purpose of assessing the spatial distribution of immune cell populations within the tumor microenvironment. Fresh samples will be analyzed by methods such as multiparameter flow cytometry, CyToF, or protein quantification assays to assess the tumor microenvironment.

When sample is limited, the priority will be for IHC staining for PD-L1 expression by tumor and inflammatory cells and the presence of CD8 T cells within the tumor, surrounding stroma, and the invasive margin. Additional exploratory analysis by IHC and/or flow cytometry or CyToF would include characterization of immune cell populations such as: lymphocytes (CD3, CD4, CD8, FOXP3), macrophages (CD68), B cells (CD20), NK cells (CD56), markers of activation and exhaustion (e.g. HLA-DR, ICOS, granzyme B, PD-1, CTLA-4, LAG-3, Tim-3, KLRG1, CD160, 2B4, BTLA), and immunosuppressive factors (IDO, arginase).

Additionally, tumor infiltrating lymphocytes (TIL) may be expanded in vitro from fresh samples by incubating with cytokine (IL-2) and/or with anti-CD3/CD28 activation for further characterization of phenotype and function. Core biopsies or excisional biopsy tumor material will undergo mechanical and enzymatic digestion and viable cells placed into culture. Incubation with IL-2 (6000 IU/ml) with or without stimulation with anti-CD3/CD28 or artificial antigen presenting cells (aAPC) expressing membrane bound OKT3 with CD80 and CD83 will be performed to expand TIL[27,28]. This will allow for sufficient numbers of lymphocytes to characterize antigen specificity by functional analysis (e.g. interferon-γ secretion). For example, the ability of expanded T cells to recognize neoantigens generated through mutations identified by DNA/RNA sequencing

If sufficient material is available, then tumor samples will be used to generate patient-derived xenografts (PDX) in mice. The PDX would provide additional quantities of tumor cells for assessing responses to cancer therapy, and would provide an in vivo model for assessing the function of in vitro –expanded autologous TILs.

4.2.3.2.3 PD-L1 Immunohistochemistry

Samples will be sent to QualTek for PD-L1 testing using DAKO 22C3.

In brief, following SOPs, harvested tissue is placed immediately into at least 15 volumes of 10% Neutral buffered formalin (4 gm. of Sodium dihydrogen phosphate, monohydrate and 6.5 gm. Disodium hydrogen phosphate, anhydrous in 1 litre of 3.7% formaldehyde). Tissues will be fixed for a maximum of 96 hours at room temperature and then prepared for processing into a paraffin blocks.

4.2.3.3 Biomarker Research - Blood-Based

4.2.3.3.1 Peripheral Blood Mononuclear Cell and Serum Analysis

An exploratory analysis of peripheral blood will be performed to phenotypically characterize cellular subsets such as effector lymphocyte, dendritic cell, regulatory T cell, and myeloid derived suppressor cell populations. Analysis will be performed using multiparameter flow cytometry or CyTOF. Analysis (as sample material allows) will include characterization of cell populations such as: CD3, CD4, CD8 (T cells), CD19 (B cells), CD14 (monocytes), CD56 (NK cells), CD11c/HLA-DR (DCs); differentiation/memory status: CD45RO, CD28, CD27, CCR7; characterization of CD4+ T helper cell subsets: CD161, CCR6, CXCR3, CCR4; CD4+ regulatory T cells: HELIOS, Foxp3, CD127 (IL7-Ralpha), CD25; Effector T cells: ICOS, TIA-1, perforin, granzyme B; and putative markers of T cell exhaustion such as: LAG-3, Tim-3, PD-1, CTLA-4, KLRG1, CD160, 2B4, BTLA. Functional characterization may be performed using in vitro assays.

Serum will also be collected with each cycle and stored for correlation with clinical activity and development of toxicity. Multiplex platforms such as the Luminex platform may be used to measure chemokines/cytokines involved in responses such as: Th1/Tc1 responses (e.g. IFN-γ, TNF, IL-2), Th2/Tc1 responses (e.g. IL-4, IL-5, IL-10), pro-inflammatory innate responses (e.g. IFN-α, IL-1β, IL-6), homeostatic lymphocyte expansion (e.g. IL-7, IL-15) and chemotaxis of immune cells (e.g. IP-10, MCP-1, MIP-1α, MIP-1β, RANTES). Proteins that may influence anti-tumor immunity or toxicities may also be analyzed by other standard approaches such as enzyme-linked immunosorbent assays (ELISAs) or protoarrays. Metabolites that modulate immunity may be analyzed by high-performance liquid chromatography (HPLC).

4.2.3.3.2 Predictive Biomarker Assay (in vitro Culture System)

Preliminary data suggests the presence of a differential expression pattern of various activation markers following in vitro stimulation of peripheral blood T cells in patients who respond to immune checkpoint blockade compared to non-responders. In this study, as sample material allows, peripheral blood mononuclear cells (PBMCs) taken at baseline will be used to perform an exploratory analysis of this in vitro assay for its potential predictive value. Cryopreserved PBMCs will be thawed and stimulated with anti-CD3 mAb. Flow cytometric analysis will be performed at various time points for markers such as CD4, CD8, HLA-DR, CD25, and 41BB. Expression patterns will be interrogated for associations with clinical responses.

4.2.3.3.3 Circulating Tumor DNA

Genetic determinants of treatment-resistance can be found within plasma-derived circulating tumour DNA (ctDNA). The emergence of drug-resistant clones is detectable by ctDNA analysis well before cancer progression is appreciated using conventional medical imaging[29,30]. Next generation sequencing of ctDNA has revealed tumour evolution and clonal selection for acquired resistance to anticancer therapies in patients with advanced cancers; however, such studies have not yet been performed in the context of ICIs such as pembrolizumab. At each collection time point on INSPIRE, ~30 mL of peripheral blood will be collected in EDTA tubes, and plasma will be separated from the cell pellet within 2 hours. Cell-free DNA will be purified from clarified plasma using the Circulating Nucleic Acid Kit (Qiagen). Multiplexed Illumina libraries will be constructed and subjected to custom hybrid capture and deep sequencing[31].

The absolute concentration of ctDNA within peripheral blood plasma will be calculated from the aggregated tumor-specific reads and normalized to the total concentration of cell-free DNA. To examine changes within ctDNA in response to treatment with ICIs, sets of somatic mutations that are identified within distinct tumor sub-clones will be aggregated separately for comparison of relative abundance. Mutations will also be aggregated into distinct sets based on whether they are predicted to result in (1) defective immune surveillance and tolerance (based on Gene Ontology and ImmPort tools, https://immport.niaid.nih.gov/), or (2) the creation of neoantigens that bind to patient-specific MHC class I and activate T cells (based on the method of Snyder et al[32]).

4.2.3.3.4 Proteomic Analysis

Purified injectable drug (to be obtained from Merck) will be denatured, reduced and trypsin-digested, then subjected to detailed proteomic analysis with a Q-Exactive mass spectrometer, to characterize by the de-novo sequencing all tryptic peptides that are released from the parent drug. Bioinformatic analysis will then be conducted to identify 1 or more unique peptides of this antibody that can be found only in the drug sequence, but not in natural human immunoglobulins. Identification of such unique peptides will allow for development of a highly sensitive and specific selected reaction monitoring (SRM) assay with a triple quadruple mass spectrometer, to directly quantify pemimmurolizumab in patient blood. This method will allow for the first time to assess the levels of the drug after each treatment cycle, and examine if there are significant pharmacodynamic differences between the various groups of patients, and if these differences are associated with the effectiveness of the drug to elicit a therapeutic response.

We will develop monoclonal antibodies which target specifically unique peptides of the drug (i.e. peptides that are not found in natural immunoglobulins). We will use these monoclonal antibodies to develop an immunocapture purification method for the drug, from the blood of treated patients. The objective of this step is to develop a robust method to isolate the drug after it has been exposed to the patient circulation, and examine for the first time if the drug binds to non-cognate targets (e.g. molecules that are not related to programmed death receptor 1). If this is the case, the ramifications could be highly important since it is possible (but not

as yet examined), that most of the active drug in such patients is inactivated by interaction with non-target binding, before it reaches its intended target. We anticipate that we will be able to delineate which off-target molecules are associated with the therapeutic antibodies by using mass spectrometry. We will isolate the drug circulating in blood, then elute possible non-cognate binding antigens and then characterize these molecules by using mass spectrometry. It is possible that the degree of interaction of these therapeutic antibodies with non-cognate ligands correlates closely with the therapeutic effectiveness of the drug.

Our mass spectrometry assay, developed as described above, could be instrumental in assessing the amount of antibody that reaches the tumor. This could be an important determinant of the therapeutic response of the drug, and could be assessed with our SRM assay (to be developed). The minimum number of tubes required will be 3 (baseline, during treatment, after treatment). One 7ml red-top tube should be collected specifically for this project and the tube will be centrifuged between1-2 hours of collection, to separate serum. Serum will then be aliquoted and stored until use.

4.2.3.4 Radiomic Research

Computerized tomography (CT) scans are universally used to assess patient responses to treatment. It contains information about the tumor, such as shape and texture, which is mineable using automatically extracted data characterization algorithms. Radiomics is an emerging field that focuses on the quantitative analysis of available medical images, capturing distinct tumor phenotypic differences, which may have prognostic and predictive significance[33,34].

In a study by Aerts et al, radiomic analysis of independent data sets from 1,019 head and neck and lung cancer patients revealed a prognostic radiomic signature that is associated with intratumoral heterogeneity. This non-invasive, low-cost technique provides a significant opportunity for prognostic stratification of patients that may help guide treatment choice[35]. Quantitative analysis of available CT images of head and neck cancer patients on ICIs may reveal a pattern of radiomics signature that may be used to predict clinical response.

For INSPIRE, a bank of texture features will be computed for each segmented image based on the image voxels lying inside the tumor Volume of Interest (VOI). A team of computational scientist(s), radiologist(s) and medical oncologist(s) will determine the panel of radiomic algorithms to be used for analysis.

Confidential

5.0 METHODOLOGY

5.1 Entry Criteria

5.1.1 Diagnosis/Condition for Entry into the Trial

5.1.2 Subject Inclusion Criteria

In order to be eligible for participation in this trial, the subject must:

1. Be willing and able to provide written informed consent/assent for the trial.

2. Be ≥ 18 years of age on day of signing informed consent.

3. Have histologically or cytologically-documented, locally-advanced, or metastatic solid malignancy that is incurable and has either (a) failed prior standard therapy, (b) for which no standard therapy exists, or (c) standard therapy is not considered appropriate by the patient and treating physician. There is no limit to the number of prior treatment regimens.

   Note: For malignant melanoma patients, where pembrolizumab is considered acceptable as a standard therapy, are eligible for the trial even if they have not failed a prior standard therapy.

4. Have one of the following advanced (unresectable and/or metastatic) solid tumor indications:

   A Squamous Cell Cancer of Head and Neck (SCCHN)[a]
   B Triple Negative Breast Cancer (TNBC)[b]
   C Epithelial Ovarian Cancer (EOC) Type II[c]
   D Malignant Melanoma (MM)[d]
   E Advanced Solid Tumors

   [a] Note: Patients with primary tumors originating from oropharynx, larynx, oral cavity and hypopharynx are eligible. Other primary sites, such as unknown primary, paranasal sinus, nasopharynx are not eligible.

   [b] Note: Definition of TNBC is based onAmerican Society of Clinical Oncology-College of American Pathologists (ASCO-CAP) guidelines[36].

   [c] Note: High grade serous ovarian, primary peritoneal, fallopian tube carcinomas are eligible. Type I EOCs are not eligible.

   [d] Note: Cutaneous melanomas and non-cutaneous melanomas (uveal, mucosal etc.) are eligible.

5. Have measurable disease based on RECIST 1.1.

6. Be willing to provide tissue from a newly obtained core or excisional biopsy of a tumor lesion. *Newly-obtained is defined as a specimen obtained up to 6 weeks (42 days) prior to initiation of treatment on Day 1. Subjects for whom newly-obtained samples cannot be provided (e.g. inaccessible or subject safety concern) will not be eligible for this study.*

7. Have a performance status of 0 or 1 on the ECOG Performance Scale.

8. Demonstrate adequate organ function as defined in Table 2, all screening labs should be performed within 10 days of treatment initiation.

Table 2 Adequate Organ Function Laboratory Values

System	Laboratory Value
**Hematological**	
Absolute neutrophil count (ANC)	≥1,500 /mcL
Platelets	≥100,000 / mcL
Hemoglobin	≥9 g/dL without transfusion or EPO dependency (within 7 days of assessment)
**Renal**	
Serum creatinine **OR**<br>Measured or calculated[a] creatinine clearance<br>(GFR can also be used in place of creatinine or CrCl)	≤1.5 X upper limit of normal (ULN) **OR**<br><br>≥60 mL/min for subject with creatinine levels > 1.5 X institutional ULN
**Hepatic**	
Serum total bilirubin	≤ 1.5 X ULN **OR**
	Direct bilirubin ≤ ULN for subjects with total bilirubin levels > 1.5 ULN
AST (SGOT) and ALT (SGPT)	≤ 2.5 X ULN **OR**<br>≤ 5 X ULN for subjects with liver metastases
Albumin	≥25 g/L
**Coagulation**	
International Normalized Ratio (INR) or Prothrombin Time (PT)	≤1.5 X ULN unless subject is receiving anticoagulant therapy as long as PT or PTT is within therapeutic range of intended use of anticoagulants
Activated Partial Thromboplastin Time (aPTT)	≤1.5 X ULN unless subject is receiving anticoagulant therapy as long as PT or PTT is within therapeutic range of intended use of anticoagulants
[a]Creatinine clearance should be calculated per institutional standard.	

9. Female subject of childbearing potential should have a negative urine or serum pregnancy within 72 hours prior to receiving the first dose of study medication. If the urine test is positive or cannot be confirmed as negative, a serum pregnancy test will be required.

10. Female subjects of childbearing potential should be willing to use 2 methods of birth control or be surgically sterile, or abstain from heterosexual activity for the course of the study through 120 days after the last dose of study medication (Reference Section

5.7.2). Subjects of childbearing potential are those who have not been surgically sterilized or have not been free from menses for > 1 year.

11. Male subjects should agree to use an adequate method of contraception starting with the first dose of study therapy through 120 days after the last dose of study therapy.

5.1.3 Subject Exclusion Criteria

The subject must be excluded from participating in the trial if the subject:

1. Is currently participating and receiving study therapy or has participated in a study of an investigational agent and received study therapy or used an investigational device within 4 weeks of the first dose of treatment.

2. Has a diagnosis of immunodeficiency or is receiving systemic steroid therapy or any other form of immunosuppressive therapy within 7 days prior to the first dose of trial treatment.

3. Has a known history of active TB (Bacillus Tuberculosis).

4. Hypersensitivity to pembrolizumab or any of its excipients.

5. Has had a prior anti-cancer monoclonal antibody (mAb) within 4 weeks prior to study Day 1 or who has not recovered (i.e., ≤ Grade 1 or at baseline) from adverse events due to agents administered more than 4 weeks earlier.

6. Has had prior chemotherapy, targeted small molecule therapy, or radiation therapy within 2 weeks prior to study Day 1 or who has not recovered (i.e., ≤ Grade 1 or at baseline) from adverse events due to a previously administered agent.

    - Note: Subjects with ≤ Grade 2 neuropathy or alopecia are an exception to this criterion and may qualify for the study.

    - Note: If subject received major surgery, they must have recovered adequately from the toxicity and/or complications from the intervention prior to starting therapy.

7. Has a known additional malignancy that is progressing or requires active treatment. Exceptions include basal cell carcinoma of the skin or squamous cell carcinoma of the skin that has undergone potentially curative therapy or in situ cervical cancer.

8. Has known active central nervous system (CNS) metastases and/or carcinomatous meningitis. Subjects with previously treated brain metastases may participate provided they are stable (without evidence of progression by imaging for at least four weeks prior to the first dose of trial treatment and any neurologic symptoms have returned to baseline), have no evidence of new or enlarging brain metastases, and are not using

steroids for at least 7 days prior to trial treatment. This exception does not include carcinomatous meningitis which is excluded regardless of clinical stability.

9. Has active autoimmune disease that has required systemic treatment in the past 2 years (i.e. with use of disease modifying agents, corticosteroids or immunosuppressive drugs). Replacement therapy (e.g. thyroxine, insulin, or physiologic corticosteroid replacement therapy for adrenal or pituitary insufficiency, etc.) is not considered a form of systemic treatment.

10. Has known history of, or any evidence of active, non-infectious pneumonitis.

11. Has evidence of interstitial lung disease.

12. Has an active infection requiring systemic therapy.

13. Has a history or current evidence of any condition, therapy, or laboratory abnormality that might confound the results of the trial, interfere with the subject's participation for the full duration of the trial, or is not in the best interest of the subject to participate, in the opinion of the treating investigator.

14. Has known psychiatric or substance abuse disorders that would interfere with cooperation with the requirements of the trial.

15. Is pregnant or breastfeeding, or expecting to conceive or father children within the projected duration of the trial, starting with the pre-screening or screening visit through 120 days after the last dose of trial treatment.

16. Has received prior therapy with an anti-PD-1, anti-PD-L1, or anti-PD-L2 agent. Prior anti-CTLA4 agents are allowed. Prior therapy with T-cell co-stimulatory agents (e.g. anti-CD137 antibody, anti-OX40 antibody) are allowed.

17. Has a known history of Human Immunodeficiency Virus (HIV) (HIV 1/2 antibodies).

18. Has known active Hepatitis B (e.g., HBsAg reactive) or Hepatitis C (e.g., HCV RNA [qualitative] is detected).

19. Has received a live vaccine within 30 days of planned start of study therapy.

    Note: Seasonal influenza vaccines for injection are generally inactivated flu vaccines and are allowed; however intranasal influenza vaccines (e.g., Flu-Mist®) are live attenuated vaccines, and are not allowed.

5.2 Trial Treatments

The treatment to be used in this trial is outlined below in Table 3.

Table 3 Trial Treatment*

Drug	Dose/Potency	Dose Frequency	Route of Administration	Regimen/Treatment Period	Use
Pembrolizumab	200 mg	Q3W	IV infusion	Day 1 of each 3 week cycle	Experimental

*Trial treatment should begin within 3 days (working days) of study registration.

5.2.1 Dose Selection/Modification

5.2.1.1 Dose Selection

The rationale for selection of the dose to be used in this trial is provided in Section 4.0 – Background and Rationale.

Details on preparation and administration of pembrolizumab are provided in the Pharmacy Manual.

5.2.1.2 Dose Modification

Adverse events (both non-serious and serious) associated with pembrolizumab exposure may represent an immunologic etiology. These adverse events may occur shortly after the first dose or several months after the last dose of treatment. Pembrolizumab must be withheld for drug-related toxicities and severe or life-threatening AEs as per Table 4 below.

Table 4 Dose Modification and Toxicity Management Guidelines for Immune-related AEs Associated with Pembrolizumab

General instructions:

1. Corticosteroid taper should be initiated upon AE improving to Grade 1 or less and continue to taper over at least 4 weeks.
2. For situations where pembrolizumab has been withheld, pembrolizumab can be resumed after AE has been reduced to Grade 1 or 0 and corticosteroid has been tapered. Pembrolizumab should be permanently discontinued if AE does not resolve within 12 weeks of last dose or corticosteroids cannot be reduced to ≤10 mg prednisone or equivalent per day within 12 weeks.
3. For severe and life-threatening irAEs, IV corticosteroid should be initiated first followed by oral steroid. Other immunosuppressive treatment should be initiated if irAEs cannot be controlled by corticosteroids.

Immune-related AEs	Toxicity grade or conditions (CTCAEv4.0)	Action taken to pembrolizumab	irAE management with corticosteroid and/or other therapies	Monitor and follow-up
Pneumonitis	Grade 2	Withhold	• Administer corticosteroids (initial dose of 1-2 mg/kg prednisone or equivalent) followed by taper	• Monitor participants for signs and symptoms of pneumonitis<br>• Evaluate participants with suspected pneumonitis with radiographic imaging and initiate corticosteroid treatment<br>• Add prophylactic antibiotics for opportunistic infections
	Grade 3 or 4, or recurrent Grade 2	Permanently discontinue		
Diarrhea / Colitis	Grade 2 or 3	Withhold	• Administer corticosteroids (initial dose of 1-2 mg/kg prednisone or equivalent) followed by taper	• Monitor participants for signs and symptoms of enterocolitis (ie, diarrhea, abdominal pain, blood or mucus in stool with or without fever) and of bowel perforation (ie, peritoneal signs and ileus).<br>• Participants with ≥ Grade 2 diarrhea suspecting colitis should consider GI consultation and performing endoscopy to rule out colitis.<br>• Participants with diarrhea/colitis should be advised to drink liberal quantities of clear fluids. If sufficient oral fluid intake is not feasible, fluid and electrolytes should be substituted via IV infusion.
	Grade 4	Permanently discontinue		

AST / ALT elevation or Increased bilirubin	Grade 2	Withhold	• Administer corticosteroids (initial dose of 0.5- 1 mg/kg prednisone or equivalent) followed by taper	• Monitor with liver function tests (consider weekly or more frequently until liver enzyme value returned to baseline or is stable
	Grade 3 or 4	Permanently discontinue	• Administer corticosteroids (initial dose of 1-2 mg/kg prednisone or equivalent) followed by taper	
Type 1 diabetes mellitus (T1DM) or Hyperglycemia	Newly onset T1DM or Grade 3 or 4 hyperglycemia associated with evidence of β-cell failure	Withhold	• Initiate insulin replacement therapy for participants with T1DM<br>• Administer anti-hyperglycemic in participants with hyperglycemia	• Monitor participants for hyperglycemia or other signs and symptoms of diabetes.
Hypophysitis	Grade 2	Withhold	• Administer corticosteroids and initiate hormonal replacements as clinically indicated.	• Monitor for signs and symptoms of hypophysitis (including hypopituitarism and adrenal insufficiency)
	Grade 3 or 4	Withhold or permanently discontinue[1]		
Hyperthyroidism	Grade 2	Continue	• Treat with non-selective beta-blockers (eg, propranolol) or thionamides as appropriate	• Monitor for signs and symptoms of thyroid disorders.
	Grade 3 or 4	Withhold or permanently discontinue[1]		
Hypothyroidism	Grade 2-4	Continue	• Initiate thyroid replacement hormones (eg, levothyroxine or liothyroinine) per standard of care	• Monitor for signs and symptoms of thyroid disorders.
Nephritis and Renal dysfunction	Grade 2	Withhold	• Administer corticosteroids (prednisone 1-2 mg/kg or equivalent) followed by taper.	• Monitor changes of renal function
	Grade 3 or 4	Permanently discontinue		
Myocarditis	Grade 1 or 2	Withhold	• Based on severity of AE administer corticosteroids	• Ensure adequate evaluation to confirm etiology and/or exclude other causes
	Grade 3 or 4	Permanently discontinue		

<table>
<tr><td rowspan="3">All other immune-related AEs</td><td>Intolerable/ persistent Grade 2</td><td>Withhold</td><td rowspan="3">• Based on type and severity of AE administer corticosteroids</td><td rowspan="3">• Ensure adequate evaluation to confirm etiology and/or exclude other causes</td></tr>
<tr><td>Grade 3</td><td>Withhold or discontinue based on the type of event. Events that require discontinuation include and not limited to: Gullain-Barre Syndrome, encephalitis</td></tr>
<tr><td>Grade 4 or recurrent Grade 3</td><td>Permanently discontinue</td></tr>
<tr><td colspan="5">1. Withhold or permanently discontinue pembrolizumab is at the discretion of the investigator or treating physician.
NOTE:
For participants with Grade 3 or 4 immune-related endocrinopathy where withhold of pembrolizumab is required, pembrolizumab may be resumed when AE resolves to ≤ Grade 2 and is controlled with hormonal replacement therapy or achieved metabolic control (in case of T1DM).</td></tr>
</table>

5.2.2 Timing of Dose Administration

Trial treatment should be administered on Day 1 of each cycle after all procedures/assessments have been completed as detailed on the Trial Flow Chart (Section 6.0). Trial treatment may be administered up to 3 days before or after the scheduled Day 1 of each cycle due to administrative reasons.

Dosing interruptions are permitted in the case of medical / surgical events or logistical reasons not related to study therapy (e.g., elective surgery, unrelated medical events, patient vacation, and/or holidays). Subjects should be placed back on study therapy within 3 weeks of the scheduled interruption, unless otherwise discussed with the Sponsor. The reason for interruption should be documented in the patient's study record.

All trial treatments will be administered on an outpatient basis.

Pembrolizumab 200 mg will be administered as a 30 minute IV infusion every 3 weeks. Sites should make every effort to target infusion timing to be as close to 30 minutes as possible. However, given the variability of infusion pumps from site to site, a window of -5 minutes and +10 minutes is permitted (i.e., infusion time is 30 minutes: -5 min/+10 min).

The Pharmacy Manual contains specific instructions for the preparation of the pembrolizumab infusion fluid and administration of infusion solution.

5.2.3 Trial Blinding/Masking

This is an open-label trial; therefore, the Sponsor, investigator and subject will know the treatment administered.

5.3 Randomization or Treatment Allocation

Not applicable.

5.4 Stratification

Not applicable.

5.5 Concomitant Medications/Vaccinations (allowed & prohibited)

Medications or vaccinations specifically prohibited in the exclusion criteria are not allowed during the ongoing trial. If there is a clinical indication for one of these or other medications or vaccinations specifically prohibited during the trial, discontinuation from trial therapy or vaccination may be required. The investigator should discuss any questions regarding this with the Principal Investigator. The final decision on any supportive therapy or vaccination rests with the investigator and/or the subject's primary physician.

5.5.1 Acceptable Concomitant Medications

All treatments that the investigator considers necessary for a subject's welfare may be administered at the discretion of the investigator in keeping with the community standards of medical care. All concomitant medication will be recorded on the electronic case report form (eCRF) including all prescription, over-the-counter (OTC), herbal supplements, and IV medications and fluids. If changes occur during the trial period, documentation of drug dosage, frequency, route, and date may also be included on the CRF.

All concomitant medications received within 28 days before the first dose of trial treatment and 30 days after the last dose of trial treatment should be recorded. Concomitant medications administered after 30 days after the last dose of trial treatment should be recorded for Serious Adverse Events (SAEs) as defined in Section 7.2.

5.5.2 Prohibited Concomitant Medications

Subjects are prohibited from receiving the following therapies during the Screening and Treatment Phase (including retreatment for post-complete response relapse) of this trial:

- Antineoplastic systemic chemotherapy or biological therapy
- Immunotherapy not specified in this protocol
- Chemotherapy not specified in this protocol
- Investigational agents other than pembrolizumab
- Radiation therapy
    - Note: Radiation therapy to a symptomatic solitary lesion or to the brain may be allowed at the investigator's discretion.
- Live vaccines within 30 days prior to the first dose of trial treatment and while participating in the trial. Examples of live vaccines include, but are not limited to, the following: measles, mumps, rubella, varicella/zoster, yellow fever, rabies, BCG, and typhoid vaccine.
- Systemic glucocorticoids for any purpose other than to modulate symptoms from an event of suspected immunologic etiology. The use of physiologic doses of corticosteroids may be approved after consultation with the Principal Investigator. Inhaled steroids for management of asthma, and prophylactic corticosteroids to avoid allergic reactions (e.g. to IV contrast dye) are permitted.

Subjects who, in the assessment by the investigator, require the use of any of the aforementioned treatments for clinical management should be removed from the trial. Subjects may receive other medications that the investigator deems to be medically necessary.

The Exclusion Criteria describes other medications which are prohibited in this trial.

There are no prohibited therapies during the Post-Treatment Follow-up Phase.

5.6 Rescue Medications & Supportive Care

5.6.1 Supportive Care Guidelines

Subjects should receive appropriate supportive care measures as deemed necessary by the treating investigator. See Section 5.2.1.2 for toxicity management guidelines for immune-related AEs associated with pembrolizumab. Where appropriate, these guidelines include the use of oral or intravenous treatment with corticosteroids as well as additional anti-inflammatory agents if symptoms do not improve with administration of corticosteroids. Note that several courses of steroid tapering may be necessary as symptoms may worsen when the steroid dose is decreased. For each disorder, attempts should be made to rule out other causes such as metastatic disease or bacterial or viral infection, which might require additional supportive care. The treatment guidelines are intended to be applied when the investigator determines the events to be related to pembrolizumab.

It may be necessary to perform conditional procedures such as bronchoscopy, endoscopy, or skin photography as part of evaluation of the event.

Management of Infusion Reactions: Signs and symptoms usually develop during or shortly after drug infusion and generally resolve completely within 24 hours of completion of infusion.

Table 5 below shows treatment guidelines for subjects who experience an infusion reaction associated with administration of pembrolizumab.

Table 5 Infusion Reaction Treatment Guidelines

NCI CTCAE Grade	Treatment	Premedication at subsequent dosing
Grade 1<br>Mild reaction; infusion interruption not indicated; intervention not indicated	Increase monitoring of vital signs as medically indicated until the subject is deemed medically stable in the opinion of the investigator.	None
Grade 2<br>Requires infusion interruption but responds promptly to symptomatic treatment (e.g., antihistamines, NSAIDS, narcotics, IV fluids); prophylactic medications indicated for < =24 hrs	**Stop Infusion and monitor symptoms.**<br>Additional appropriate medical therapy may include but is not limited to:<br>IV fluids<br>Antihistamines<br>NSAIDS<br>Acetaminophen<br>Narcotics<br>Increase monitoring of vital signs as medically indicated until the subject is deemed medically stable in the opinion of the investigator.<br>If symptoms resolve within one hour of stopping drug infusion, the infusion may be restarted at 50% of the original infusion rate (e.g., from 100 mL/hr to 50 mL/hr). Otherwise dosing will be held until symptoms resolve and the subject should be premedicated for the next scheduled dose.<br>**Subjects who develop Grade 2 toxicity despite adequate premedication should be permanently discontinued from further trial treatment administration.**	Subject may be premedicated 1.5h (± 30 minutes) prior to infusion of pembrolizumab with:<br><br>Diphenhydramine 50 mg po (or equivalent dose of antihistamine).<br><br>Acetaminophen 500-1000 mg po (or equivalent dose of antipyretic).
Grades 3 or 4	**Stop Infusion.**	No subsequent dosing

NCI CTCAE Grade	Treatment	Premedication at subsequent dosing
Grade 3:<br>Prolonged (i.e., not rapidly responsive to symptomatic medication and/or brief interruption of infusion); recurrence of symptoms following initial improvement; hospitalization indicated for other clinical sequelae (e.g., renal impairment, pulmonary infiltrates)<br><br>Grade 4:<br>Life-threatening; pressor or ventilatory support indicated	Additional appropriate medical therapy may include but is not limited to:<br>IV fluids<br>Antihistamines<br>NSAIDS<br>Acetaminophen<br>Narcotics<br>Oxygen<br>Pressors<br>Corticosteroids<br>Epinephrine<br><br>Increase monitoring of vital signs as medically indicated until the subject is deemed medically stable in the opinion of the investigator.<br>Hospitalization may be indicated.<br>**Subject is permanently discontinued from further trial treatment administration.**	
Appropriate resuscitation equipment should be available in the room and a physician readily available during the period of drug administration.		

5.7 Diet/Activity/Other Considerations

5.7.1 Diet

Subjects should maintain a normal diet unless modifications are required to manage an AE such as diarrhea, nausea or vomiting.

5.7.2 Contraception

Pembrolizumab may have adverse effects on a fetus in utero. Furthermore, it is not known if pembrolizumab has transient adverse effects on the composition of sperm. Non-pregnant, non-breast-feeding women may be enrolled if they are willing to use 2 methods of birth control or are considered highly unlikely to conceive. Highly unlikely to conceive is defined as 1) surgically sterilized, or 2) postmenopausal (a woman who is ≥45 years of age and has not had menses for greater than 1 year will be considered postmenopausal), or 3) not heterosexually active for the duration of the study. The two birth control methods can be either two barrier methods or a barrier method plus a hormonal method to prevent pregnancy. Subjects should start using birth control from study Visit 1 throughout the study period up to 120 days after the last dose of study therapy.

The following are considered adequate barrier methods of contraception: diaphragm, condom (by the partner), copper intrauterine device, sponge, or spermicide. Appropriate hormonal contraceptives will include any registered and marketed contraceptive agent that contains an estrogen and/or a progestational agent (including oral, subcutaneous, intrauterine, or intramuscular agents).

Subjects should be informed that taking the study medication may involve unknown risks to the fetus (unborn baby) if pregnancy were to occur during the study. In order to participate in the study they must adhere to the contraception requirement (described above) for the duration

of the study and during the follow-up period defined in section 7.2.2-Reporting of Pregnancy and Lactation to the Sponsor and to Merck. If there is any question that a subject will not reliably comply with the requirements for contraception, that subject should not be entered into the study.

5.7.3 Use in Pregnancy

If a subject inadvertently becomes pregnant while on treatment with pembrolizumab, the subject will immediately be removed from the study. The site will contact the subject at least monthly and document the subject's status until the pregnancy has been completed or terminated. The outcome of the pregnancy will be reported to the Sponsor and to Merck without delay and within 24 hours to the Sponsor and within 2 working days to Merck if the outcome is a serious adverse experience (e.g., death, abortion, congenital anomaly, or other disabling or life-threatening complication to the mother or newborn).

The study investigator will make every effort to obtain permission to follow the outcome of the pregnancy and report the condition of the fetus or newborn to the Sponsor. If a male subject impregnates his female partner the study personnel at the site must be informed immediately and the pregnancy reported to the Sponsor and to Merck and followed as described above and in Section 7.2.2.

5.7.4 Use in Nursing Women

It is unknown whether pembrolizumab is excreted in human milk. Since many drugs are excreted in human milk, and because of the potential for serious adverse reactions in the nursing infant, subjects who are breast-feeding are not eligible for enrollment.

5.8 Subject Withdrawal/Discontinuation Criteria

Subjects may withdraw consent at any time for any reason or be dropped from the trial at the discretion of the investigator should any untoward effect occur. In addition, a subject may be withdrawn by the investigator or the Sponsor if enrollment into the trial is inappropriate, the trial plan is violated, or for administrative and/or other safety reasons. Specific details regarding discontinuation or withdrawal are provided in Section 7.1.4 – Other Procedures.

A subject must be discontinued from the trial for any of the following reasons:

- The subject or legal representative (such as a parent or legal guardian) withdraws consent.

- Confirmed radiographic disease progression

    Note: For unconfirmed radiographic disease progression, please see Section 7.1.2.6

    Note: A subject may be granted an exception to continue on treatment with confirmed radiographic progression if clinically stable or clinically improved, please see Section 7.1.2.6

- Unacceptable adverse experiences as described in Section 5.2.1.2
- Intercurrent illness that prevents further administration of treatment
- Investigator's decision to withdraw the subject
- The subject has a confirmed positive serum pregnancy test
- Noncompliance with trial treatment or procedure requirements
- The subject is lost to follow-up
- Completed 35 administrations of pembrolizumab (approximately 2 years).

  Note: The number of treatments is calculated starting with the first dose. Participants who stop pembrolizumab after receiving 35 doses may be eligible for retreatment if they progress after stopping study treatment provided they meet the requirements detailed in Section 7.1.5.3. Participants may be retreated in the Second Course Phase (Retreatment) for up to an additional 17 cycles (approximately 1 year).

- Administrative reasons

The End of Treatment and Follow-up visit procedures are listed in Section 6 (Protocol Flow Chart) and Section 7.1.5 (Visit Requirements). After the end of treatment, each subject will be followed for 30 days for adverse event monitoring (serious adverse events will be collected for 90 days after the end of treatment as described in Section 7.2.3.1). Subjects who discontinue for reasons other than progressive disease will have post-treatment follow-up for disease status until disease progression, initiating a non-study cancer treatment, withdrawing consent or becoming lost to follow-up. After documented disease progression each subject will be followed by telephone every 3 months for overall survival until death, withdrawal of consent, or the end of the study, whichever occurs first.

5.8.1 Discontinuation of Study Therapy after CR

Discontinuation of treatment may be considered for subjects who have attained a confirmed CR that have been treated for at least 8 cycles of pembrolizumab and had at least two treatments with pembrolizumab beyond the date when the initial CR was declared. Subjects who then experience radiographic disease progression may be eligible for up to 17 cycles (approximately 1 year) of additional treatment with pembrolizumab via the Second Course Phase at the discretion of the investigator if no cancer treatment was administered since the last dose of pembrolizumab, the subject meets the safety parameters listed in the Inclusion/Exclusion criteria, and the trial is open. Subjects will resume therapy at the same dose and schedule at the time of initial discontinuation. Additional details are provided in Section 7.1.5.3.

5.9 Subject Replacement Strategy

5.10 Clinical Criteria for Early Trial Termination

Early trial termination will be the result of the criteria specified below:

1. Quality or quantity of data recording is inaccurate or incomplete

2. Poor adherence to protocol and regulatory requirements

3. Incidence or severity of adverse drug reaction in this or other studies indicates a potential health hazard to subjects

4. Plans to modify or discontinue the development of the study drug

In the event of Merck decision to no longer supply study drug, ample notification will be provided so that appropriate adjustments to subject treatment can be made.

6.0 TRIAL FLOW CHART

6.1 Study Flow Chart (Table 6)

**Trial Period:**	**Screening Phase**		**Treatment Cycles (3-Week Cycles)**								**End of Treatment**	**Post-Treatment**		
							To be repeated beyond 8 cycles							
Treatment Cycle/Title:	Screening (Visit 1)	Screening (Visit 2)	1	2	3	4	5	6	7	8	Discon	Safety Follow-up	Follow Up Visits	Survival Follow-Up[a]
Scheduling Window (Days)[b]:	-28 to -1	-10 to -1	± 3	± 3	± 3	± 3	± 3	± 3	± 3	± 3	At time of Discon	30 days post last dose (± 7 days)	Every 12 weeks post discon (± 7 days)	Every 12 weeks (± 7 days)
**Administrative Procedures**														
Informed Consent	X													
Inclusion/Exclusion Criteria	X													
Demographics and Medical History	X													
Prior and Concomitant Medication Review	X	X	X	X	X	X	X	X	X	X	X	X		
Trial Treatment Administration			X	X	X	X	X	X	X	X				
Post-study anticancer therapy status													X	X
Survival Status														X
**Clinical Procedures/Assessments**														
Review Adverse Events	X		X	X	X	X	X	X	X	X	X	X	X	
Full Physical Examination	X										X			
Directed Physical Examination			X	X	X	X	X	X	X	X				
Vital Signs (Height and Weight)[c]	X		X	X	X	X	X	X	X	X	X	X	X	
ECOG Performance Status		X	X	X	X	X	X	X	X	X	X	X	X	
**Laboratory Procedures/Assessments: analysis performed by LOCAL laboratory**														
Pregnancy Test – Urine or Serum β-HCG[d]		X												
PT/INR and aPTT[e]		X												

**Trial Period:**	**Screening Phase**		**Treatment Cycles (3-Week Cycles)**								**End of Treatment**	**Post-Treatment**		
							To be repeated beyond 8 cycles							
Treatment Cycle/Title:	Screening (Visit 1)	Screening (Visit 2)	1	2	3	4	5	6	7	8	Discon	Safety Follow-up	Follow Up Visits	Survival Follow-Up[a]
Scheduling Window (Days)[b]:	-28 to -1	-10 to -1	± 3	± 3	± 3	± 3	± 3	± 3	± 3	± 3	At time of Discon	30 days post last dose (± 7 days)	Every 12 weeks post discon (± 7 days)	Every 12 weeks (± 7 days)
CBC with Differential[f]		X		X	X	X	X	X	X	X	X	X		
Comprehensive Serum Chemistry Panel[f]		X		X	X	X	X	X	X	X	X	X		
Urinalysis[f]		X		X		X		X[g]		X[g]		X		
T3, FT4 and TSH[f]		X		X		X		X[g]		X[g]		X		
**Efficacy Measurements**														
Tumor Imaging and RECIST Assessment	X				X[h]			X[h]			X[i]		X[j]	
**Archival Tissue Collection**														
Archival Tumor Collection (confirm that archival tissue is available)[k]	X													
**Correlative Studies**														
Fresh Tumor Biopsy[l]	X			X							X			
Normal DNA Sequence Control Blood[m]		X												
Circulating Tumor DNA Blood[m,o]		X			X			X			X[p]			
Proteomic Analysis Blood[m]		X			X						X[p]			
Immune Assessment for PBMC Blood[m]		X	X	X			X			X[n]	X[p]		X	
Immune Assessment for Serum Cytokines[m]		X	X	X	X	X	X	X	X	X	X[p]		X	

a. After documented local site assessed disease progression, or the start of new anticancer treatment; contacts are every 12 weeks (3 months) by telephone.

**Trial Period:**	**Screening Phase**		**Treatment Cycles (3-Week Cycles)**								**End of Treatment**	**Post-Treatment**		
							To be repeated beyond 8 cycles							
Treatment Cycle/Title:	Screening (Visit 1)	Screening (Visit 2)	1	2	3	4	5	6	7	8	Discon	Safety Follow-up	Follow Up Visits	Survival Follow-Up[a]
Scheduling Window (Days)[b]:	-28 to -1	-10 to -1	± 3	± 3	± 3	± 3	± 3	± 3	± 3	± 3	At time of Discon	30 days post last dose (± 7 days)	Every 12 weeks post discon (± 7 days)	Every 12 weeks (± 7 days)

b. In general, assessments/procedures are to be performed on Day 1 and prior to the dose of treatment for each cycle unless otherwise specified.

c. Height will be measured at visit 1 only.

d. For women of reproductive potential, a urine pregnancy test should be performed within 72 hours prior to first dose of trial treatment. If urine pregnancy results cannot be confirmed as negative, a serum pregnancy test performed by the local study site laboratory will be required. Pregnancy tests (serum and/or urine tests) should be repeated if required by local guidelines.

e. INR, PTT may be repeated pre-tumor biopsy if deemed clinically indicated.

f. After Cycle 1, laboratory samples can be collected up to 3 days prior to Day 1 of subsequent cycles.

g. To be repeated ever 2 cycles after Cycle 6.

h. The first on-study imaging time point will be performed at 9 weeks (63 ±7 days) after the date of first dose of trial treatment and then every 9 weeks (63 ± 7 days) thereafter or more frequently if clinically indicated. After 9 months, imaging frequency should be reduced to every 12 weeks (84 ±7 days). Local site investigator readings will be used for subject management. Imaging timing should follow calendar days and should not be adjusted for delays in cycle starts of pembrolizumab cycle frequencies.

i. In subjects who discontinue study therapy without local site confirmed disease progression, a radiologic evaluation should be performed at the time of treatment discontinuation (i.e., date of discontinue ± 4 week window). If a previous scan was obtained within 4 weeks prior to the date of discontinuation, then a scan at treatment discontinuation is not mandatory.

**Trial Period:**	**Screening Phase**		**Treatment Cycles (3-Week Cycles)**								**End of Treatment**	**Post-Treatment**		
							To be repeated beyond 8 cycles							
Treatment Cycle/Title:	Screening (Visit 1)	Screening (Visit 2)	1	2	3	4	5	6	7	8	Discon	Safety Follow-up	Follow Up Visits	Survival Follow-Up[a]
Scheduling Window (Days)[b]:	-28 to -1	-10 to -1	± 3	± 3	± 3	± 3	± 3	± 3	± 3	± 3	At time of Discon	30 days post last dose (± 7 days)	Every 12 weeks post discon (± 7 days)	Every 12 weeks (± 7 days)

j. In subjects who discontinue study therapy without documented local site disease progression, every effort should be made to continue monitoring their disease status by radiologic imaging every 12 weeks (84 ± 7 days) in the first year and every 24 weeks (168 ± 7days) after year 1 until (1) the start of new anti-cancer treatment, (2) disease progression as assessed by the local site assessment, (3) death, or (4) the end of the trial, whichever occurs first.

k. An archival tissue sample will be collected at any time point. Detailed instructions for tissue collection, process and shipment are provided in the Procedures Manual.

l. Fresh tumor biopsies should be done at these time points: 1) Pre-treatment at day -28 to day -1, 2) On-treatment during end of cycle 2 (any day during week 6 or earlier if patient is responding to the treatment and the Investigator deems it necessary), 3) Post-progression: for patients who responded (CR or PR) or had prolonged SD (>4 months) and subsequently progressed – the biopsy should be done within 4 weeks post progression. For handling and processing details for tumor biopsies, please refer to Table 7.

m. For details regarding handling and processing of blood-based biomarkers, please refer to Table 7.

n. For PBMC samples after cycle 5, please draw every 3 cycles (i.e. cycle 8, 11, 14, etc).

o. Assessment for PBMC may also be performed on these samples.

p. Correlative studies samples taken at discontinuation can be performed at a reasonable timeframe after date of discontinuation for logistical reasons.

Table 7 Correlative Studies Collection for INSPIRE

Markers	Days of sample collection	Sample	Processing	Shipment
**Tumor-based**				
**Priority 1: PD-L1 and other Immunohistochemistry**	Pre-treatment: Screening (baseline day -28 to day -1)<br><br>On-treatment: before end of Cycle 2 (during week 6 or earlier if patient is responding to the treatment and the Investigator deems it necessary)<br><br>Post-progression: for patients who responded or had prolonged SD (> 4 months) and subsequently progressed (within 4 weeks post progression)	1st core of tumor tissue	Formalin-fixed, paraffin embedded	PD-L1 will be done by QualTek – unstained slide(s) will be sent to QualTek
**Priority 2: DNA/RNA sequencing, Tumor microenvironment evaluation, TILs generation, PDX**	Pre-treatment: Screening (baseline day -28 to day -1)<br><br>On-treatment: before end of Cycle 2 (during week 6 or earlier if patient is responding to the treatment and the Investigator deems it necessary)<br><br>Post-progression: for patients who responded or had prolonged SD (> 4 months) and subsequently progressed (within 4 weeks post progression)	2nd,3rd,4th,5th and 6th cores of tumor tissue	Fresh processing	Immune Monitoring Laboratory, Princess Margaret Cancer Centre

DNA, RNA, frozen tissue, FFPE tissue blocks and slides may be transferred to the Translational Genomics Laboratory for analysis (generating NGS based libraries for sequencing).

**Blood-Based**				
**Normal DNA Sequence Control Blood Sample (1 sample)**	Pre-treatment: Screening (baseline day -28 to day -1)	One 10-mL whole blood once only (lavender tube – EDTA)	DNA extraction as a normal genome sequence control	Pugh Lab/Princess Margaret Genomics Centre
**Circulating Tumor DNA (multiple samples)**	Pre-treatment: Screening (baseline day -10 to day -1)<br><br>At every tumor imaging time point, see Table 6, point (h)<br><br>Post-progression: for patients who responded or had prolonged SD (> 4 months) and subsequently progressed (within 4 weeks post progression)	At each time-point, 30 mL of peripheral blood will be collected in lavender (EDTA) tubes	Plasma will be separated from the cell pellet within 2 hours. Cell-free DNA will be purified from clarified plasma using the Circulating Nucleic Acid Kit (Qiagen)	Pugh Lab/Princess Margaret Genomics Centre
**Proteomic Analysis (3 samples in total)**	Pre-treatment: Screening (baseline day -10 to day -1)<br><br>On-treatment: before end of Cycle 3 (during week 9)<br><br>Post-progression: for patients who responded or had prolonged SD (> 4 months) and subsequently progressed (within 4 weeks post progression)	One 6-ml red top (no anti-coagulant) tube at each of 3 time points	Sample will be centrifuged between1-2 hours of collection, to separate serum	Clinical Chemistry Lab of Diamandis/ Blasutig, University Health Network

**Immune Assessment: PBMC (multiple samples)**	Pre-treatment: Screening (baseline day -10 to day -1) and day of treatment prior to dosing.<br><br>On-treatment: prior to dosing on cycle 2 and then prior to dosing on cycle 5 and then every 3 cycles thereafter while on study (i.e. cycle 8, 11, 14, etc). For those patients who discontinue drug but remain on study, a sample will be obtained approximately every 12 weeks till off study	Three 10-ml green top (sodium heparin) tubes at each time point.	Samples will be ficolled to harvest PBMC within 18 hours.	Immune Monitoring Laboratory, Princess Margaret Cancer Centre
**Immune Assessment: Serum (multiple samples)**	Pre-treatment: Screening (baseline day -10 to day -1) and day of treatment prior to dosing.<br><br>On-treatment: prior to dosing with each cycle. For those patients who discontinue drug but remain on study, a sample will be obtained approximately every 12 weeks till off study	Two 4-ml SST tube	Sample will be centrifuged between 1-2 hours of collection, to separate serum.	Immune Monitoring Laboratory, Princess Margaret Cancer Centre

7.0 TRIAL PROCEDURES

7.1 Trial Procedures

The Trial Flow Chart - Section 6.0 summarizes the trial procedures to be performed at each visit. Individual trial procedures are described in detail below. It may be necessary to perform these procedures at unscheduled time points if deemed clinically necessary by the investigator.

Furthermore, additional evaluations/testing may be deemed necessary by the Sponsor for reasons related to subject safety. In some cases, such evaluation/testing may be potentially sensitive in nature (e.g., HIV, Hepatitis C, etc.), and thus local regulations may require that additional informed consent be obtained from the subject. In these cases, such evaluations/testing will be performed in accordance with those regulations.

7.1.1 Administrative Procedures

7.1.1.1 Informed Consent

The Investigator must obtain documented consent from each potential subject prior to participating in a clinical trial.

7.1.1.1.1 General Informed Consent

Consent must be documented by the subject's dated signature or by the subject's legally acceptable representative's dated signature on a consent form along with the dated signature of the person conducting the consent discussion.

A copy of the signed and dated consent form should be given to the subject before participation in the trial.

The initial informed consent form, any subsequent revised written informed consent form and any written information provided to the subject must receive the REB's approval/favorable opinion in advance of use. The subject or his/her legally acceptable representative should be informed in a timely manner if new information becomes available that may be relevant to the subject's willingness to continue participation in the trial. The communication of this information will be provided and documented via a revised consent form or addendum to the original consent form that captures the subject's dated signature or by the subject's legally acceptable representative's dated signature.

Specifics about a trial and the trial population will be added to the consent form template at the protocol level.

The informed consent will adhere to REB requirements, applicable laws and regulations and Sponsor requirements.

7.1.1.2 Inclusion/Exclusion Criteria

All inclusion and exclusion criteria will be reviewed by the investigator or qualified designee to ensure that the subject qualifies for the trial.

7.1.1.3 Medical History

A medical history will be obtained by the investigator or qualified designee. Medical history will include all active conditions, and any condition diagnosed within the prior 10 years that are considered to be clinically significant by the Investigator. Details regarding the disease for which the subject has enrolled in this study will be recorded separately and not listed as medical history.

7.1.1.4 Prior and Concomitant Medications Review

7.1.1.4.1 Prior Medications

The investigator or qualified designee will review prior medication use, including any protocol-specified washout requirement, and record prior medication taken by the subject within 28 days before starting the trial. Treatment for the disease for which the subject has enrolled in this study will be recorded separately and not listed as a prior medication.

7.1.1.4.2 Concomitant Medications

The investigator or qualified designee will record medication, if any, taken by the subject during the trial. All medications related to reportable SAEs should be recorded as defined in Section 7.2.

7.1.1.5 Disease Details and Treatments

7.1.1.5.1 Disease Details

The investigator or qualified designee will obtain prior and current details regarding disease status. If molecular profiling results are available in study subjects, this information will also be collected.

7.1.1.5.2 Prior Treatment Details

The investigator or qualified designee will review all prior cancer treatments including systemic treatments, radiation and surgeries.

7.1.1.5.3 Subsequent Anti-Cancer Therapy Status

The investigator or qualified designee will review all new anti-neoplastic therapy initiated after the last dose of trial treatment. If a subject initiates a new anti-cancer therapy within 30 days after the last dose of trial treatment, the 30 day Safety Follow-up visit must occur before the

first dose of the new therapy. Once new anti-cancer therapy has been initiated the subject will move into survival follow-up.

7.1.1.6 Patient Registration

Prior to registering a patient, the site must have submitted all necessary regulatory documentation to the Tumor Immunotherapy Program Central Office. The eligibility checklist will only be sent once this has been received.

All patients will be screened by one of the investigators prior to entry on this study. An explanation of the study and discussion of the expected side effects and presentation of the informed consent document will take place.

No patient can receive protocol treatment until registration with the Central Office as taken place. All eligibility criteria must be met at the time of registration. There will be no exceptions. Any questions should be addressed with the Central Office prior to registration.

To register a patient, the following documents are to be completed by the research nurse or data manager and emailed to the TIP Central Office:

- Signed patient consent form
- Eligibility Checklist signed by the investigator

To complete the registration process, central office will review the checklist and once eligibility has been confirmed:

- Assign a patient serial number
- Confirm the patient dose
- Register the patient on the study
- Fax or e-mail the confirmation worksheet with the patient serial number and dose to the participating site

To ensure immediate attention is given to the emailed checklist, the site is advised to also call the Central office study coordinator. Patient registrations will be accepted between the hours of 9am to 5pm Monday to Friday, excluding Canadian statutory holidays when the central office will be closed.

All eligible patients enrolled in the study will be entered in a patient registration log maintained by the Tumor Immunotherapy Program central office. Following registration, patients should begin protocol treatment within 72 hours.

7.1.2 Clinical Procedures/Assessments

7.1.2.1 Adverse Event (AE) Monitoring

The investigator or qualified designee will assess each subject to evaluate for potential new or worsening AEs as specified in the Trial Flow Chart and more frequently if clinically indicated.

Adverse experiences will be graded and recorded throughout the study and during the follow-up period according to NCI CTCAE Version 4.03 (see Section 11.2). Toxicities will be characterized in terms regarding seriousness, causality, toxicity grading, and action taken with regard to trial treatment.

Please refer to section 7.2 for detailed information regarding the assessment and recording of AEs.

7.1.2.2 Full Physical Exam

The investigator or qualified designee will perform a complete physical exam during the screening period. Clinically significant abnormal findings should be recorded as medical history. A full physical exam should be performed during screening,

7.1.2.3 Directed Physical Exam

For cycles that do not require a full physical exam per the Trial Flow Chart, the investigator or qualified designee will perform a directed physical exam as clinically indicated prior to trial treatment administration.

7.1.2.4 Vital Signs

The investigator or qualified designee will take vital signs at screening, prior to the administration of each dose of trial treatment and at treatment discontinuation as specified in the Trial Flow Chart (Section 6.0). Vital signs should include temperature, pulse, respiratory rate, weight and blood pressure. Height will be measured at screening only.

7.1.2.5 Eastern Cooperative Oncology Group (ECOG) Performance Scale

The investigator or qualified designee will assess ECOG status (see Section 11.1) at screening, prior to the administration of each dose of trial treatment and discontinuation of trial treatment as specified in the Trial Flow Chart.

7.1.2.6 Tumor Imaging and Assessment of Disease

Immunotherapeutic agents such as pembrolizumab may produce antitumor effects by potentiating endogenous cancer-specific immune responses. The response patterns seen with such an approach may extend beyond the typical time course of responses seen with cytotoxic agents, and can manifest as a clinical response after an initial apparent increase in tumor burden (i.e. pseudoprogression) or even the appearance of new lesions.

In INSPIRE, objective response rates per RECIST 1.1 will be evaluated and will serve as the efficacy endpoint. However, as stated, standard RECIST-based assessement of disease progression may not provide an accurate assessment of response to immunotherapeutic agents

such as pembrolizumab. For this reason, irRECIST (Appendix 11.3) has been developed for use by the site investigator to inform on pembrolizumab treatment decisions.

7.1.2.7 Tumor Tissue Collection and Correlative Studies Blood Sampling

Please refer to Table 7 Correlative Studies Collection for INSPIRE for details of tumor tissue and blood collection.

7.1.3 Laboratory Procedures/Assessments

Details regarding specific laboratory procedures/assessments to be performed in this trial are provided below Laboratory Safety Evaluations (Hematology, Chemistry and Urinalysis)

Laboratory tests for hematology, chemistry, urinalysis, and others are specified in Table 8.

Table 8 Laboratory Tests

Hematology	Chemistry	Urinalysis	Other
Hematocrit	Albumin	Blood	Serum β-human chorionic gonadotropin†
Hemoglobin	Alkaline phosphatase	Glucose	(β-hCG) or urine pregnancy test†
Platelet count	Alanine aminotransferase (ALT)	Protein	PT (INR)
WBC (total and differential)	Aspartate aminotransferase (AST)	Specific gravity	aPTT
Red Blood Cell Count	Lactate dehydrogenase (LDH)		Free thriiodothyronine (T3)
Absolute Neutrophil Count	Carbon Dioxide		Free tyroxine (T4)
Absolute Lymphocyte Count	*(CO_2 or biocarbonate)*		Thyroid stimulating hormone (TSH)
	Total protein		
	Calcium		
	Chloride		Blood for correlative studies (see Table 7)
	Glucose		
	Phosphate		
	Potassium		
	Sodium		
	Magnesium		
	Total Bilirubin		
	Direct Bilirubin *(If total bilirubin is elevated above the upper limit of normal)*		
	Creatinine		
† Perform on women of childbearing potential only.			

Laboratory tests for screening or entry into the Second Course Phase should be performed within 10 days prior to the first dose of treatment. After Cycle 1, pre-dose laboratory procedures can be conducted up to 72 hours prior to dosing. Results must be reviewed by the investigator or qualified designee and found to be acceptable prior to each dose of trial treatment.

7.1.4 Other Procedures

7.1.4.1 Withdrawal/Discontinuation

When a subject discontinues/withdraws prior to trial completion, all applicable activities scheduled for the final trial visit should be performed at the time of discontinuation. Any adverse events which are present at the time of discontinuation/withdrawal should be followed in accordance with the safety requirements outlined in Section 7.2 - Assessing and Recording Adverse Events. Subjects who a) attain a CR or b) complete 35 treatments with pembrolizumab may discontinue treatment with the option of restarting treatment if they meet the criteria specified in Section 7.1.5.3. After discontinuing treatment following assessment of CR, these subjects should return to the site for a Safety Follow-up Visit (described in Section 7.1.5.1) and then proceed to the Follow-Up Period of the study (described in Section 7.1.5.2).

7.1.5 Visit Requirements

Visit requirements are outlined in Section 6.0 - Trial Flow Chart. Specific procedure-related details are provided above in Section 7.1 - Trial Procedures.

7.5.1.1 Safety Follow-Up Visit

The mandatory Safety Follow-Up Visit should be conducted approximately 30 days after the last dose of trial treatment or before the initiation of a new anti-cancer treatment, whichever comes first. All AEs that occur prior to the Safety Follow-Up Visit should be recorded. Subjects with an AE of Grade > 1 will be followed until the resolution of the AE to Grade 0-1 or until the beginning of a new anti-neoplastic therapy, whichever occurs first. SAEs that occur within 90 days of the end of treatment or before initiation of a new anti-cancer treatment should also be followed and recorded. Subjects who are eligible for retreatment with pembrolizumab (as described in Section 7.1.5.3) may have up to two safety follow-up visits, one after the Treatment Period and one after the Second Course Phase.

7.1.5.2 Follow-up Visits

Subjects who discontinue trial treatment for a reason other than disease progression will move into the Follow-Up Phase and should be assessed every 12 weeks (42 ± 7 days) by radiologic imaging to monitor disease status. After 1 year, the imaging time point will occur every 24 weeks (± 7 days). Every effort should be made to collect information regarding disease status until the start of new anti-neoplastic therapy, disease progression, death, end of the study or if the subject begins retreatment with pembrolizumab as detailed in Section 7.1.5.3. Information regarding post-study anti-neoplastic treatment will be collected if new treatment is initiated.

Subjects who are eligible to receive retreatment with pembrolizumab according to the criteria in Section 7.1.5.3 will move from the follow-up phase to the Second Course Phase when they experience disease progression.

7.1.5.2.1 Survival Follow-up

Once a subject experiences confirmed disease progression or starts a new anti-cancer therapy, the subject moves into the survival follow-up phase and should be contacted by telephone every 12 weeks to assess for survival status until death, withdrawal of consent, or the end of the study, whichever occurs first.

7.1.5.3 Second Course Phase (Retreatment Period)

All participants who stop study treatment with SD or better may be eligible for up to an additional 17 cycles (approximately 1 year) of pembrolizumab treatment if they progress after stopping study treatment from the initial treatment phase. This retreatment is termed the Second Course Phase of this study and is only available if the study remains open and the subject meets the following conditions:

- **Either**
  - Stopped initial treatment with pembrolizumab after attaining an investigator-determined confirmed CR according to RECIST 1.1, and
    - Was treated for at least 8 administrations of pembrolizumab before discontinuing therapy, and
    - Received at least two treatments with pembrolizumab beyond the date when the initial CR was declared

    OR

  - Had SD, PR or CR and stopped pembrolizumab treatment after completion of 35 administrations (approximately 2 years) for reasons other than disease progression or intolerability

AND

- Experienced an investigator-determined confirmed radiographic disease progression by RECIST 1.1 after stopping their initial treatment with pembrolizumab, and
- Did not receive any anti-cancer treatment since the last dose of pembrolizumab, and
- The participant meets all of the safety parameters listed in the inclusion criteria and none of the safety parameters listed in the exclusion criteria, and
- The study is ongoing

Note: patients must have measurable disease at the start of protocol treatment to be eligible for this provision.

Subjects who restart treatment will be retreated at the same dose and dose interval as when they last received pembrolizumab. Treatment will be administered for up to 17 additional administrations (approximately 1 year). Subjects who are re-treated must re-sign consent form.

Visit requirements are outlined in Section 6.0 – Trial Flow Chart.

7.2 Assessing and Recording Adverse Events

An adverse event is defined as any untoward medical occurrence in a patient or clinical investigation subject administered a pharmaceutical product and which does not necessarily have to have a causal relationship with this treatment. An adverse event can therefore be any unfavorable and unintended sign (including an abnormal laboratory finding, for example), symptom, or disease temporally associated with the use of a medicinal product or protocol-specified procedure, whether or not considered related to the medicinal product or protocol-specified procedure. Any worsening (i.e., any clinically significant adverse change in frequency and/or intensity) of a preexisting condition that is temporally associated with the use of the Merck's product, is also an adverse event.

Changes resulting from normal growth and development that do not vary significantly in frequency or severity from expected levels are not to be considered adverse events. Examples of this may include, but are not limited to, teething, typical crying in infants and children and onset of menses or menopause occurring at a physiologically appropriate time.

Merck product includes any pharmaceutical product, biological product, device, diagnostic agent or protocol-specified procedure, whether investigational (including placebo or active comparator medication) or marketed, manufactured by, licensed by, provided by or distributed by Merck for human use.

Adverse events may occur during the course of the use of Merck product in clinical trials or within the follow-up period specified by the protocol, or prescribed in clinical practice, from overdose (whether accidental or intentional), from abuse and from withdrawal.

Adverse events may also occur in screened subjects during any pre-allocation baseline period as a result of a protocol-specified intervention, including washout or discontinuation of usual therapy, diet, placebo treatment or a procedure.

Progression of the cancer under study is not considered an adverse event unless it is considered to be drug related by the investigator.

All adverse events will be recorded from the time the consent form is signed through 30 days following cessation of treatment and at each examination on the Adverse Event case report forms/worksheets. The reporting timeframe for adverse events meeting any serious criteria is described in section 7.2.3.1.

7.2.1 Definition of an Overdose for This Protocol and Reporting of Overdose to the Sponsor and to Merck

For purposes of this trial, an overdose of pembrolizumab will be defined as any dose of 1,000 mg or greater (≥5 times the indicated dose). No specific information is available on the treatment of overdose of pembrolizumab. Appropriate supportive treatment should be provided if clinically indicated. In the event of overdose, the subject should be observed closely for signs of toxicity. Appropriate supportive treatment should be provided if clinically indicated.

If an adverse event(s) is associated with ("results from") the overdose of a Merck product, the adverse event(s) is reported as a serious adverse event, even if no other seriousness criteria are met.

All reports of overdose with and without an adverse event must be reported within 24 hours to the Sponsor and within 2 working days hours to Merck Global Safety. (Attn: Worldwide Product Safety; [redacted])

7.2.2 Reporting of Pregnancy and Lactation to the Sponsor and to Merck

Although pregnancy and lactation are not considered adverse events, it is the responsibility of investigators or their designees to report any pregnancy or lactation in a subject (spontaneously reported to them), including the pregnancy of a male subject's female partner that occurs during the trial or within 120 days of completing the trial completing the trial, or 30 days following cessation of treatment if the subject initiates new anticancer therapy, whichever is earlier. All subjects and female partners of male subjects who become pregnant must be followed to the completion/termination of the pregnancy. Pregnancy outcomes of spontaneous abortion, missed abortion, benign hydatidiform mole, blighted ovum, fetal death, intrauterine death, miscarriage and stillbirth must be reported as serious events (Important Medical Events). If the pregnancy continues to term, the outcome (health of infant) must also be reported.

Such events must be reported within 24 hours to the Sponsor and within 2 working days to Merck Global Safety. (Attn: Worldwide Product Safety; [redacted])

7.2.3 Immediate Reporting of Adverse Events to the Sponsor and to Merck

7.2.3.1 Serious Adverse Events

A serious adverse event (SAE) is any adverse event occurring at any dose or during any use of Merck's product that:

- Results in death;
- Is life threatening;
- Results in persistent or significant disability/incapacity;
- Results in or prolongs an existing inpatient hospitalization;
- Is a congenital anomaly/birth defect;
- Is a new cancer (that is not a condition of the study);

- Is associated with an overdose;
- Is another important medical event

Any serious adverse event, or follow up to a serious adverse event, including death due to any cause other than progression of the cancer under study that occurs to any subject from the time the consent is signed through 90 days following cessation of treatment, or the initiation of new anti-cancer therapy, whichever is earlier, whether or not related to Merck product,

The investigator should inform the Tumor Immunotherapy Program Central Office of any **SAE within 24 hours of being made aware of the event**. All SAEs must be reported via a written report, signed by the Investigator, and emailed to the Tumor Immunotherapy Program Central Office at **TIP@uhn.ca** within 24-hours/1 business day at the latest on the following working day. The Sponsor is required to notify Merck Global Safety within 2 working days.

The initial report must be as complete as possible, including details of the current illness and serious adverse event, and an assessment of the causal relationship between the event and the investigational product(s). Information not available at the time of the initial report (e.g., an end date for the adverse event or laboratory values received after the report) must be documented on a follow-up SAE form.

All SAEs must be followed until they are resolved (return to normal or baseline values), stabilized, or the patient is lost to follow-up or deceased. Supplemental measurements and/or evaluations may be necessary to fully investigate the nature and/or causality of an AE or SAE. This may include additional laboratory tests, diagnostic procedures, or consultation with other healthcare professionals

A death on study requires reporting regardless of causality and attribution to treatment or other cause must be provided. Death due to progressive disease should be reported as grade 5 "Neoplasms benign, malignant and unspecified (incl cysts and polyps) - Other (Progressive Disease)" under the system organ class (SOC) of the same name. Evidence that the death was a manifestation of underlying disease (*e.g.*, radiological changes suggesting tumor growth or progression: clinical deterioration associated with a disease process) should be submitted. Deaths that occur beyond 30 days after the end of study drug administration/initiation of an alternate therapy, do not qualify as SAEs.

Additionally, any serious adverse event, considered by an investigator who is a qualified physician to be related to Merck product that is brought to the attention of the investigator at any time outside of the time period specified in the previous paragraph also must be reported immediately to the Sponsor and to Merck.

SAE reports and any other relevant safety information are to be forwarded to the Merck Global Safety facsimile number: [redacted].

7.2.4 Evaluating Adverse Events

An investigator who is a qualified physician will evaluate all adverse events according to the NCI Common Terminology for Adverse Events (CTCAE), version 4.03. Any adverse event which changes CTCAE grade over the course of a given episode will have each change of grade recorded on the adverse event case report forms/worksheets.

All adverse events regardless of CTCAE grade must also be evaluated for seriousness.

Table 9 Evaluating Adverse Events

An investigator who is a qualified physician, will evaluate all adverse events as to:

**V4.03 CTCAE Grading**	**Grade 1**	**Mild; asymptomatic or mid symptoms; clinical or diagnostic observations only; intervention not indicated.**
	**Grade 2**	**Moderate; minimal, local or noninvasive intervention indicated; limiting age-appropriate instrumental ADL.**
	**Grade 3**	**Severe or medically significant but not immediately life-threatening; hospitalization or prolongation or hospitalization indicated; disabling; limiting self-care ADL.**
	**Grade 4**	**Life threatening consequences; urgent intervention indicated.**
	**Grade 5**	**Death related to AE**
**Seriousness**	A serious adverse event is any adverse event occurring at any dose or during any use of Merck product that:	
	†**Results in death**; or	
	†**Is life threatening; or** places the subject, in the view of the investigator, at immediate risk of death from the event as it occurred (Note: This does not include an adverse event that, had it occurred in a more severe form, might have caused death.); or	
	†**Results in a persistent or significant disability/incapacity** (substantial disruption of one's ability to conduct normal life functions); or	
	†**Results in or prolongs an existing inpatient hospitalization** (hospitalization is defined as an inpatient admission, regardless of length of stay, even if the hospitalization is a precautionary measure for continued observation. (Note: Hospitalization [including hospitalization for an elective procedure] for a preexisting condition which has not worsened does not constitute a serious adverse event.); or	
	†**Is a congenital anomaly/birth defect** (in offspring of subject taking the product regardless of time to diagnosis);or	
	**Is a** new **cancer;** (that is not a condition of the study) **or**	
	**Is an overdose** (whether accidental or intentional). Any adverse event associated with an overdose is considered a serious adverse event.	
	**Other important medical events** that may not result in death, not be life threatening, or not require hospitalization may be considered a serious adverse event when, based upon appropriate medical judgment, the event may jeopardize the subject and may require medical or surgical intervention to prevent one of the outcomes listed previously (designated above by a †).	
**Duration**	Record the start and stop dates of the adverse event. If less than 1 day, indicate the appropriate length of time and units	
**Action taken**	Did the adverse event cause the Merck product to be discontinued?	
**Relationship to test drug**	Did the Merck product cause the adverse event? The determination of the likelihood that the Merck product caused the adverse event will be provided by an investigator who is a qualified physician. The investigator's signed/dated initials on the source document or worksheet that supports the causality noted on the AE form, ensures that a medically qualified assessment of causality was done. This initialed document must be retained for the required regulatory time frame. The criteria below are intended as reference guidelines to assist the investigator in assessing the likelihood of a relationship between the test drug and the adverse event based upon the available information.<br>**The following components are to be used to assess the relationship between the Merck product and the AE**; the greater the correlation with the components and their respective elements (in number and/or intensity), the more likely the Merck product caused the adverse event (AE):	
	**Exposure**	Is there evidence that the subject was actually exposed to the Merck product such as: reliable history, acceptable compliance assessment (pill count, diary, etc.), expected pharmacologic effect, or measurement of drug/metabolite in bodily specimen?
	**Time Course**	Did the AE follow in a reasonable temporal sequence from administration of the Merck product?<br>Is the time of onset of the AE compatible with a drug-induced effect (applies to trials with investigational medicinal product)?
	**Likely Cause**	Is the AE not reasonably explained by another etiology such as underlying disease, other drug(s)/vaccine(s), or other host or environmental factors

<table>
<tr><td rowspan="3">Relationship to Merck product (continued)</td><td colspan="2">The following components are to be used to assess the relationship between the test drug and the AE: (continued)</td></tr>
<tr><td>Dechallenge</td><td>Was the Merck product discontinued or dose/exposure/frequency reduced?
If yes, did the AE resolve or improve?
If yes, this is a positive dechallenge. If no, this is a negative dechallenge.
(Note: This criterion is not applicable if: (1) the AE resulted in death or permanent disability; (2) the AE resolved/improved despite continuation of the Merck product; or (3) the trial is a single-dose drug trial); or (4) Merck product(s) is/are only used one time.)</td></tr>
<tr><td>Rechallenge</td><td>Was the subject re-exposed to the Merck product in this study?
If yes, did the AE recur or worsen?
If yes, this is a positive rechallenge. If no, this is a negative rechallenge.
(Note: This criterion is not applicable if: (1) the initial AE resulted in death or permanent disability, or (2) the trial is a single-dose drug trial); or (3) Merck product(s) is/are used only one time).
NOTE: IF A RECHALLENGE IS PLANNED FOR AN ADVERSE EVENT WHICH WAS SERIOUS AND WHICH MAY HAVE BEEN CAUSED BY THE MERCK PRODUCT, OR IF REEXPOSURE TO THE MERCK PRODUCT POSES ADDITIONAL POTENTIAL SIGNIFICANT RISK TO THE SUBJECT, THEN THE RECHALLENGE MUST BE APPROVED IN ADVANCE BY THE U.S. CLINICAL MONITOR AS PER DOSE MODIFICATION GUIDELINES IN THE PROTOCOL.</td></tr>
<tr><td></td><td>Consistency with Trial Treatment Profile</td><td>Is the clinical/pathological presentation of the AE consistent with previous knowledge regarding the Merck product or drug class pharmacology or toxicology?</td></tr>
<tr><td colspan="3">The assessment of relationship will be reported on the case report forms /worksheets by an investigator who is a qualified physician according to his/her best clinical judgment, including consideration of the above elements.</td></tr>
<tr><td colspan="2">Record one of the following</td><td>Use the following scale of criteria as guidance (not all criteria must be present to be indicative of a Merck product relationship).</td></tr>
<tr><td colspan="2">Yes, there is a reasonable possibility of Merck product relationship.</td><td>There is evidence of exposure to the Merck product. The temporal sequence of the AE onset relative to the administration of the Merck product is reasonable. The AE is more likely explained by the Merck product than by another cause.</td></tr>
<tr><td colspan="2">No, there is not a reasonable possibility Merck product relationship</td><td>Subject did not receive the Merck product OR temporal sequence of the AE onset relative to administration of the Merck product is not reasonable OR there is another obvious cause of the AE. (Also entered for a subject with overdose without an associated AE.)</td></tr>
</table>

7.2.5 Sponsor Responsibility for Reporting Adverse Events to Health Canada

All adverse drug reactions that are **Serious, Unexpected, and at minimum Possibly related to the study drug**, and have not been previously reported in the Investigator's Brochure, or reference information document will be promptly reported to Health Canada in writing by the Tumor Immunotherapy Program. This will include a clear description of the suspected reaction and an assessment as to whether the event is thought to be drug or disease related. The Tumor Immunotherapy Program is responsible for reporting all Serious Adverse Events that are both unexpected and related to study drug to Canadian regulatory authorities. They will notify Health Canada by telephone or fax of any unexpected fatal or life threatening experience associated with the use of the drugs as soon as possible but no later than 7 calendar days after initial receipt of the information.

7.2.6 Responsibility for Reporting Adverse Events to the Research Ethics Board

The Principal Investigator is required to notify his/her Research Ethics Board of a serious adverse event in accordance with institutional policy. Documentation from the REB of receipt of these reportable events must be kept on file in each institution's regulatory binder.

8.0 STATISTICAL ANALYSIS PLAN

8.1 Statistical Analysis Plan

A total of approximately 100 evaluable patients will be recruited on this study. Each of the following 5 groups will enroll approximately 20 patients:

A Squamous Cell Cancer of Head and Neck (SCCHN)
B Triple Negative Breast Cancer (TNBC)
C Epithelial Ovarian Cancer (EOC) Type II
D Malignant Melanoma (MM)
E Advanced Solid Tumors

Subjects will be considered evaluable if they have tumor sample available from the screening and on-treatment biopsy time-points of acceptable quality and quantity for analysis, as assessed by study site correlatives team (an estimate of 40 additional patients are required to yield 100 evaluable patients by this definition).

8.2 Biomarker Endpoint Analysis

The primary objective(s), changes of biomarkers in genomic and immune landscapes in patients before and after treatment will be compared between responders and non-responders by using T-test or Wilcoxon's test.

For all the other objectives, summary statistics, such as the mean, median, counts and proportion, will be used to summarize the patients. Overall response rate, duration of response,

rate of 1 year PFS and overall survival (OS) will be reported. Survival estimates will be computed using the Kaplan-Meier method. Potential association between variables will be measured using Pearson correlation coefficients, chi-square tests, one- or two-sample t-tests or logistic regression analyses as appropriate. Non-parametric tests such as Spearman correlation coefficients, Fisher's exact tests and Wilcoxon rank sum tests may be substituted if necessary. Ninety-five percent confidence intervals will be constructed and selected results will be illustrated using figures and plots.

Frequency and severity of adverse events will be tabulated using counts and proportions detailing frequently occurring, serious and severe events of interest.

If sufficient numbers of patients have a clinical response, the treatment-related marker effect on clinical response will be evaluated. Logistic regression analysis will be used to test whether the mean marker value difference (pre-treatment biopsy value - post-treatment biopsy value) and the mean marker value at the pre-treatment biopsy are statistically significant predictors of clinical response. Plots and tables of the data will be presented to visually inspect the findings.

These tests will be considered purely exploratory in nature and exact p-values given. Thus, no p-value adjustment will occur; however, significant results will be interpreted understanding the exploratory nature of these tests and the increased probability of observing statistically significant results under the null hypothesis due to multiple testing

9.0 LABELING, PACKAGING, STORAGE AND RETURN OF CLINICAL SUPPLIES

9.1 Investigational Product

The investigator shall take responsibility for and shall take all steps to maintain appropriate records and ensure appropriate supply, storage, handling, distribution and usage of investigational product in accordance with the protocol and any applicable laws and regulations.

Clinical Supplies will be provided by Merck as summarized in Table 10.

Table 10 Product Descriptions

Product Name & Potency	Dosage Form
Pembrolizumab 50 mg	Lyophilized Powder for Injection
Pembrolizumab 100 mg/ 4mL	Solution for Injection

9.2 Packaging and Labeling Information

Clinical supplies will be affixed with a clinical label in accordance with regulatory requirements.

9.3 Clinical Supplies Disclosure

This trial is open-label; therefore, the subject, the trial site personnel, the Sponsor and/or designee are not blinded to treatment. Drug identity (name, strength) is included in the label text; random code/disclosure envelopes or lists are not provided.

9.4 Storage and Handling Requirements

Clinical supplies must be stored in a secure, limited-access location under the storage conditions specified on the label.

Receipt and dispensing of trial medication must be recorded by an authorized person at the trial site.

Clinical supplies may not be used for any purpose other than that stated in the protocol.

9.5 Returns and Reconciliation

The investigator is responsible for keeping accurate records of the clinical supplies received from Merck or designee, the amount dispensed to and returned by the subjects and the amount remaining at the conclusion of the trial.

Upon completion or termination of the study, all unused and/or partially used investigational product will be destroyed at the site per institutional policy. It is the Investigator's responsibility to arrange for disposal of all empty containers, provided that procedures for proper disposal have been established according to applicable federal, state, local and institutional guidelines and procedures, and provided that appropriate records of disposal are kept.

10.0 ADMINISTRATIVE AND REGULATORY DETAILS

10.1 Compliance with Trial Registration and Results Posting Requirements

Under the terms of the Food and Drug Administration Modernization Act (FDAMA) and the Food and Drug Administration Amendments Act (FDAAA), the Sponsor of the trial is solely responsible for determining whether the trial and its results are subject to the requirements for submission to the Clinical Trials Data Bank, http://www.clinicaltrials.gov. Information posted will allow subjects to identify potentially appropriate trials for their disease conditions and pursue participation by calling a central contact number for further information on appropriate trial locations and trial site contact information.

10.2 Patient Protection

The responsible investigator will ensure that this study is conducted in agreement with either the Declaration of Helsinki (Tokyo, Venice, Hong Kong, Somerset West and Edinburgh amendments) or the laws and regulations of the country, whichever provides the greatest protection of the patient. The protocol has been written, and the study will be conducted according to the ICH Harmonized Tripartite Guideline for Good Clinical Practice (ref: http://www.fda.gov/cder/guidance/959fnl.pdf). The protocol will be approved by the Local, Regional or National Ethics Committees.

10.3 Direct Access to Source Data Documents

In accordance with federal regulations and GCP guidelines, the investigator should make available for direct access all trial-related records upon request of the sponsor or clinical monitor, auditor, REB, Health Canada or FDA.

10.4 Study Monitoring

This is an investigator initiated study and study monitoring will be performed by the Tumor Immunotherapy Program Central Office. Data in the Medidata Rave eCRFs will be reviewed on a regular basis and quality assurance measures will be performed. Electronic data queries as well as paper query letters may be issued to the site.

10.5 Quality Control and Quality Assurance

The Tumor Immunotherapy Program Central Office is responsible for ensuring that the trial is conducted and data are generated, documented, and reported in compliance with the protocol, GCP guidelines, and applicable regulatory requirements. A monitoring plan is in place for this investigator-initiated trial, at least 10% of patients on study will be monitored to ensure data quality.

10.6 Data Management Guidelines

10.6.1 Case Report Form Completion

At the time of patient registration, the paper Eligibility Checlist CRF must be completed using black or blue ink. Any errors must be crossed out so that the original entry is still visible, the correction clearly indicated and then initialed and dated by the individual making the correction.

This study will utilize electronic data capture using the Medidata Rave® platform. Site staff access to Medidata Rave will be initiated at the time of site activation. eCRFs will be completed using any internet-ready computer, and each assigned user will have a password-protected secure sign-in. Study-specific document forms may be developed directly from eCRFs for ease

of data collection and can be considered as source. eCRF entries will be made on site at the participating centre.

10.6.2 Case Report Form Submission Schedule

eCRFs will be completed according to the schedule noted below. eCRF completion guidelines are available for the site. The Investigator must electronically sign and date a declaration on the electronic CRF attesting to his/her responsibility for the quality of all data entered and that the data represents a complete and accurate record of each patient's participation in the study.

Case Report Form	Submission Schedule
Eligibility Checklist	At the time of registration
Baseline eCRFs	Within 2 weeks of on study date
On Treatment (Cycle) eCRFs	Within 2 weeks of the end of each cycle of treatment
End of Treatment (EOT) eCRFs	Within 2 weeks of the patient coming off study therapy
30 Day Safety Follow-Up eCRF	Within 2 weeks of the patient visit/contact
12 Week Follow-Up eCRFs	Within 2 weeks of the radiologic assessment date
12 Week Survival Follow-Up eCRFs	Within 2 weeks of the patient visit/chart review every 12 weeks or death being known to the investigator, if applicable

10.6.3 Regulatory Requirements

- All required documents are to be submitted to the Tumor Immunotherapy Program Central Office.
- The Principal Investigator must submit a completed Qualified Investigator Undertaking (QIU).
- All investigators must have an up-to-date CV (signed within 2 years) on file with the Tumor Immunotherapy Program Central Office.
- Laboratory certification/accreditation and normal ranges are required
- Investigators and site staff are required to complete Medidata eCRF training modules depending on delegated tasks
- Consent forms must be reviewed by the Central Office before submission to the local regulatory ethics board (REB) and must include a statement that 1) information will be sent to, and 2) medical records will be reviewed by, the Tumor Immunotherapy Program Central Office.

- A Membership list of the local ethics board is required.
- A copy of the initial approval letter from the ethics board must be submitted to the Tumor Immunotherapy Program Central Office.
- A completed Site Participant List/Training Log is required and must be submitted to the Tumor Immunotherapy Program Central Office
- Continuing approval will be obtained at least yearly until follow-up on patients is completed

10.7 REB Composition

The participating centre will have on file with the Tumor Immunotherapy Program the composition of its REB. The composition and procedures of the REB must be compliant with the ICH-GCP Guidelines and be consistent with Canadian regulatory requirements.

10.8 Initial Approval

Documentation of full board approval of the initial protocol and the consent form must be received prior to local activation.

An REB Attestation Form (Health Canada) must be completed and signed by the REB Chair. This documentation or a comparable assurance must be received by the Tumor Immunotherapy Program central office before the centre can be locally activated.

10.9 Annual Re-Approvals

Annual re-approval is required for as long as the trial is open to patient accrual or patients are receiving protocol treatment or undergoing protocol mandated interventions.

10.10 Amendments / Revisions

All amendments or revisions to the protocol must undergo review by local REB prior to implementation. Amendments/revisions will be circulated to all participating sites in a standard format with clear instructions regarding REB review. If full board approval of an amendment is required it will be specified.

Amendments will be reviewed and approved by Health Canada prior to central implementation of the study, and by REB prior to local implementation, EXCEPT when the amendment eliminates an immediate hazard to clinical trial subjects.

10.11 REB Refusals

If an REB refuses to approve this protocol (or an amendment/revision to this protocol) the Tumor Immunotherapy Program must be notified immediately of the date of refusal and the reason(s) for the refusal.

10.12 Serious Adverse Events, Safety Updates, and Investigator Brochure Updates

During the course of the study serious adverse events, safety updates or investigator brochure updates may be sent to the Principal Investigator for reporting to your REB. If/when this occurs, documentation of REB submission of this information must be forwarded to the central office.

10.13 Informed Consent Document

The REB of an institution must approve the consent form document which will be used at that centre prior to its local activation; changes to the consent form in the course of the study will also require REB notification/approval.

It is essential that the consent form contain a clear statement that gives permission for 1) information to be sent to and 2) source medical records to be reviewed by the Tumor Immunotherapy Program and other agencies as necessary. In addition, the consent form should include all elements required by ICH-Good Clinical Practice Guidelines.

10.14 Consent Process/ Patient Eligibility

Patients who cannot give informed consent (i.e. mentally incompetent patients, or those physically incapacitated such as comatose patients) are not to be recruited into the study. Patients competent but physically unable to sign the consent form may have the document signed by their nearest relative or legal guardian. Each patient will be provided with a full explanation of the study before consent is requested.

10.15 Retention of Patient Records and Study Files

This study is conducted under a CTA with Health Canada, therefore ICH Good Clinical Practice guidelines apply. All essential documents should be retained until at least two years after the last approval of a marketing application in an ICH region and until there are no pending or contemplated marketing applications in ICH region or at least two years have elapsed since the formal discontinuation of clinical development of the investigational product or for 25 years, whichever is longer. These documents should be retained for a longer period however if required by the applicable regulatory requirements or by an agreement with the Tumor Immunotherapy Program. It is the responsibility of Tumor Immunotherapy Program to inform the investigator/institution as to when these documents no longer need to be retained. The investigator/institution should take measures to prevent accidental or premature destruction of these documents.

The Tumor Immunotherapy Program will notify all the trial investigators/institutions and all the regulatory authorities if clinical development of an investigational product discontinues or when trial related records are no longer needed.

11.0 APPENDICES

11.1 Table 11 ECOG Performance Status

Grade	Description
0	Normal activity. Fully active, able to carry on all pre-disease performance without restriction.
1	Symptoms, but ambulatory. Restricted in physically strenuous activity, but ambulatory and able to carry out work of a light or sedentary nature (e.g., light housework, office work).
2	In bed <50% of the time. Ambulatory and capable of all self-care, but unable to carry out any work activities. Up and about more than 50% of waking hours.
3	In bed >50% of the time. Capable of only limited self-care, confined to bed or chair more than 50% of waking hours.
4	100% bedridden. Completely disabled. Cannot carry on any self-care. Totally confined to bed or chair.
5	Dead.
* As published in Am. J. Clin. Oncol.: *Oken, M.M., Creech, R.H., Tormey, D.C., Horton, J., Davis, T.E., McFadden, E.T., Carbone, P.P.: Toxicity And Response Criteria Of The Eastern Cooperative Oncology Group. Am J Clin Oncol 5:649-655, 1982.* The Eastern Cooperative Oncology Group, Robert Comis M.D., Group Chair.	

11.2 Common Terminology Criteria for Adverse Events V4.03 (CTCAE)

The descriptions and grading scales found in the revised NCI Common Terminology Criteria for Adverse Events (CTCAE) version 4.03 will be utilized for adverse event reporting. (http://ctep.cancer.gov/reporting/ctc.html)

11.3 Response Evaluation Criteria in Solid Tumors (RECIST) 1.1 Criteria for Evaluating Response in Solid Tumors

RECIST version 1.1* will be used in this study for assessment of tumor response. While either CT or MRI may be utilized, as per RECIST 1.1, CT is the preferred imaging technique in this study.

* As published in the European Journal of Cancer:

E.A. Eisenhauer, P. Therasse, J. Bogaerts, L.H. Schwartz, D. Sargent, R. Ford, J. Dancey, S. Arbuck, S. Gwyther, M. Mooney, L. Rubinstein, L. Shankar, L. Dodd, R. Kaplan,

D. Lacombe, J. Verweij. New response evaluation criteria in solid tumors: Revised RECIST guideline (version 1.1). Eur J Cancer. 2009 Jan;45(2):228-47.

11.4 irRECIST

When radiologic imaging assessment by the investigator first shows disease progression by RECIST 1.1, the trial subject has the option of continuing treatment per below while awaiting radiologic local site confirmation of progression as assessed by repeat imaging ≥4 weeks later. In all cases, if RECIST-measurable disease is restricted to a solitary lesion, its neoplastic nature should be confirmed either by cytology/histology or by lesion progression on the next imaging examination.

The decision to continue trial treatment after the 1st evidence of disease progression is at the Investigator's discretion based on the clinical status of the subject as described in Table 12 below (Imaging and Treatment After 1st Radiologic Evidence of Disease Progression).

Clinically stable subjects may continue to receive trial treatment and tumor assessment should be repeated ≥ 4 weeks later in order to confirm local site disease progression by RECIST 1.1. Clinical stability is defined by the following criteria:

- Absence of signs and symptoms of clinically significant progression of disease, including worsening of laboratory values
- No decline in ECOG performance status
- Absence of rapid progression of disease
- Absence of progressive tumor at critical anatomical sites (e.g., CNS metastasis with potential for cord compression) requiring urgent alternative medical intervention

NOTE: Subjects exhibiting toxicity from trial therapy as outlined in Section 5.2.1.2 and 7.2 may NOT continue to receive trial therapy.

Table 12 Imaging and Treatment After 1st Radiologic Evidence of Disease Progression

	Clinically Stable		Clinically Unstable	
	Imaging	Treatment	Imaging	Treatment
1st radiologic evidence of disease progression (RECIST 1.1 by	Repeat imaging at ≥4 weeks at site to confirm disease progression	May continue trial treatment at the investigator's discretion while awaiting	Repeat imaging at ≥4 weeks at site to confirm disease progression per	Discontinue treatment

local site assessment)		confirmatory scan by site	physician discretion only	
Repeat scan confirms disease progression (RECIST 1.1 by local site assessment)	No additional imaging required	Discontinue treatment	No additional imaging required	N/A
Repeat scan shows no evidence of disease progression (RECIST 1.1 by local site assessment)	Continue regularly scheduled imaging assessments	Continue trial treatment at the Investigator's discretion	Continue regularly scheduled imaging assessments	May restart trial if condition has improved and/or clinically stable per investigator's discretion

In determining whether or not the tumor burden has increased or decreased per irRECIST, the local site investigator should consider all target and non-target lesions as well as any incremental new lesion(s).
Scenarios where progressive disease is confirmed at repeat imaging if ANY of the following occur by irRECIST:

- Tumor burden remains ≥ 20% and at least 5 mm absolute increase compared to nadir
- Non-target disease resulting in initial diagnosis of progressive disease is worse (qualitative assessment)
- New lesion resulting in initial diagnosis of progressive disease is worse (qualitative assessment)
- Additional new lesion(s) since last evaluation
- Additional new non-target lesion progression since last evaluation

If repeat imaging confirms progressive disease due to any of the scenarios listed above, subjects will be discontinued from trial therapy (exception noted below).
Scenarios where progressive disease is not confirmed at repeat imaging if ALL of the following occur by irRECIST:

- Tumor burden is < 20 % or < 5 mm absolute increase compared to nadir
- Non-target disease resulting in initial diagnosis of progressive disease is stable or improved (qualitative assessment)
- New lesion resulting in initial diagnosis of progressive disease is stable or improved (qualitative assessment)

- No incremental new lesion(s) since last evaluation
- No incremental new non-target progression since last evaluation

If repeat local site imaging does not confirm progressive disease by irRECIST and the subject continues to be clinically stable, treatment may continue and follow the regular imaging schedule.

When feasible, subjects should not be discontinued until progression is confirmed by the local site investigator radiology assessment. This allowance to continue treatment despite initial radiologic progressive disease takes into account the observation that some subjects can have a transient tumor flare in the first few months after the start of immunotherapy, and then experience subsequent disease response. Subjects that are deemed clinically unstable are not required to have repeat tumor imaging for confirmation of PD.

References:

1. **Dunn GP, Koebel CM, Schreiber RD: Interferons, immunity and cancer immunoediting. Nat.Rev.Immunol. 6:836-848, 2006**
2. **Fridman WH, Pages F, Sautes-Fridman C, et al: The immune contexture in human tumours: impact on clinical outcome. Nat Rev Cancer 12:298-306, 2012**
3. **Wherry EJ: T cell exhaustion. Nat Immunol 12:492-9, 2011**
4. **Okazaki T, Chikuma S, Iwai Y, et al: A rheostat for immune responses: the unique properties of PD-1 and their advantages for clinical application. Nat Immunol 14:1212-8, 2013**
5. **Taube JM, Anders RA, Young GD, et al: Colocalization of inflammatory response with B7-h1 expression in human melanocytic lesions supports an adaptive resistance mechanism of immune escape. Sci Transl Med 4:127ra37, 2012**
6. **Spranger S, Spaapen RM, Zha Y, et al: Up-Regulation of PD-L1, IDO, and Tregs in the Melanoma Tumor Microenvironment Is Driven by CD8+ T Cells. Sci Transl Med 5:200ra116, 2013**
7. **Taube JM, Klein AP, Brahmer JR, et al: Association of PD-1, PD-1 ligands, and other features of the tumor immune microenvironment with response to anti-PD-1 therapy. Clin Cancer Res, 2014**
8. **Seiwert TY, Haddad RI, Gupta S, et al: Antitumor activity and safety of pembrolizumab in patients (pts) with advanced squamous cell carcinoma of the head and neck (SCCHN): Preliminary results from KEYNOTE-012 expansion cohort. J Clin Oncol 33:(suppl; abstr LBA6008), 2015**
9. **Nanda R, Chow LQ, Dees EC, et al: A phase Ib study of pembrolizumab (MK-3475) in patients with advanced triple-negative breast cancer. San Antonio Breast Cancer Symposium:abstr S1-09, 2014**
10. **Varga A, Piha-Paul SA, Ott PA, et al: Antitumor activity and safety of pembrolizumab in patients (pts) with PD-L1 positive advanced ovarian cancer: Interim results from a phase Ib study. J Clin Oncol 33:(suppl; abstr 5510), 2015**
11. **Robert C, Schachter J, Long GV, et al: Pembrolizumab versus Ipilimumab in Advanced Melanoma. N Engl J Med, 2015**
12. **Li H, Durbin R: Fast and accurate short read alignment with Burrows-Wheeler transform. Bioinformatics 25:1754-60, 2009**
13. **Cibulskis K, Lawrence MS, Carter SL, et al: Sensitive detection of somatic point mutations in impure and heterogeneous cancer samples. Nat Biotechnol 31:213-9, 2013**
14. **Koboldt DC, Zhang Q, Larson DE, et al: VarScan 2: somatic mutation and copy number alteration discovery in cancer by exome sequencing. Genome Res 22:568-76, 2012**
15. **Ramos A, al. e: Oncotator. at <http://www.broadinstitute.org/oncotator/>.**
16. **Favero F, Joshi T, Marquard AM, et al: Sequenza: allele-specific copy number and mutation profiles from tumor sequencing data. Ann Oncol 26:64-70, 2015**
17. **Roth A, Khattra J, Yap D, et al: PyClone: statistical inference of clonal population structure in cancer. Nat Methods 11:396-8, 2014**

18. Zhao W, He X, Hoadley KA, et al: Comparison of RNA-Seq by poly (A) capture, ribosomal RNA depletion, and DNA microarray for expression profiling. BMC Genomics 15:419, 2014

19. Wilkerson MD, Cabanski CR, Sun W, et al: Integrated RNA and DNA sequencing improves mutation detection in low purity tumors. Nucleic Acids Res 42:e107, 2014

20. Trapnell C, Roberts A, Goff L, et al: Differential gene and transcript expression analysis of RNA-seq experiments with TopHat and Cufflinks. Nat Protoc 7:562-78, 2012

21. Griffith M, Griffith OL, Mwenifumbo J, et al: Alternative expression analysis by RNA sequencing. Nat Methods 7:843-7, 2010

22. Wu Jea: SOAPfusion: a robust and effective computational fusion discovery tool for RNA-seq reads. Bioinformatics btt522 (2013). doi:10.1093/bioinformatics/btt522.

23. Li S, Tighe SW, Nicolet CM, et al: Multi-platform assessment of transcriptome profiling using RNA-seq in the ABRF next-generation sequencing study. Nat Biotechnol 32:915-25, 2014

24. Consortium SM-I: A comprehensive assessment of RNA-seq accuracy, reproducibility and information content by the Sequencing Quality Control Consortium. Nat Biotechnol 32:903-14, 2014

25. Consortium GT: The Genotype-Tissue Expression (GTEx) project. Nat Genet 45:580-5, 2013

26. Tumeh PC, Harview CL, Yearley JH, et al: PD-1 blockade induces responses by inhibiting adaptive immune resistance. Nature 515:568-71, 2014

27. Butler MO, Imataki O, Yamashita Y, et al: Ex vivo expansion of human CD8+ T cells using autologous CD4+ T cell help. PLoS One 7:e30229, 2012

28. Nguyen LT, Yen PH, Nie J, et al: Expansion and characterization of human melanoma tumor-infiltrating lymphocytes (TILs). PLoS One 5:e13940, 2010

29. Diaz LA, Jr., Williams RT, Wu J, et al: The molecular evolution of acquired resistance to targeted EGFR blockade in colorectal cancers. Nature 486:537-40, 2012

30. Oxnard GR, Paweletz CP, Kuang Y, et al: Noninvasive detection of response and resistance in EGFR-mutant lung cancer using quantitative next-generation genotyping of cell-free plasma DNA. Clin Cancer Res 20:1698-705, 2014

31. Newman AM, Bratman SV, To J, et al: An ultrasensitive method for quantitating circulating tumor DNA with broad patient coverage. Nat Med 20:548-54, 2014

32. Snyder A, Makarov V, Merghoub T, et al: Genetic Basis for Clinical Response to CTLA-4 Blockade in Melanoma. N Engl J Med 371:2189-2199, 2014

33. Lambin P, Rios-Velazquez E, Leijenaar R, et al: Radiomics: extracting more information from medical images using advanced feature analysis. Eur J Cancer 48:441-6, 2012

34. Kumar V, Gu Y, Basu S, et al: Radiomics: the process and the challenges. Magn Reson Imaging 30:1234-48, 2012

35. Aerts HJ, Velazquez ER, Leijenaar RT, et al: Decoding tumour phenotype by noninvasive imaging using a quantitative radiomics approach. Nat Commun 5:4006, 2014

36. Wolff AC, Hammond ME, Hicks DG, et al: Recommendations for human epidermal growth factor receptor 2 testing in breast cancer: American Society of Clinical Oncology/College of American Pathologists clinical practice guideline update. J Clin Oncol 31:3997-4013, 2013
