# Supplementary material for: Pan-cancer analysis of longitudinal metastatic tumors reveals genomic alterations and immune landscape dynamics associated with pembrolizumab sensitivity
Source: Nat Commun. 2021 Aug 26;12:5137. doi: 10.1038/s41467-021-25432-7 (PMC8390680; doi:10.1038/s41467-021-25432-7)
Supplement: Supplementary file 3 — Description of Additional Supplementary Files [file 41467_2021_25432_MOESM3_ESM.pdf]

Description of Additional Supplementary Files

File Name: Supplementary Data 1
Description: INSPIRE patient cancer type, clinical outcome, and data availability.

File Name: Supplementary Data 2
Description: Mutation enrichment in baseline tumors with high and low pembrolizumab molecular sensitivity.

File Name: Supplementary Data 3
Description: Differentially expressed genes after anti-PD1 treatment in HS/CB tumors.

File Name: Supplementary Data 4
Description: Differentially expressed genes after anti-PD1 treatment in LS tumors.

File Name: Supplementary Data 5
Description: Differentially regulated genes between HS/CB and LS tumors.

File Name: Supplementary Data 6
Description: GO biological processes terms enriched in selected DRGs.
