# Supplementary material for: Pan-cancer analysis of longitudinal metastatic tumors reveals genomic alterations and immune landscape dynamics associated with pembrolizumab sensitivity
Source: Nat Commun. 2021 Aug 26;12:5137. doi: 10.1038/s41467-021-25432-7 (PMC8390680; doi:10.1038/s41467-021-25432-7)
Supplement: Supplementary file 10 — Reporting Summary [file 41467_2021_25432_MOESM10_ESM.pdf]

nature research

Corresponding author(s): Trevor J. Pugh

Last updated by author(s): 16/7/21

Reporting Summary

Nature Research wishes to improve the reproducibility of the work that we publish. This form provides structure for consistency and transparency in reporting. For further information on Nature Research policies, see our Editorial Policies and the Editorial Policy Checklist.

Statistics

For all statistical analyses, confirm that the following items are present in the figure legend, table legend, main text, or Methods section.

n/a	Confirmed	
☐	☒	The exact sample size (*n*) for each experimental group/condition, given as a discrete number and unit of measurement
☒	☐	A statement on whether measurements were taken from distinct samples or whether the same sample was measured repeatedly
☐	☒	The statistical test(s) used AND whether they are one- or two-sided<br>*Only common tests should be described solely by name; describe more complex techniques in the Methods section.*
☒	☐	A description of all covariates tested
☒	☐	A description of any assumptions or corrections, such as tests of normality and adjustment for multiple comparisons
☐	☒	A full description of the statistical parameters including central tendency (e.g. means) or other basic estimates (e.g. regression coefficient) AND variation (e.g. standard deviation) or associated estimates of uncertainty (e.g. confidence intervals)
☐	☒	For null hypothesis testing, the test statistic (e.g. *F*, *t*, *r*) with confidence intervals, effect sizes, degrees of freedom and *P* value noted<br>*Give P values as exact values whenever suitable.*
☒	☐	For Bayesian analysis, information on the choice of priors and Markov chain Monte Carlo settings
☒	☐	For hierarchical and complex designs, identification of the appropriate level for tests and full reporting of outcomes
☐	☒	Estimates of effect sizes (e.g. Cohen's *d*, Pearson's *r*), indicating how they were calculated

Our web collection on statistics for biologists contains articles on many of the points above.

Software and code

Policy information about availability of computer code

Data collection	Exome and whole transcriptome sequencing was conducted on the HiSeq2000 or 2500 platform (Illumina Inc, California, USA) by the Princess Margaret Genomic Centre and Translational Genomics Laboratory in Toronto, Canada. Natera Inc (San Carlos, USA) designed and performed and bespoke mutation panels for ctDNA detection.
Data analysis	WES alignment: Burrows-Wheeler Alignment (version 0.7.12)<br>RNAseq alignment: STAR (version 2.4.2a)<br>RNAseq analysis: RSEM (version 1.3.0), sva (version 3.36), DESeq2 (version 3.11), CIBERSORT (version 1.06, https://cibersort.stanford.edu/), ComBat (gsva(veresion 3.11))<br>QC: Picard (version 2.10.9), RNA-seQC (version 1.1.8), NGSCheckMate (https://parklab.github.io/NGSCheckMate/)<br>Mutation analysis: Haplotypecaller (GATK version 4.0.5.1), Mutect2 (GATK version 3.8), Mutect (version 1.1.4), Strelka (version 1.0.14), Varscan2 (version 2.4.2), Vardict (version 1.5.8), Variant Effect Predictor (version 92), deconstructSigs (version 1.8.0), Sequenza (version 3.0.0)<br>HLA analysis: PolySolver (version 1.0, https://hub.docker.com/r/sachet/polysolver/tags/), HLA-LOH (https://bitbucket.org/mcgranahanlab/lohhla/src/master/)<br>MSI analysis: mSINGs (https://bitbucket.org/uwlabmed/msings.git)<br>General software: R (version 3.3.1)<br>ctDNA analysis: The Signatera ctDNA system is a proprietary assay developed and trade-marked by Natera Inc. (San Carlos, USA)

For manuscripts utilizing custom algorithms or software that are central to the research but not yet described in published literature, software must be made available to editors and reviewers. We strongly encourage code deposition in a community repository (e.g. GitHub). See the Nature Research guidelines for submitting code & software for further information.

Data

Policy information about availability of data

All manuscripts must include a data availability statement. This statement should provide the following information, where applicable:

- Accession codes, unique identifiers, or web links for publicly available datasets
- A list of figures that have associated raw data
- A description of any restrictions on data availability

Anonymized patient normal, tumor exome and RNAseq bam files generated in this study have been deposited in the European Genome-phenome Archive repository under accession code EGAS00001003280 [https://ega-archive.org/studies/EGAS00001003280]. The datasets are available under restricted access in compliance with patient consent for data sharing, access can be obtained by approval from the University Health Network data access committee (Contact person: Natalie Stickle, Email: natalie.stickle@uhn.ca). The processed mutation data is available at EGAD00001006569. A redacted version of the clincial trial study protocol is provided in Supplementary Note 1. The publically available datasets (Broad MSS mixed solid tumors10, UMich MET50011, and MSKCC-IMPACT IO study12) used in this study are available via the cBioportal dataportal [https://www.cbioportal.org/]. Source data for all figures and supplementary figures are provided with this paper.

Field-specific reporting

Please select the one below that is the best fit for your research. If you are not sure, read the appropriate sections before making your selection.

☒ Life sciences ☐ Behavioural & social sciences ☐ Ecological, evolutionary & environmental sciences

For a reference copy of the document with all sections, see nature.com/documents/nr-reporting-summary-flat.pdf

Life sciences study design

All studies must disclose on these points even when the disclosure is negative.

Sample size	Sample size calculation was not performed. Sample size was chosen based on data availability based on maximum patient enrollment and budgetary considerations.
Data exclusions	WES data excluded from final dataset used when genotypes were mismatched between normal/tumor pairs and tumors < 20% cancer cell fraction.
Replication	No replication was performed due to sample size and biospecimen availability. Sequencing and ctDNA measurements were performed once on each sample and not repeated.
Randomization	There is no randomization as part of this study. Patients were enrolled into one of five sub-cohorts: (A) Squamous Cell Cancer of Head and Neck; (B) Triple Negative Breast Cancer; (C) Epithelial Ovarian Cancer Type II; (D) Metastatic Melanoma; or (E) Mixed Advanced Solid Tumors. Control for covariates were not applicable as analyses were performed on the whole cohort with all tumor types to address pan-cancer research aims.
Blinding	Natera was blinded to the clinical data including PFS, OS, and objective response while conducting the ctDNA measurements. Tissue processing, PD-L1 IHC, exome and RNA-seq and data proessing were were also performed without knowledge of patient outcomes. Blinding was not applicable for analyses involving comparison of patient groups based on clinical outcome groupings.

Reporting for specific materials, systems and methods

We require information from authors about some types of materials, experimental systems and methods used in many studies. Here, indicate whether each material, system or method listed is relevant to your study. If you are not sure if a list item applies to your research, read the appropriate section before selecting a response.

Materials & experimental systems

n/a	Involved in the study	
☐	☒	Antibodies
☒	☐	Eukaryotic cell lines
☒	☐	Palaeontology and archaeology
☒	☐	Animals and other organisms
☐	☒	Human research participants
☐	☒	Clinical data
☒	☐	Dual use research of concern

Methods

n/a	Involved in the study	
☒	☐	ChIP-seq
☐	☒	Flow cytometry
☒	☐	MRI-based neuroimaging

Antibodies

Antibodies used	PD-L1 immunohistochemistry staining and assessment was conducted by QualTek Molecular Laboratories (Newtown, PA, USA) using monoclonal antibody clone 22C3 (Merck) at a concentration of 2ug/mL
Validation	QualTek Molecular Laboratories, along with Merck & Co Inc (Merck Canada), has validated the IHC assay for PD-L1 using mAb 22C3 for testing on formalin-fixed paraffin-embedded (FFPE) tissues (Dolled-Fillhart M. et al., Archives of Pathology & Laboratory Medicine: Nov. 2016, No. 11, pp. 1259-1266; https://dx.doi.org/10.5858/arpa.2015-0544-OA).

Human research participants

Policy information about studies involving human research participants

Population characteristics	Patients greater than 18 years old with advanced solid tumors and available archived or baseline tumor tissue provided informed consent were enrolled in INSPIRE. Patients were enrolled into one of five sub-cohorts: (A) Squamous Cell Cancer of Head and Neck; (B) Triple Negative Breast Cancer; (C) Epithelial Ovarian Cancer Type II; (D) Metastatic Melanoma; or (E) Mixed Advanced Solid Tumors. Participants were required to have measurable disease, ECOG status 0-1, and adequate organ function. Participants could not have had prior anti-PD1/PDL1/PDL2 therapy or active immunodeficiency, auto-immune disease, pneumonitis, active tuberculosis, or active central nervous system metastases. Patient were mainly female (62%) with ages range from 21 to 81 years old (median 59 years old) at the time of enrollment.
Recruitment	Patients were recruited at clinics in Princess Margaret Cancer Centre (University Health Network), a research hospital in Toronto, Ontario. As patients were expected to allow on-treatment biopsies and provide research blood samples, the recruited population likely represented on average a healthier and better prognosis group than an average clinical presentation off study. This self-selection bias could result in altered effect sizes of prognostic and predictive biomarkers such as change in ctDNA, immune infiltration and gene-expression signatures.
Ethics oversight	The clinical trial was approved by the Research Ethics Board at University Health Netwrok in Toronto, Canada. The trial was conducted in accordance with the principles of Good Clinical Practice, the provisions of the Declaration of Helsinki, and other applicable local regulations. All patients gave their written informed consent.

Note that full information on the approval of the study protocol must also be provided in the manuscript.

Clinical data

Policy information about clinical studies

All manuscripts should comply with the ICMJEguidelines for publication of clinical research and a completedCONSORT checklist must be included with all submissions.

Clinical trial registration	NCT02644369
Study protocol	The full protocol is available from the authors upon reasonable request. A redacted protocol is provided in Supplementary Note 1.
Data collection	Patients recruitment and data collection was conducted at Princess Margaret Cancer Centre from March 21, 2016 to May 9, 2018. Data collection cutoff was July 18, 2019
Outcomes	Survival outcomes of the study was defined by progression free survival (PFS) and overall survival (OS). Clinical outcomes was determined by RECIST criteria and clinical benefit was defined as patients with complete or partial response or stable disease greater than 18 weeks.

Flow Cytometry

Plots

Confirm that:

- [x] The axis labels state the marker and fluorochrome used (e.g. CD4-FITC).
- [x] The axis scales are clearly visible. Include numbers along axes only for bottom left plot of group (a 'group' is an analysis of identical markers).
- [x] All plots are contour plots with outliers or pseudocolor plots.
- [x] A numerical value for number of cells or percentage (with statistics) is provided.

Methodology

Sample preparation	Pooled tumor core biopsies or tissue samples were minced into 2-4mm3 fragments and digested with the gentle MACS dissociator (Miltenyi, Catalog #130–093-235) and the human tumor dissociation kit (Miltenyi, Catalog #130–095-929). Cells were stained for immune markers of interest.<br>Table including immune markers of interest, catalogue numbers, and dilutions are presented in Supplementary Table 3

Instrument	Data were acquired using a 5-laser LSR Fortessa X-20 (BD, Mississauga, Ontario, Canada).
Software	Immunophenotyping data were analyzed using FlowJo v10 (Treestar, Ashland, Oregon, USA).
Cell population abundance	Not relevant to the study. No cell sorting was performed.
Gating strategy	Gating strategies for CD3 T, CD8 T, and CD19 B cells are presented in Supplementary Figure 9A

☒ Tick this box to confirm that a figure exemplifying the gating strategy is provided in the Supplementary Information.
